# Informal carers’ experiences of mental health services regarding supporting those they care for and getting support for their own wellbeing: A systematic umbrella review of qualitative evidence

**DOI:** 10.1371/journal.pmen.0000740

**Published:** 2026-10-15

**Authors:** Xiaoye Liu, Phoebe Barnett, Patrick Nyikavaranda, Laura Burley, Brynmor Lloyd-Evans

**Affiliations:** Division of Psychiatry, Faculty of Brain Sciences, University College London, London, United Kingdom; PLOS: Public Library of Science, UNITED KINGDOM OF GREAT BRITAIN AND NORTHERN IRELAND

## Abstract

Informal carers play a vital role in supporting individuals with mental health difficulties. To provide an overarching synthesis of available evidence, this umbrella review aimed to synthesise qualitative review evidence on informal carers’ (including family members, friends, or other unpaid supporters) experiences of mental health services regarding supporting the individuals with mental health difficulties they care for and seeking support for their own wellbeing. Following the Joanna Briggs Institute (JBI) methodology, a meta-aggregative umbrella review was conducted. Five databases were searched up to 9th May 2025: CINAHL, MEDLINE, PsycInfo, the Cochrane Library, and Epistemonikos. The quality of primary systematic reviews was assessed using a framework adapted from the work of SmithBattle and colleagues. Findings were extracted from eligible reviews and aggregated into categories, from which higher-order synthesised findings were developed. Twelve eligible qualitative reviews were identified covering a wide range of mental health service contexts. Six synthesised findings were identified: (1) emotional complexity in the caregiving journey; (2) recognition and coping with their loved ones’ mental health difficulties; (3) multiple barriers to accessing mental health support; (4) persistent responsibilities and roles in supporting service users; (5) complex experiences of engaging with services; and (6) relational strain during the caregiving process. While some carers reported positive experiences, most described exclusion, emotional distress, and structural barriers to support. This review highlights the urgent need for mental health services to recognise, involve, and support informal carers.

## Introduction

In the UK, around 13% of the approximately 1.5 million informal carers (e.g., family members, friends, or significant others) play a vital role in caring for individuals with mental health difficulties [1,2]. The carers often assist with the individuals’ daily living and functioning needs, provide emotional support, manage crises, facilitate medical engagement, and complement professional services [3–5]. A multinational survey estimated the monetary value of the care provided by each carer, which ranged from €34,960 to €125,412 per year, depending on living arrangements and valuation methods [6]. The benefits of carers’ involvement in caring for individuals with mental health difficulties are well-established. Increased carer involvement in treatment has been associated with service users’ reduced psychiatric symptoms, shorter durations of hospitalisation [7], and longer maintenance of remission [8].

While carers’ involvement is beneficial to service users, the caregiving role often places significant practical and psychological burdens on carers themselves, resulting in poorer mental health and higher psychiatric symptoms compared to non-caregivers [9]. The caring roles demand significant time, financial resources, and emotional energy. For instance, informal carers of individuals with mental health difficulties provided an average of 43.42 hours of care per week, rising to 65.41 hours for co-resident carers [6]. In addition to the practical burden, the emotional toll of caregiving is highlighted. Research has consistently shown that many informal carers experience reduced quality of life, with high levels of stress, anxiety, fear, and social isolation, although the degree and nature of experiences can vary depending on individual, cultural, and systemic contexts [9–13]. At the same time, carers are frequently marginalised within mental health systems [14]. The complexities of navigating mental health services and the lack of recognition of carers’ needs and corresponding support, combined with stigma, exacerbate carers’ difficulties [15]. Their involvement is often undervalued or even actively excluded by service providers due to concerns about confidentiality, risk, or professional boundaries [14,16]. These dynamics can create tension between carers and mental health professionals, further complicating their caregiving experience and amplifying psychological challenges [15].

Understanding carers’ experiences of accessing and engaging with mental health services regarding supporting the service users and getting support for their own wellbeing is essential for informing policy development and driving service adaptations to better support informal carers and promote their wellbeing, which is also recognised and encouraged by policy makers [17]. In response to this need, there has been a growing body of qualitative research exploring carers’ experiences of mental health services. Correspondingly, a number of qualitative systematic reviews and syntheses have been conducted to consolidate this evidence and provide broader insights into carers’ experiences across diverse contexts, such as inpatient services [15], respite care services [18], psychosis interventions [19], and others. However, to date, no comprehensive synthesis has integrated these diverse experiences into a single, high-level evidence base. Given the growing amount of qualitative systematic reviews in this area, an umbrella review that systematically identifies, evaluates, and synthesises evidence from existing review-level literature is warranted to collate and summarise existing evidence to inform the development of more inclusive, effective, and holistic caring systems. Thus, this study aims to conduct an umbrella review of review-level qualitative evidence on informal carers’ experiences of mental health services regarding supporting the individuals with mental health difficulties they care for and getting support for their own wellbeing.

## Methods

The umbrella review was primarily guided by the JBI Manual for Evidence Synthesis, which provided comprehensive guidance on all stages of the umbrella review process, including protocol development, systematic literature selection, quality appraisal, data extraction, evidence synthesis, and certainty assessment [20]. Data synthesis used a meta-aggregative approach, based on JBI guidance on meta-aggregation and adapted for review-level analysis [20]. The protocol was pre-registered in the publicly accessible PROSPERO database on July 20, 2025 (PROSPERO 2025 CRD420251046003). Ethical approval was not required for this study as it was an umbrella review of existing literature.

### Eligibility criteria

This umbrella review included published systematic reviews, other types of literature reviews, or qualitative meta-syntheses with distinguishable qualitative findings specific to the experiences of informal carers (e.g., family members, friends, or unpaid individuals) in accessing and engaging with mental health services in any setting (e.g., community, inpatient, outpatient) through their caring role for individuals with mental health diﬃculties. The reviews were eligible only if they searched at least two databases for qualitative or mixed-methods studies, and if the findings from the primary qualitative studies were distinguishable and explicitly identified in tables or text. There were no restrictions on language, geographic location, or date of the reviews.

Reviews on informal carers’ experience of individuals with neurocognitive or neurodevelopmental disorders were excluded. Reviews focused on drug, alcohol, and smoking services, or speciﬁc, discrete interventions within mental health services were excluded. Reviews on the general experience of informal carers of individuals with mental health diﬃculties, that are not primarily and explicitly focused on carers’ experiences of mental health services, were also excluded to maintain a clear focus for the current review.

### Search strategy and study selection

A literature search was conducted across five databases on 9th May 2025: CINAHL, MEDLINE, PsycInfo, the Cochrane Library, and Epistemonikos. The databases provided a comprehensive coverage of medical, psychological, nursing, and evidence-based review literature relevant to mental health and caregiving. The complete query strings can be found in **S1 Appendix** **(A: Search Strategy)**.

The references were imported into Covidence, deduplicated, and screened [21]. Two independent reviewers (XYL and LB) discussed the eligibility criteria and conducted a pilot screening exercise to ensure a consistent understanding and application of the eligibility criteria. Screening of titles and abstracts was undertaken by one independent reviewer (XYL), consistent with Cochrane guidance that initial title and abstract screening may be conducted by a single reviewer [22]. A second reviewer (LB) double-screened 285 results, approximately 10% of the results screened at the title and abstract level, to ensure consistency. 35 discrepancies were identified and resolved through discussion, achieving strong agreement (κ = 0.88). Subsequently, two independent reviewers (XYL and LB) assessed potentially eligible reviews in full text. During the whole selection process, disagreements were resolved through discussion or consultation with a third independent reviewer (BL).

### Data extraction

Two independent reviewers (XYL and LB) manually extracted data from each eligible review using a structured, pre-deﬁned table adapted from the JBI Data Extraction Form for Review for Systematic Reviews and Research Syntheses using Microsoft Excel [20]. The two reviewers first extracted data from three reviews, compared results to standardise procedures, and then completed the extraction from the remaining reviews. Discrepancies were resolved through discussion or consultation with a third reviewer (BL).

Extracted information included: citation details, review type, analysis technique, sources searched, the search date range, details of original qualitative studies (number and types), context, review objectives, phenomena of interest, carer participant characteristics, and findings on informal carers’ experiences of mental health services, along with verbatim excerpts (i.e., text copied directly from the included reviews without modification, representing the original descriptions of findings reported by the review authors) and selected quotes from the source reviews.

### Quality appraisal

The methodological quality of the included reviews was appraised using the criteria adapted from SmithBattle et al.’s umbrella review [23], covering six domains: search criteria, review type, search process, findings extraction, identification of findings, and synthesis methods. The tool was selected for its appropriateness for qualitative umbrella reviews and its thorough coverage of key methodological domains. Two independent reviewers assessed each included review against the six criteria, rating them as either “yes” (criterion fulfilled) or “no” (criterion not fully met). Discrepancies between reviewers were resolved through discussion to reach a consensus.

Unlike SmithBattle et al.’s original method, which reported only “yes” or “no” ratings, a three-level quality classification (high, moderate, low) was then applied to improve the interpretability of the ratings and to lay the foundation for certainty assessment. Reviews meeting five or more criteria were rated as high quality, those meeting three or four criteria as moderate quality, and those meeting fewer than three as low quality, whose thresholds were informed by logic and practice applied in dependability assessment in the JBI ConQual framework.

### Assessment of overlap between primary studies

To assess the extent of overlap between primary studies included across the reviews, a citation matrix was constructed, and the Corrected Covered Area (CCA) was calculated [24]. Overlap among primary studies across reviews may result in the over-representation of certain evidence and disproportionately influence the synthesis. The CCA provides an estimate of the degree of overlap by calculating the proportion of repeated primary study occurrences relative to the total number of study occurrences across included reviews [24]. Higher CCA values indicate greater overlap and a greater potential for duplicated evidence to influence the interpretation of findings. CCA values below 5% are generally considered to indicate slight overlap, 5–10% moderate overlap, and above 10% high overlap [25].

### Data synthesis

Findings from all included reviews were synthesised using an adapted meta-aggregative approach, which forms credible synthesised findings through aggregation with deliberate efforts to minimise re-interpretation of original review-level synthesis [20]. Extracted findings were assessed as unequivocal, equivocal or unsupported, based on how clearly they were based on direct quotations or observations. Unequivocal and equivocal findings were grouped into categories based on similarity in meaning and representation of the carers’ experience. Each category was described in a clear, concise narrative that preserved the original intent of the primary review authors. Higher-order, synthesised findings were then developed by integrating two or more related categories. These findings were formulated as descriptive labels accompanied by summary statements. Narrative presentations supported by data excerpts or quotations were provided, allowing for a nuanced and meaningful representation of carers’ experiences. A summary table was provided, listing synthesised findings and showing which categories contributed to each synthesised finding.

### Assessment of the confidence of synthesised findings

To facilitate interpretation of the synthesised findings, an assessment of the breadth and robustness of the supporting evidence was undertaken. As no established framework exists for assessing confidence in synthesised findings in qualitative umbrella reviews, a narrative assessment was conducted using review-level information, considering the methodological quality of the included reviews, quality and adequacy of support, and breadth and diversity of the supporting evidence. Specifically, for each synthesised finding, we examined: 1) The methodological quality of the contributing reviews; 2) the extent to which the individual, extracted findings were well supported by primary evidence reported within the included reviews (e.g., links to primary qualitative findings or participant quotations), rated as unequivocal (i.e., clearly supported by direct and unambiguous evidence), equivocal (i.e., supported but open to some interpretation), or not supported; 3) the degree of primary study overlap across contributing reviews; 4) the number of contributing extracted findings and reviews to each category and synthesised finding; and 5) the diversity of mental health service contexts and geographical settings represented by the contributing evidence.

## Results

### Search results and study characteristics

The database search identified 3,746 records. After removal of 1,108 duplicates, 2,638 records underwent title and abstract screening, of which 2,542 were excluded. 96 full-text reviews were assessed for eligibility, and 84 were excluded. The reasons for exclusion were an ineligible phenomenon of interest (n = 27), an ineligible population (n = 21), an ineligible review design (n = 9), and the reviews were on informal carers’ experiences in general rather than experiences of accessing or engaging with mental health services (n = 27). The records excluded at the full-text screening stage were presented in **S1 Appendix** **(B: Records excluded at full text)** with reasons for their exclusion to ensure transparency. A total of 12 eligible reviews were identified and included in the current review. The whole study selection process was demonstrated in the PRISMA flow diagram in Fig 1.

**Fig 1:**
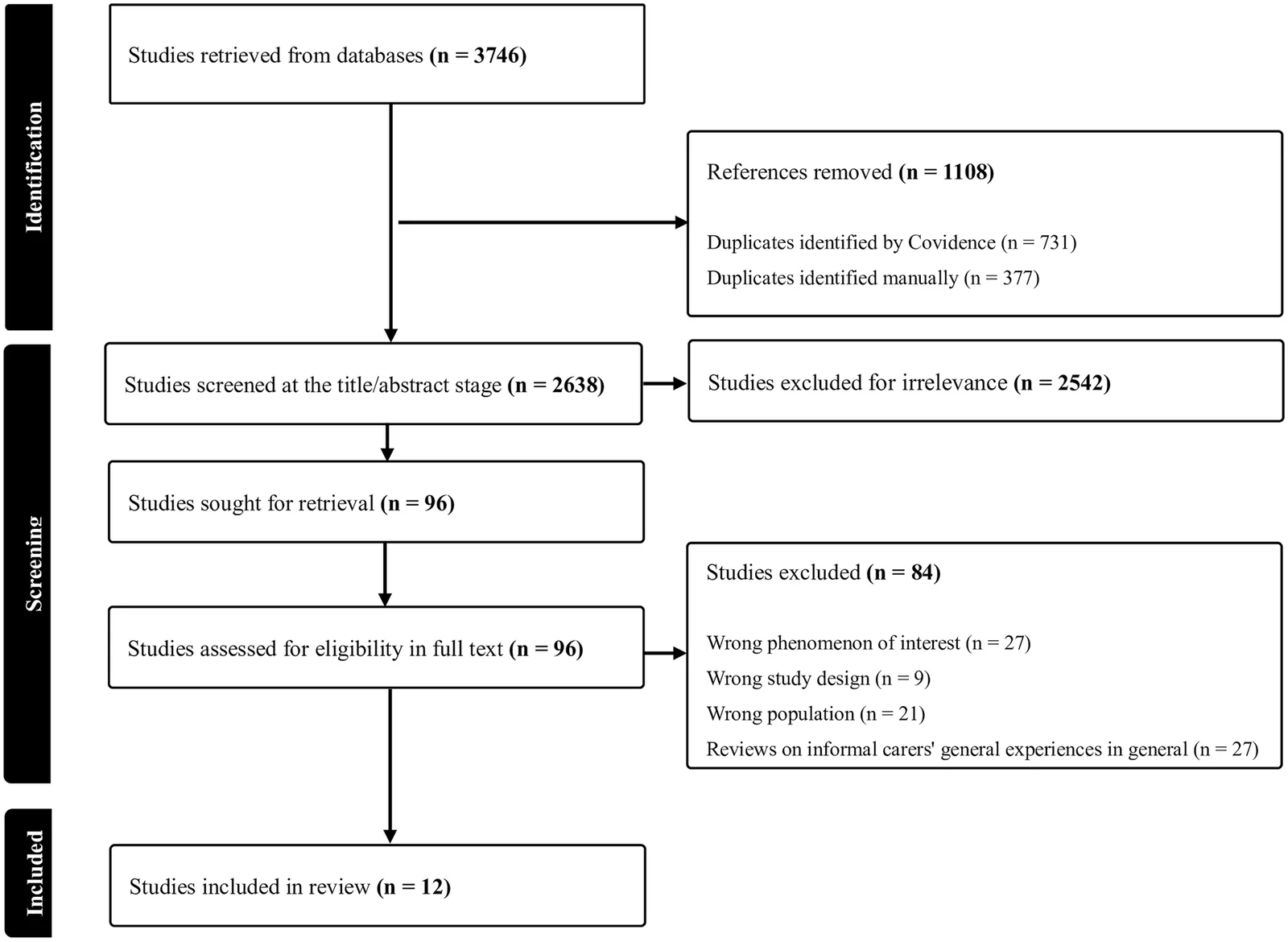
PRISMA flow diagram.

The key characteristics of included reviews are reported in **Table 1**. The included reviews were published between 2013 and 2024. Although the search imposed no language restrictions, all included reviews were published in English. Non-English records were identified but excluded because they did not meet the eligibility criteria. Most included reviews were systematic reviews and qualitative meta-syntheses (n = 10), while there was one integrative literature review [18] and one narrative review [26]. Most qualitative syntheses were conducted thematically (n = 8), while others utilised meta-ethnography (n = 2) [27,28], aggregation (n = 1) [19], and narrative synthesis (n = 1) [26]. The number of original qualitative studies on carers included in each review ranged from 5 [18,29,30] to 25 [31]. The types and context of the original qualitative studies were not included in the study characteristics table due to inconsistent reporting at the review level. Most reviews applied no geographical restrictions, except for one review focused exclusively on Australian studies [18]. Where the socio-economic context of primary studies was reported, the majority originated from high-income countries. The objectives of the reviews were generally to synthesise the qualitative evidence on informal carers’ experiences of mental health services. The mental health service settings and conditions explored across reviews were wide-ranging. Eleven included reviews focused on mental health services primarily aimed at service users, including services regarding compulsory admission [30,32,33], inpatient care [15,29], psychosis [19,26,27], perinatal mental health [28], anorexia nervosa [31], and forensic mental health [34]. One review addressed respite care services, which explored carers’ experiences of using the services that were primarily for them, providing temporary relief by housing service users for short periods [18]. Carer participant characteristics were also not presented in a table in this umbrella review since only a few reviews synthesised and reported the information at a review level. Participant descriptives were synthesised and reported in only three of the included reviews, where carers were often family members and predominantly adult females [31,33,34]. While some reviews reported the total number of carers included, ranging from 100 [30] to 357 [31], others did not specify the total counts, reflecting variability in reporting standards. Detailed demographic breakdowns (e.g., age and ethnicity) were rarely available and inconsistently presented.

**Table 1:** Study characteristics and quality appraisal.

Citation details	Type of review	Analysis technique	Sources searched	Search date	N of studies^a^	Phenomena of interest	Quality appraisal (Y = Yes; N = No)						
							1^b^	2^c^	3^d^	4^e^	5^f^	6^g^	QR^h^
AbouSeif et al., 2022 [15]	Systematic review and thematic synthesis	Thematic analysis	MEDLINE, PsycINFO, Embase, and CINAHL	Inception to February 2021	12	Informal carers’ experiences of adult (18+) mental health inpatient care	Y	Y	Y	Y	Y	Y	High
Almoaber et al., 2024 [18]	Integrative literature review	Thematic analysis	CINAHL, MEDLINE, PsycINFO, and Scopus	Between January 2005 and May 2023	5	The experiences of 18–65 years who were caring for a person aged 18 years or over with a primary diagnosis of mental illness regarding respite care services	Y	Y	Y	N	N	Y	Moderate
Bartl et al., 2024 [32]	Systematic review and qualitative meta-synthesis	A two stage process, informed by thematic synthesis	MEDLINE, PsycINFO, HMIC and Embase, and the Social Sciences Citation Index	Between January 2018 and March 2023	8	The experiences of compulsory admission or legally detained to a psychiatric hospital	Y	Y	Y	Y	Y	Y	High
Cairns V.A. et al., 2015 [27]	Systematic review and qualitative meta-synthesis	Noblit and Hare’s process of meta-ethnography	PsycINFO, AMED, CINAHL, Web of Science, Medline, and PubMed	From 1806 to 2013	13	Family members’ experience of seeking help for first-episode psychosis on behalf of a loved one	Y	Y	Y	N	Y	Y	High
Dawson et al., 2013 [19]	Systematic review	Aggregation and meta-synthesis	CINAHL, PubMed, Scopus, PsycINFO, Embase, Mednar, ProQuest Dissertations and Theses, Trove	From January 1990 to August 2012	22	Carers’ experience of a relative with first-episode psychosis, and in particular, their experience preceding and of seeking help from both informal (family and friends) and formal (services) sources.	Y	Y	Y	Y	Y	Y	High
Lever Taylor et al., 2018 [28]	Systematic review and qualitative meta-synthesis	An approach based on meta-ethnography	PsycINFO, CINAHL, Embase, HMIC, and Medline	Inception up to June 2017	20	Women’s partners’ experiences and views of perinatal mental health care	Y	Y	Y	Y	Y	Y	High
Oketah et al., 2023 [31]	Systematic review and meta-synthesis	Thematic analysis	Medline, PsychINFO, CINHAL, Embase, Cochrane Library, and Web of Science	Between January 1980 and August 1, 2023	25	Parents’ views on the treatment and recovery process of anorexia nervosa in their adolescent child.	Y	Y	Y	N	Y	Y	High
Reupert et al., 2023 [29]	Systematic review	Thematic synthesis	CINAHL Plus, PsycINFO, ProQuest, MEDLINE, PubMed, and Scopus	Between 2004 and 13th January 2022	5	The experiences of parents and other family members when a parent is hospitalised for their mental illness.	Y	Y	Y	Y	Y	Y	High
Rodell & Parry, 2024 [26]	Narrative review	Narrative synthesis	PsycINFO and PubMed. Reference lists were cross-checked.	From November 2021 to February 2022	12	The experiences of supporting those under 18 years old reporting symptoms associated with the psychosis spectrum or unusual sensory experiences.	Y	Y	Y	N	Y	Y	High
Stuart et al., 2020 [33]	Systematic review	Thematic synthesis	Medline, PsycINFO, HMIC, Embase, and Social Sciences Citation Index	Search in January 2018, limited to papers published after 1983	23	Carers’ experiences of and views about assessment for involuntary admission and subsequent detention	Y	Y	Y	Y	Y	Y	High
Sugiura et al., 2020 [30]	Systematic review and meta-synthesis	Thematic analysis	Academic Search Complete, CINAHL, ERIC, MEDLINE, PsycINFO, Ichushi, SveMed+, EBSCO, Enfispo, IBECS, LILACS, Psicodoc, and SciELO	From 2008 to 2018	5	The experiences of involuntary psychiatric admission among adults	Y	Y	Y	N	Y	Y	High
Vestphal et al., 2023 [34]	Systematic review and qualitative evidence synthesis	Thematic synthesis	CINAHL, Medline, PsycINFO, and Scopus	From 2014 to 2022	13	Family carers’ experiences in forensic mental health care	Y	Y	Y	N	Y	Y	High

^a^The number of original qualitative studies on carers included in each primary review

^b^Criterion 1: Are the search criteria clearly identified and appropriate for the aim?

^c^Criterion 2: Is the type of review identified?

^d^Criterion 3: Is the search process clearly described in a figure or text?

^e^Criterion 4: Are procedures for data extraction, with what was extracted from primary studies, explicitly described?

^f^Criterion 5: Are findings of primary qualitative studies clearly identified?

^g^Criterion 6: Are findings from primary studies synthesised?

^h^QR is short for quality rating.

The quality appraisal of included reviews is also reported in **Table 1**. Eleven reviews were rated as high quality for satisfying at least five out of the six appraisal criteria. One review was rated as moderate quality for satisfying only four criteria [18]. All included reviews clearly reported their search criteria, review type, search process, and synthesised primary findings. However, one review did not clearly identify primary findings [18], and six lacked detailed information on data extraction processes [18,26,27,30,31,34]. These gaps suggest some limitations in the transparency and reproducibility of the selection and interpretation of findings in a minority of reviews.

The lists of primary studies across the included reviews were cross-checked to assess the potential overlap. Across the 12 included reviews, there were 168 total occurrences of primary studies, representing 143 unique primary studies. Among these, 14 primary studies were included in two reviews, 4 were included in three reviews, and 1 was included in four reviews. The degree of overlap was quantified using the Corrected Covered Area (CCA) [24]. It was 1.6%, indicating slight overlap. This suggests that the included reviews were based largely on independent bodies of primary qualitative evidence and that repeated inclusion of the same primary studies in multiple reviews was unlikely to have materially influenced the synthesis by disproportionately representing particular findings.

### Meta-aggregative synthesis

Six overarching synthesised findings were identified, derived from 105 individual findings that were subsequently aggregated into a set of 20 categories. 6 findings from the included reviews were not incorporated into categories due to uniqueness or lack of support from primary data. The structure of the synthesised findings is presented in **Table 2**, indicating which categories contributed to each synthesis. As for the narrative presentations, the opening paragraph under each synthesised finding provided a brief, overarching summary of that synthesised finding. The brief summary was followed by narrative descriptions of each contributing category, with each subsequent paragraph corresponding to one category and elaborating on the specific aspects of carers’ experiences represented within it. Where appropriate, data excerpts or quotations reported in the included reviews are presented to support the narrative synthesis and provide contextual richness while remaining faithful to the original review findings. Findings extracted from each included review are presented in **S1 Appendix** **(C: Findings extracted from each review Tables 1-12)**. Findings contributing to each category, including verbatim excerpts from the source reviews, are presented in **S1 Appendix** **(D: Findings contributing to each category Tables 1-20)**.

**Table 2:** Organisation of the synthesised findings and supporting categories.

Synthesised finding	Category
Emotional complexity in the caregiving journey	High emotional distress during the build-up to help-seeking and accessing services
	Mixed and complicated emotional responses to service involvement
	Uncertainty and guarded hope about the future
Recognition and coping with their loved ones’ mental health difficulties	Early recognition, reactions, and management
	Boosted help-seeking after reaching “crisis point”
Multiple barriers for carers in accessing mental health support	Systematic barriers to accessing appropriate mental health support
	The uneasy and continuous process of sourcing services
	Lack of information and knowledge
	Complex personal and relational barriers
Persistent responsibilities and multiple roles in supporting service users	Experiential knowledge and potential to help
	A strong sense of responsibility and persistent caring roles
Complex experiences of engaging with mental health services	Dissatisfaction and distrust
	Neglect of needs and lack of adequate support
	Exclusion from care and decision-making
	Unmet need for information and communication
	Limitations of current mental health service models
	Expectations of health professionals and services
	Variations in views and experiences
Relational strain during the caregiving process	Strained relationships between carers and service users
	Unsatisfactory relationships between carers and health professionals
Persistent responsibilities and multiple roles in supporting service users	Experiential knowledge and potential to help
	A strong sense of responsibility and persistent caring roles
Complex experiences of engaging with mental health services	Dissatisfaction and distrust
	Neglect of needs and lack of adequate support
	Exclusion from care and decision-making
	Unmet need for information and communication
	Limitations of current mental health service models
	Expectations of health professionals and services
	Variations in views and experiences
Relational strain during the caregiving process	Strained relationships between carers and service users
	Unsatisfactory relationships between carers and health professionals

#### Synthesised finding 1: Emotional complexity in the caregiving journey.

Informal carers of people with mental health difficulties underwent a complex emotional journey marked by intense distress before accessing services, mixed and conflicting feelings during service involvement, and ongoing uncertainty and guarded hope about the future. These experiences reflected the psychological burden of caregiving shaped by uncertainty, fear, guilt, stigma, responsibility, and fluctuating hope across different stages of the care trajectory.

Carers’ intense and difficult emotional experiences during the build-up to seeking and accessing mental health services were consistently described. Preceding formal engagement with services, carers were often the first to recognise early changes in their loved ones and manage escalating symptoms. The process was marked by overwhelming feelings of confusion, distress, and helplessness. Carers were often among the first to recognise changes in their loved ones, upon which they “experienced a sense of ‘not knowing’” [27]. Some carers felt “distressed, embarrassed and confused” when they confronted their loved ones’ symptoms and behaviour changes [19]. Anxiety, fear, sadness, and exhaustion were also reported [19,32,34]. Meanwhile, the deterioration of service users was often a gradual process, which typically progressed over an extended period before attracting clinical attention. The build-up to the service users’ crisis was described as distressing and overwhelming [15]. Before the service users were accepted by mental health services, carers struggled to face and manage the evolving situations on their own. The continued and uncertain process caused strong emotional impacts on carers, leading to difficult feelings such as “powerlessness and frustration” [15], “high stress and hypervigilance” [32], “fear, sadness, helplessness, hopelessness, and isolation” [19]. At the same time, many carers sought help on behalf of their loved ones, which also led to frustration when health professionals failed to recognise the severity of the service users’ conditions [32], high distress, and sometimes even a sense of trauma [27].

After the service users entered mental health services, carers reported experiencing mixed feelings in response to service engagement. While some findings reported positive emotions, such as relief and hope, when carers felt their loved ones were supported and protected, more described ongoing distress and complex emotional experiences. Carers expressed uncertainty and powerlessness regarding the treatment process, self-blame for delayed help-seeking or for contributing to their loved ones’ mental health difficulties, and feelings of loneliness and isolation arising from strained relationships and the stigma surrounding mental health difficulties. Many carers reported some relief when they felt the service users were safe and under appropriate treatments [15,18,28,32,33]. For instance, “one carer emphasised ‘feeling peace of mind that their loved one was safe’”, as cited in one review [18]. Positive feelings such as gratitude and hope were also reported in a few reviews [15,33]. However, difficult emotions still appear to be prominent. Some carers reported distress and even trauma related to initiating coercive measures, undergoing assessments, and witnessing admission [32]. Some expressed personal uncertainty [32] and reported feeling worried due to limited knowledge about the illness and the treatment process [31]. Some reported struggling with guilt and self-blame for not recognising the conditions earlier, delaying help-seeking, or even believing they were “causing the illness” [31]. The self-blame further led to a sense of helplessness or powerlessness, as illustrated by cited statements such as “we’d failed as parents” and “I kind of think that nothing I do helps, really” [31]. Relationship strain during service engagement also adversely affected carers’ well-being. Distress could arise from disagreements with other family members about the appropriate course of action and conflicts between the service users’ wishes and what was considered to be in their best interests [32]. Some carers reported feelings of loneliness and expressed a desire for connection [29,31]. Increased caregiving responsibilities led to social disconnection, while stigma surrounding mental illness contributed to reduced support and greater peer blame [31]. Collectively, these findings revealed that while formal service engagement could alleviate some burdens, it introduced new emotional and social challenges for carers.

In terms of prospects, informal carers experienced enduring uncertainty and guarded hope regarding their loved ones’ recovery and future well-being, even after receiving professional treatment. Fear and frustration overshadowed optimism fostered by progress in the service user’s condition when setbacks occurred. For instance, carers expressed fear of relapse [31] and “experienced substantial anguish” when witnessing their children regress or relapse after making progress [26]. In addition, there was a tension between carers’ desire to be hopeful and the inclination to moderate it, as illustrated by the cited quotes: “I want to be optimistic but I feel maybe she can only be helped so much and then this will happen again. I fear she will backslide. My only hope is that she will be happy and enjoy life” [26]. The tension reflected an ongoing struggle to sustain hope amid the unpredictability of mental health recovery.

#### Synthesised finding 2: Recognition and coping with their loved one’s mental health difficulties.

Whereas the first synthesised finding focused on carers’ emotional responses to their loved one’s situations, the second focused on their actions and coping strategies upon recognition of changes in their loved ones. Initial denial, attributions to non-mental health causes, and interpretations based on their own knowledge and belief system influence their perceptions and corresponding management. Help-seeking to external and professional sources was often prompted by a “crisis point,” where carers’ coping capacity was overwhelmed.

Carers were often the first to notice changes in their loved ones, but their initial reactions varied. Some experienced denial or shock, while others struggled to make sense of the situation. Limited by their own knowledge and belief systems, carers’ misinterpretations were common, leading to delays in seeking help or turning to other sources rather than professional mental health services. Informal carers noticed issues with their loved ones through observing mood fluctuation, behavioural changes, and functional decline [19]. Upon recognition, some carers reacted with “denial and shock” [31]. Also, carers attempted to understand the changed presentations and make sense of the situation, mostly within their own knowledge and belief systems [27]. Their understanding and interpretations of the situations informed their subsequent type and timing of management. Some carers initially failed to recognise the seriousness of their loved ones’ conditions, as illustrated by the cited quote, “I thought it was a phase. I had no idea how serious,” which led to delayed help-seeking [31]. Others misattributed the early symptoms of mental health difficulties to other factors [19]. For instance, a mother misattributed her son’s mental health condition to supernatural causation, thus “forced her son to be exorcised in a Pentecostal church” [19]. After clarification of the presence of an illness, relatives sought to understand the possible causes or triggering factors of the condition [19].

These initial inactions in help-seeking were often resolved when carers reached what they perceived as a “crisis point,” where their personal coping resources became overwhelmed to manage the situation without professional mental health support [19,26,27]. This threshold triggered a sharp or urgent increase in seeking external support [19,26,27]. A review cited one carer’s reflection: “It took me quite a long time to understand that there was a mental issue there but it wasn’t until she started to become very ill. She actually had a crisis” [19].

#### Synthesised finding 3: Multiple barriers for carers in accessing mental health support.

After recognising their loved ones’ mental health difficulties and perceiving the necessity for professional support, carers played a crucial role in seeking help and accessing services on their behalf, often encountering numerous obstacles in the process. Carers faced multifaceted barriers when accessing mental health support, including systematic inadequacies, procedural difficulties, lack of information, and personal and relational challenges, often resulting in a prolonged, confusing, and emotionally consuming help-seeking process and delayed access to services.

Carers frequently encountered systematic inadequacies when attempting to access mental health support for their relatives, including a lack of appropriate and continuous services, restrictive entry criteria, and inadequate community care and pathways. While mental health services were generally lacking, tailored and appropriate services for specific populations, such as young people, were even more scarce, highlighting a gap in current services [26]. Also, carers voiced a lack of continuity in services, which was especially prominent in transitions between services and following inpatient discharge [26]. Strict and restrictive entry criteria further limited service availability. Some professional services were only accessible when individuals with mental health difficulties were “convincingly unwell, in crisis, at risk of harming themselves or others, or at least willing to engage in treatment” [19]. Respite care also had restrictive entry criteria concerning “the patient’s health condition, diagnosis, geographical location, age, length of the caring role and ethnicity” [18]. Community services and pathways to care were also described as insufficient and inadequate [32]. These barriers underscored the need for improved availability of existing services, as well as more flexible, inclusive, and holistic service models that addressed diverse needs and offered continuous support throughout different stages of care.

At the same time, sourcing mental health services was an uneasy and repeated process, which reflected procedural barriers to service access. Seeking help on behalf of the ill individuals often required multiple attempts and exhausting efforts [19,26,30]. For example, a review cited, “one parent described trying to convince health care professionals for over one year that her son needed help for his psychiatric illness” [19]. Difficulties in help-seeking led to delayed access to support and increased strain on carers. For instance, “families found it difficult to navigate the system to get support, they were frustrated with a delay in support, and they felt services were reactive rather than proactive” [30].

Carers’ lack of information and knowledge of mental health services further compounded the systematic and procedural barriers, creating confusion and hindered timely, informed decision-making. Many carers lacked clarity about available support [18], how and where to access it [26], and the procedures and legislation for interventions such as involuntary admission [18,26,30]. They expressed a need for more information and guidance from services to help them manage the situation, as illustrated by the cited quote: “There should be more support actually for me or actually tell me what I need to do to support him” [32].

Personal and relational barriers also hindered help-seeking and service access. Informal carers’ negative attitudes toward mental health services, often shaped by stigma or past negative experiences, and service users’ withdrawal or hostility toward carers’ help-seeking, both hindered access to services. Carers’ informal networks (e.g., family, friends, and community figures) and formal networks (e.g., healthcare professionals) may both obstruct help-seeking when dismissing difficulties or failing to provide appropriate support, though they facilitated service access with positive interactions. Informal carers’ own stigmatised attitudes toward mental health difficulties acted as initial barriers [26]. Carers’ negative past experiences of mental health services also led to “lasting distrust and reluctance to seek help from similar services” [26]. Service users may also play a role in impeding service access, being reluctant to engage in services when carers sought help [18,32]. They may withdraw from carers, mask their symptoms during assessments, or stay guarded [32]. Some may even be “hostile” to carers’ help-seeking efforts [26]. Carers’ informal networks, including family members, friends, and community figures such as religious leaders, can provide support to carers and may act as facilitators to service access [19]. The informal networks often contributed to “initial detection of problems, clarification of the seriousness of problems and encouragement to seek help” [19]. Meanwhile, another review highlighted that interactions within these networks could both facilitate help-seeking or hinder it when they normalised symptoms or dismissed difficulties [27]. The mediating effects of interactions were also evident when carers sought help from formal networks (e.g., mental health professionals, general practitioners, social workers, counsellors), as some interactions facilitated help-seeking and were regarded as helpful, while others acted as barriers [19,27,31]. Examples of barriers within formal networks included inappropriate treatment by professionals [27] and professional knowledge gaps, such as an inability to provide required treatment options [31].

#### Synthesised finding 4: Persistent responsibilities and multiple roles in supporting service users.

Even after navigating numerous challenges during the build-up to and accessing mental health services, carers did not disengage once service users were under care. While carers insisted on participating, they did have strong potential to facilitate treatment with their unique perspectives and in-depth knowledge of the service users. As carers felt a profound and persistent sense of duty in supporting the service users before, during, and after engaging in mental health services, they played important and multifaceted roles in supporting the service users.

Carers possessed in-depth knowledge and expertise of the service users’ behaviour and history through long-term, close observation and interactions, which was valuable for care and treatment [32,34]. Carers perceived their experiential knowledge as a facilitator in their involvement in care and treatment [34], strengthening their belief in both their ability to contribute and the necessity of their participation in treatment decision-making. Their expertise, however, was often unrecognised or dismissed by health professionals, upon which carers reported feelings of anger, disempowerment, and mistrust [34]. Despite these challenges, carers remained committed to supporting their loved ones.

Carers’ persistent efforts were driven by a strong sense of responsibility, which also led to significant burdens. They protected and supported their loved ones through various means, such as initiating admissions, mediating relationships between service users and professionals, and advocating for appropriate care when they perceived unfair treatment or neglect. Carers reported a strong sense of responsibility to the service users, persistently caring for them before, during, and after service engagement [32,34]. The responsibility led to prolonged, heavy burdens, some “to a point where they lived their lives with an almost constant focus on their ill family member” [34]. Carers remained vigilant to protect their loved ones and described themselves as “fighting from the outside inwards” [32]. They played multifaceted roles in protecting and supporting their loved ones. Some initiated admission, believing their actions provided protection [30,32]. Some mediated between service users and health professionals and advocated for the rights of the service users and appropriate care when they perceived unfair treatment or victimisation by healthcare systems or professionals [34]. For instance, a review cited that when perceiving a doctor’s dismissive response that downplayed the service user’s concerns, one carer challenged the doctor by stating, “I want you to repeat what you just said in front of a witness, because when she goes outa here and harms herself or somebody else, I’m taking you to court” [34].

#### Synthesised finding 5: Complex experiences of engaging with mental health services.

Regarding engagement with mental health services, carers expressed dissatisfaction with and distrust in the quality of care. They frequently reported experiencing neglect and inadequate support from services, exclusion from care and decision-making, and unmet needs for information and communication. Carers also raised concerns about inadequate staff competencies and service models that were inappropriate or culturally insensitive. Carers expected healthcare professionals to be empathetic, respectful, and compassionate. It was noticeable that carers expressed ambivalence about their involvement and support needs and reported mixed experiences with various treatment modalities.

Carers’ general comments on their experiences with service engagement in the included reviews were predominantly negative. They expressed dissatisfaction with and distrust in current services, including service delays, safety concerns, unmet service user needs, clinicians and other staff’s lack of competence, lack of respect, heavy-handed treatment, and an overreliance on medication [15,30–33]. While acknowledging that interventions such as detention were sometimes necessary, carers expressed profound misgivings, as some were already disappointed for failing to get earlier, preventive help when they were desperate to [33]. Negative attitudes also emerged in response to poor communication, as well as perceived blame and judgment, from healthcare professionals [31].

The neglect of carers’ needs and the lack of corresponding support were key sources of negative experiences during service engagement. Carers were often overlooked, dismissed, or inadequately supported by the mental health system and professionals throughout the care process, despite their considerable needs and efforts to be recognised [15,28,30,32,33]. For instance, “the families clearly did not perceive staff as being supportive…they were seldom, if ever, acknowledged when they visited their child…”, as cited in one review [15]. They required proactive support before, during, and after formal service engagement to understand the situations, clarify their role in it, navigate the challenges, and adapt both emotionally and practically [32,33].

In addition to the neglect of carers’ needs, carers consistently reported active exclusion from the treatment process and decision-making for service users, leading to frustration and helplessness. Carers wanted to help and “be treated as a resource” [33], to inform clinical decision making with their experiential knowledge about the service users and to provide consistent support to them [32,33]. However, they consistently described being excluded from key aspects of their loved ones’ care, including communication with staff, treatment decisions, and discharge planning [15,26,32–34]. The experience was captured in the following quote: “I wasn’t involved, I was an afterthought … no one told us anything, no one rang to keep us up to date with the plan of care” [15]. The exclusion often led to feelings of frustration, helplessness, and marginalisation [26,33].

While an unmet need for communication and information may be considered a component of neglect and exclusion, it was especially and consistently highlighted in carers’ narratives as a distinct barrier that undermined their experiences. Carers consistently reported an unmet need for proactive, timely, and relevant information regarding their loved ones’ care, their own caregiving role, and the services available [28,29,32]. They were unsure about how to help and seek help [28]. “Being left in the dark” was described to be scary and disappointing [29]. In addition, confidentiality policies acted as barriers, preventing carers from receiving the information they needed [32].

Meanwhile, carers noticed limitations in current mental health service models. Carers were concerned that staff in services had insufficient skills to provide care [18]. Some services, such as respite care, supported diverse service user groups simultaneously but were not tailored to their specific needs [18]. There was also a general lack of cultural diversity in existing services, with limited attention given to this issue [18]. These limitations reduced the services’ ability to meet the needs of carers and service users.

Although some carers expressed dissatisfaction with healthcare professionals during service engagement, others reported appreciation and emphasised some desired qualities. They expressed a desire for healthcare professionals to demonstrate affection, empathy, proactivity, and respect in their interactions [15]. They expressed appreciation for healthcare professionals’ “availability, competence, and positive attitudes like empathy, sensitivity, respectfulness, friendliness, and trustworthiness” [31]. Professionals with these helpful attributes were described as “some kind of saviour” [31].

Notably, carers were not a homogeneous group. Their views and experiences varied across individuals. While many carers desired inclusion, some expressed ambivalence about their involvement and support needs. For instance, some partners of women in perinatal mental health services depicted themselves as “struggling to identify their own needs, minimizing their own desires, prioritizing the woman, or expressing a reluctance to ask for or accept help or involvement” [28]. At the same time, carers reported divergent and inconsistent experiences with different treatment modalities [28,31]. Interventions that some carers viewed as appropriate and helpful were perceived by others as distressing, unhelpful, or even harmful [28,31]. These mixed experiences suggested that the suitability of treatment modalities may depend heavily on individual contexts, needs, and values.

#### Synthesised finding 6: Relational strain during the caregiving process.

Relational dynamics were a core aspect of carers’ experiences throughout the process of seeking help for service users and engaging with mental health services, and thus presented as a separate synthesised finding. Emotional and relational distance between carers and the individuals they supported was frequently reported in the process. Simultaneously, carers reported unsatisfactory interactions with healthcare professionals. These dual tensions contributed to a sense of isolation and burdens in the caring role.

Carers reported relational strain in their relationship with the service user. Distress arising from service users’ mental health difficulties, the power dynamics during the process, and social distancing, particularly in the context of service users’ detention, were key factors contributing to the strained relationships identified in the included reviews. Involuntary admission and hospitalisation could damage communication and relationships, leading to service users’ mistrust, rejection, and social distancing [15,26,32,33]. Some carers and service users blamed each other for the situation [15]. The power dynamic involved in the process contributed to the strained relationships. While many carers felt powerless, some experienced a sense of power in their roles through making decisions for service users and service users’ economic dependence [32]. The power unbalanced the relationship between carers and service users, causing emotional distance and distress [32]. Physical distancing caused by specialist services and practical difficulties to visit the service used also added to the weakened connection [15,26]. These relationship dynamics led to carers’ feelings of separation, loss, and distress [26,32].

Carers also experienced strain in their relationship with healthcare professionals, while they desired for partnership. They consistently expressed a desire for partnership with healthcare professionals and appreciated kind and respectful interactions [15,32]. However, their relationships with healthcare professionals were often unsatisfactory, marked by feelings of dismissal, invalidation, and a lack of compassion [32,33]. Tensions and divisions were reported in the relationship, as staff sometimes perceived carers as “threats, challenging, or nuisances” [15].

#### Findings not included in the final synthesis.

Six findings from the included reviews were not synthesised using the meta-aggregative approach due to their uniqueness or lack of supporting data across studies. These are presented separately to maintain transparency.

Four findings were identified but not integrated into the synthesised findings due to their uniqueness or lack of recurrence across reviews. These included the protective factors for siblings in early adulthood during the prodromal phase [19], practical barriers to involvement in services [28], parental observations of gradual positive changes in recovery [31], and family group conferencing regarded as useful [30]. These findings were acknowledged for their potential relevance but were excluded to maintain the robustness and coherence of the synthesis. Nonetheless, they suggested potentially important areas for future investigation. Two findings were excluded from the synthesis due to limited supporting evidence. One mentioned carers’ personal growth from the caring experiences [26], and the other addressed carers’ feeling of isolation [26]. Excluding findings with insufficient credibility ensured the validity and focus of the current synthesis.

### The confidence of synthesised findings

A narrative assessment indicated that confidence varied across the synthesised findings according to the methodological quality, quality and adequacy, breadth, and contextual diversity of the supporting evidence. Overall, confidence was greatest for findings supported by multiple high-quality reviews, numerous extracted findings, evidence from diverse mental health service settings, and primary studies conducted across a broad range of countries.

The methodological quality of included reviews was presented in **Table 1**, while the quality ratings of support from primary data of individual extracted findings were shown in **S1 Appendix** **(Sections C and D).**. Drawing on these assessments, the methodological quality of the contributing reviews and the extent to which the contributing extracted findings were supported by primary evidence were summarised for each category and presented in **S1 Appendix** **(E: Evidence and data underpinning the narrative confidence assessment Table E2)**. At the category level, almost all categories demonstrated high methodological quality of contributing reviews and unequivocal support from primary qualitative evidence. One category, “Limitations of current mental health service models”, was assessed as having moderate methodological quality and equivocal support, reflecting a comparatively weaker evidence base and warranting cautious interpretation. Across all synthesised findings, confidence was strengthened by the generally high methodological quality of the contributing reviews and by the fact that most contributing reviews explicitly supported their interpretations using primary qualitative data. Together, these characteristics suggest that the umbrella review findings are well grounded in the available qualitative evidence.

The overlap assessment indicated slight overlap among primary studies across contributing reviews (CCA = 1.6%), suggesting that the synthesised findings were largely informed by independent bodies of primary qualitative evidence [25]. Therefore, the potential for repeated inclusion of the same primary studies to disproportionately influence the synthesis was considered limited.

The service-context and geographical diversity of the primary studies, the number of contributing reviews and extracted findings to categories and synthesised findings, and the level of diversity supporting each synthesised finding were summarised and presented in **S1 Appendix**. Specifically, service-context and geographical diversity of the primary studies are presented in **S1 Appendix** **(Section E,Table E1)**, while the number of contributing extracted findings, reviews, and contexts for each category and synthesised finding are presented in **S1 Appendix** **(Section E, Table E2)**.

The included reviews synthesised primary qualitative studies from geographically diverse regions across Asia, Europe, North America, South America, Africa and Oceania. Only one Australia-focused review applied geographical restriction in primary study inclusion [18]. Despite breadth of included geographical settings in the primary reviews’ inclusion criteria, studies from high-income countries, particularly the United Kingdom, Australia, Canada, and the United States, were disproportionately numerous, with relatively limited representation from low- and middle-income countries. Consequently, the transferability of the findings to resource-constrained health systems should be interpreted with caution. Meanwhile, across the included reviews, carers’ experiences were examined in seven service contexts: inpatient care overall, compulsory admission specifically, and five specific clinical or service contexts — psychosis services, perinatal mental health services, eating disorder services, forensic mental health services, and respite care. The evidence was disproportionately derived from acute, inpatient, and high-intensity mental health services. Community-based, lower-intensity services were comparatively underrepresented. As a result, the synthesis primarily reflects carers’ experiences within specialist or crisis-oriented services, which may limit the applicability of some findings to low-intensity mental health settings.

Among the six synthesised findings, “Complex experiences of engaging with mental health services” received the strongest support, drawing on 29 extracted findings from 10 reviews representing all seven mental health service contexts. Within this synthesised finding, Dissatisfaction and distrust, Neglect of needs and lack of adequate support, and Exclusion from care and decision-making were each supported by five or more reviews and evidence from multiple service contexts, with contributing reviews generally providing clear links to primary qualitative findings. Similarly, “Emotional complexity in the caregiving journey” was supported by 24 extracted findings from 11 reviews across all seven service contexts, indicating substantial consistency in carers’ emotional experiences despite variation in clinical settings. “Multiple barriers for carers in accessing mental health support” was also supported by a broad evidence base, comprising 25 extracted findings from seven reviews across four mental health service contexts. The convergence of evidence across multiple reviews suggests that barriers to accessing support are common across different mental health services, although the predominance of evidence from high-income countries should be considered when interpreting the transferability of these findings. “Recognition and coping with their loved ones’ mental health difficulties”, “Persistent responsibilities and multiple roles in supporting service users”, and “Relational strain during the caregiving process” were supported by fewer reviews, extracted findings, and service contexts than the strongest themes, indicating somewhat lower confidence, with a narrower but internally consistent evidence base.

Confidence was comparatively lower for several categories supported by a more restricted evidence base. In particular, “Limitations of current mental health service models” was supported by only one integrative review on respite care services that included only Australian primary studies, with moderate methodological quality and equivocal support. Despite comprising three extracted findings, this category should be interpreted cautiously due to the limited breadth and strength of the supporting evidence. In addition, findings such as “Expectations of health professionals and services”, “Uncertainty and guarded hope about the future”, “Boosted help-seeking after reaching crisis point”, and “Variations in views and experiences” were each informed by only two or three reviews, reducing confidence in their transferability across different settings.

Taken together, the synthesised findings are well grounded in the available qualitative evidence, while recognising that confidence is lower for themes supported by fewer reviews or more limited contextual diversity. At the same time, the transferability of the findings to resource-constrained health systems and lower-intensity community services may be constrained by the predominance of high-income countries and acute mental health service settings.

## Discussion

### Main findings

This umbrella review synthesised qualitative evidence from 12 reviews to provide a comprehensive understanding of informal carers’ experiences of mental health services regarding supporting those they care for and getting support for their own wellbeing. Drawing on a meta-aggregative approach, six synthesised findings with strong confidence were developed, capturing carers’ emotional journey throughout the caregiving process, early recognition and management of their loved ones’ situations, barriers in accessing services, persistent caring for service users, experience and feedback regarding service engagement, and the relational strain in their caregiving roles.

While carers’ emotional distress and difficult experiences were prominent themes consistent with previous literature [9–13], some positive emotions, such as relief, gratitude, and hope, were reported when carers felt that their loved ones had received appropriate and high-quality care [15,26,28,32,33]. A few reviews also reported positive experiences, such as carers observed service users’ recovery-related changes [31] and carers’ personal growth through the caregiving journey [26], though each of these was only included in one review and lacked sufficient recurrence for synthesis. The presence of these positive experiences, though limited, suggests the potential helpfulness and constructive roles of mental health services, dimensions that are often underrepresented in the caregiving literature [35].

However, emotional distress and negative experiences still predominated in the current synthesis. Carers’ persistent emotional distress and difficult experiences reflect the heavy burdens and the multifaceted challenges throughout caregiving, which echo with the wider literature and are increasingly acknowledged by policymakers [4,17]. The findings highlight the urgent need for more specific, comprehensive, and sustained support for informal carers. The challenges and barriers identified in this review suggest potential areas for improvement within current mental health services, including service accessibility, better guidance for carers, greater carer involvement, and enhanced competence among healthcare professionals.

Although many of the experiences identified in this review have been reported previously, they have largely been presented within specific mental health conditions, service settings, or stages of service engagement. Rather than representing isolated encounters with services, this umbrella review provides a higher-level conceptual understanding of informal carers’ experiences throughout the mental health care trajectory. The synthesis demonstrates that carers’ experiences can be organised and understood around a dynamic caregiving journey, extending from recognising emerging mental health difficulties, through help-seeking, accessing and engaging with services, to long-term caregiving and ongoing uncertainty about the future. Across this journey, carers’ emotional experiences, caring responsibilities, and relationships with both service users and health professionals evolve continuously.

### Implications for practice

#### Enhancing services availability and accessibility.

The availability of mental health services for both carers and service users remain a significant concern and require improvement. While carers shouldered substantial burdens and stress in supporting the service users, their needs were often unaddressed by existing service structures [15, 33]. More services tailored to carers’ needs are needed. For example, respite care services can provide temporary relief [18]. Smaller embedded interventions targeted particularly at carers, such as family psychoeducational programs, are often experienced as helpful and could be promoted more widely for empowering families and benefiting carers’ well-being [36]. Interventions that help mediate the relationship strain between carers and service users may also be helpful to reduce carers’ potential sense of conflicts and social isolation when their loved ones are receiving services [15,26,30,32,33].

Also, as reflected in the current review, the demand for appropriate and continued services for service users with fewer access barriers has been high [19,26]. Access barriers have contributed to carers’ dissatisfaction, frustration, and exhaustion, as well as service users’ poorer long-term outcomes, since they often resulted in delayed access to professional services when early interventions were critical for improving outcomes across both common and serious mental issues [37,38]. Preventive care, community-based support, and low-threshold, low-intensity services have been identified as a feasible strategy to cope with these challenges, which could expand access, promote service inclusion, mitigate service users’ symptoms, and enhance support to carers [39–41]. However, the limitations of community care and pathways was also identified in the current review. This gap underscores the need for increased attention and promotion of accessible, community -based approaches. Encouragingly, innovative services of this type have emerged with demonstrated strengths. For instance, non-diagnostic, network-based *Open Dialogue* has facilitated transparent, collaborative decision-making and emphasises relational continuity, which improved engagement and support for both carers and service users [42]. Community-based residential crisis houses provided flexible, short-term support without formal psychiatric admission, reducing coercion and promoting inclusivity [43]. These approaches enhanced access, reduced carers’ burden, and established strong practical potential to improve outcomes in non-acute settings [42]. Both example services have been praised for their flexibility and inclusion, enhancing access and improving outcomes in non-acute settings [43], which underline the importance of promoting community- and low-intensity mental health services as a response to the current gaps in service provision.

#### Raising awareness and providing guidance.

While improved service availability and accessibility are essential, they are not sufficient on their own. Carers need awareness of these services and guidance on accessing and engaging with them effectively [18,26,28–30,32]. Upon recognising changes in their loved ones’ mental health, carers often attempted to interpret these shifts through their own knowledge and belief systems, which shaped subsequent coping strategies [19]. However, due to limited mental health knowledge, many delayed help-seeking until a ‘crisis point’, negatively impacting service users’ outcomes and carers’ own well-being [19,26,27,37,38]. This highlights the importance of public awareness campaigns to improve understanding of mental health difficulties, available supports, and pathways to care. Some initiatives have provided evidence of the positive impact of these campaigns, demonstrating how they can support both carers and service users. Example campaigns include *Every Mind Matters,* a UK public health campaign that enhanced mental health literacy, promoted self-care skills, and reduced stigma, with evidence showing it improved individuals’ help-seeking self-efficacy, empowering them to seek support more timely and confidently [44]. Similarly, *RU OK? Day*, an Australian initiative promoting peer support through structured conversations, has been associated with greater intentions, confidence, and actions to check in on others’ mental health, fostering broader social support and preventing severe mental health crisis [45]. The examples suggest the potential of establishing mental health public awareness initiatives to improve current services, which can benefit both carers’ and service users’ well-being by promoting coping strategies and fostering broader connectedness in their journey of accessing services and recovery.

During and after engagement with mental health services, many carers continued to experience a lack of guidance on navigating the system and sustaining their caregiving roles, which often made them feel ill-equipped and powerless to manage both their own well-being and the needs of their loved ones [28,29,32,33]. Services should provide more guidance and communication to carers, enhancing their engagement and empowering them to contribute effectively. This can benefit carers’ self-efficacy and emotional well-being, while also strengthening service delivery and service users’ outcomes. The community-based, family-led education program, *the Journey of Hope*, that strengthened families’ knowledge and coping competencies with proactive communication, is a good example [46]. When carers feel more empowered and informed, they sustain their caregiving responsibilities more effectively with improved self-efficacy, emotional resilience, and caregiving competencies, leading to improved outcomes [46]. The beneficial effects of offering more proactive communication and guidance are highlighted, as collaborative, family-centred approaches not only improve carers’ experiences but also strengthen the overall service outcomes by fostering more engaged, informed, and supportive caregivers.

In addition, carers observed that current service models often lack cultural sensitivity [18]. While mental health difficulties may involve biological components, they are largely experienced and interpreted through cultural frameworks [47,48]. The dominant knowledge of mental health, rooted primarily in Western ethnopsychiatry, has been critiqued for its limited cross-cultural validity [49]. Therefore, awareness initiatives should adopt culturally sensitive and inclusive approaches that consider diverse worldviews and lived experiences. Initiatives such as the Patient and Carer Race Equality Framework (PCREF) have been introduced to improve the inclusiveness and cultural competence of mental health services [50]. More specifically, the PCREF has guided healthcare providers in developing culturally competent care plans and advocated to train mental health professionals to interact with individuals from diverse backgrounds in a respectful and understanding way [50]. Such an approach may help to mitigate misunderstandings, reduce the risk of inappropriate treatment, and alleviate the additional social pressures that marginalised individuals potentially experience [50]. Culturally sensitive frameworks, such as the PCREF, could be more widely promoted and adopted to address the cultural insensitivity concerns raised by carers. These frameworks may help more effectively support both carers and service users from diverse backgrounds, fostering social inclusion and promoting equity in mental health services.

#### Promoting carers’ involvement.

Within mental health services, improving the recognition of carers’ needs and enhancing their inclusion is crucial. Carers possessed profound experiential knowledge of the service users [32,34] and demonstrated sustained motivation, sense of responsibility, and willingness to persistently participate in care and support [30,32,34]. Excluding them not only led to marginalisation, mistrust, and a sense of powerlessness, but also wasted valuable resources that could support service users’ recovery [26,32–34].

Reconsidering and strengthening carers’ roles within services is therefore essential. The UK’s *Triangle of Care* model is a good example of frameworks that address these considerations [51]. It promotes collaboration between service users, professionals, and carers, encouraging routine information-sharing and carers’ meaningful involvement in care planning [51]. The model has shown promise in enhancing relational, emotional, and clinical outcomes in both carers and service users [51,52]. Interventions that facilitate carers’ involvement in treatment decisions, discharge planning, and aftercare are also highly valuable [53]. For instance, family group conferencing and a caregiver-involved discharge planning process have helped reduce caregiver burden and improved carers’ health status, demonstrating positive impacts of structured carer inclusion [30,54]. Embedding carers within the treatment process, cooperative and inclusive models, such as the examples discussed above, show significant potential in addressing the challenges of carer inclusion identified in this review. These models foster greater engagement, reduce isolation, and enhance the well-being of both carers and service users by ensuring a more holistic, integrated, and inclusive approach to care. Such frameworks should be actively promoted to strengthen support for carers and improve overall outcomes.

Policies such as confidentiality, while essential for protecting service users’ rights, can sometimes hinder carer involvement [32]. Negotiation and flexibility are needed to navigate these tensions. Frameworks have emerged to address this issue. For example, when service users withhold consent, a best practice framework recommends a context-dependent distinction between general information (e.g., illness education) that can be shared without consent and service users’ personal information that cannot, allowing carers to receive support without breaching confidentiality [55]. Flexible, situation-dependent frameworks like this can help address policy limitations, balancing the need for carer involvement with the protection of service users’ privacy and ultimately enhancing the quality of care and support provided to both carers and service users.

#### Improving healthcare professionals’ competence.

Last but not least, improving the professional competence of healthcare professionals is essential. Professionals need not only clinical knowledge and skills to make accurate diagnoses and suggest appropriate treatment options, but also the ability to recognise, engage, and collaborate with carers effectively [15,31–33]. Beyond clinical knowledge, healthcare professionals’ attitudes and interpersonal behaviours are a vital part of their clinical expertise and play a crucial role in shaping carers’ and service users’ experiences. Some staff displayed dismissive, stigmatising, or blaming attitudes, leading to carers’ distrust, distress, and reduced engagement [31]. Conversely, carers appreciated professionals who demonstrated kindness, respect, compassion, and a genuine concern for the needs of both service users and their families [15,31]. These attitudes should be actively fostered within healthcare professionals.

#### Strengthening system-level approaches to carer support.

Insufficient information and guidance, fragmented communication, limited involvement in decision-making, inadequate inclusion and respect, and insufficient support directed towards carers themselves were described relatively consistently by carers. The recurring nature of these challenges suggests that many of the challenges experienced by carers are structural rather than isolated problems arising within individual services or geographical settings. Also, support for carers should not be conceptualised as a single intervention delivered at one point in the care pathway. Instead, carers require ongoing support that adapts to their changing informational, emotional, and practical needs as their interactions with mental health services evolve.

Recent international and national initiatives increasingly emphasise recognising, involving, and supporting informal carers of people with mental health difficulties. For instance, the World Health Organisation Comprehensive Mental Health Action Plan advocates involving families and carers in treatment and providing information, care and support for them [56]. Comparable commitments have been reflected in national mental health policies, such as the UK Government’s Unpaid Carers Action Plan: Recognise, Refer, Reach [57] and New Zealand’s Mental Health and Addiction Service Development Plan [58], which have increasingly recognised informal carers as essential partners in care whose contributions and knowledge should be actively valued and integrated, while also acknowledging their own support needs. However, current findings suggest that the translation of these principles into routine practice remains inconsistent and challenging. Structural limitations identified in the current synthesis are unlikely to be fully addressed by fragmented, service-level improvements. Instead, improvements may require coordinated organisational and system-level approaches that ensure carer-inclusive practice is implemented consistently across different services and stages of the mental health care pathway. Future implementation efforts may benefit from embedding carer-inclusive practice within routine organisational processes and care pathways. This may include establishing consistent standards for information sharing and carer involvement, providing workforce training on collaborative working with carers, and ensuring continuity of support across transitions between services. Such system-level approaches may better address the recurring barriers identified throughout the caregiving journey than isolated interventions delivered within individual services.

#### Context-specific service implications.

Although the findings of this umbrella review primarily represent overarching experiences shared across different mental health service contexts, additional consideration of context-specific patterns identified several areas where tailored service responses may be particularly relevant. To explore this, where multiple reviews focused on the same service context, whether particular synthesised findings were consistently identified across reviews within that context was examined.

In three reviews regarding compulsory admission contexts, carers consistently reported dissatisfaction and distrust towards services, unmet needs and insufficient support, and strained relationships with service users. Services should prioritise approaches that promote trust, collaboration, and shared understanding. This may include providing clear and timely psychoeducation and explanations about mental health difficulties and compulsory interventions, and maintaining transparent communication. Also, carers may require specific emotional and practical support to cope with the distress associated with compulsory admission and relationship tension with the person they support. While balancing service-user autonomy and confidentiality with carers’ need for support and information remains essential, flexible approaches to information sharing, such as distinguishing between general information that can be provided without consent and confidential personal information as mentioned earlier, may help [55].

In two reviews about inpatient care, carers consistently described mixed emotional responses following service users’ admission, with relief that their loved ones were receiving care often coexisting with uncertainty, guilt, loneliness, and ongoing distress. These complicated and nuanced feelings highlight carers as individuals with their own support needs. Alongside involving carers in treatment where appropriate, services could provide clear explanations of mental health conditions and treatment processes, combined with empathetic acknowledgement and support for carers’ emotional experiences, including guilt, uncertainty, and feelings of isolation. Offering emotional support and signposting carers to peer support or other relevant services during admission may help carers adapt to changing roles, improve competence and confidence in daily care-giving, and better prepare for the transition to community care following discharge.

In three reviews of psychosis-related services, the consistent identification of high distress during the help-seeking process, crisis-driven service engagement, and personal and relational barriers to accessing support highlights the importance of proactive carer-facing support before psychosis situations escalate. Although optimal approaches are not yet clearly established, proactive early detection strategies and targeted health education from early psychosis services are warranted to improve service users’ care pathways and outcomes, and make it easier for carers to recognise psychosis and seek help [59]. Once people with psychosis have reached services, community mental health and early intervention services could consider early, proactive engagement with carers, including providing accessible psychoeducation about emerging symptoms, available pathways to support, and strategies for managing relational difficulties. Such approaches are consistent with family intervention models in psychosis, which aim to enhance carers’ understanding, coping capacity, and involvement in recovery-oriented care [60]. Earlier support may help reduce reliance on crisis points as the primary trigger for accessing mental health services and lessen the influence of personal and relational barriers on timely help-seeking.

### Implications for research

As mentioned earlier, emotional distress and negative experiences predominated in the current synthesis. Methodological and structural biases within the included reviews may have contributed to the skew. Many of the existing qualitative studies in the area were guided by a longstanding deficit-focused approach, where studies are primarily motivated by concerns related to distress, system inadequacies, or service failures [35,61–63]. For instance, 8 out of 12 included reviews focused on involuntary treatment, hospitalisation, and psychosis-related services, which are high-intensity settings with salient stress [32]. This reflects a broader pattern in the literature, where carers’ experiences in acute or coercive contexts are more frequently studied, entrenching problem-oriented perspectives. Potential overlaps in primary studies across the included reviews may also inflate the bias [24]. The absence of reviews that explicitly focus on community services or low-intensity care reflects a notable gap in perspective. Furthermore, potential underrepresentation of carers’ positive experiences may be amplified in umbrella reviews since such accounts may be deemed too infrequent or nuanced to warrant synthesis inclusion, leading to progressive downplaying at each stage of synthesis [24,64]. Future research that deliberately attends to carers’ strengths and positive experiences is needed to provide a more balanced and holistic understanding of their caregiving journey. At the same time, more studies on community-based or low-intensity mental health services are also increasingly called for. Such research would complement existing knowledge from acute service settings, informing the development of more comprehensive, cooperative, tailored, and accessible interventions that better include both carers and service users, effectively reduce access barriers, and improve outcomes.

In addition, limited demographic and socio-cultural details were reported across reviews, revealing a gap in the current evidence base. When some demographic details were available, most primary studies were conducted in high-income countries with predominantly adult female participants, limiting the generalisability of findings. The gap underscores the need for research that diversifies participant demographics and concerns underrepresented groups, such as ethnic minority carers and young male carers. Such research would deepen understanding of how cultural values, gender norms, and social resources shape carers’ engagement with mental health services and their own wellbeing and provide insights into how services can be improved to best meet the needs of different groups [20].

In addition, only one included review on respite care explicitly examined interventions supporting carers’ own mental health [18]. Consequently, this synthesis provided limited evidence on services that directly promote carers’ wellbeing, highlighting the demand for research on carers’ broader needs and targeted interventions. Given the need to enhance current services, future research should also prioritise the development and evaluation of service models designed to support carers.

### Limitations

This review has several limitations. First, although focusing on carers’ experiences of mental health services, many primary reviews also included carers’ broader experiences of supporting their loved ones. These findings were included. However, wider literature on carers’ general experiences was not comprehensively captured, potentially omitting valuable insights.

Second, to avoid missing potentially relevant evidence, any form of literature review which involved searching at least two bibliographic databases with transparent search strategies was included in this umbrella review. It is acknowledged that including reviews with different methodological approaches may introduce variability in methodological rigour and reporting quality. Thus, to examine the potential influence of reviews with different methodological approaches on the robustness of our synthesised findings, an additional assessment of the contribution of findings from the narrative review by Rodell and Parry [26] and the integrative review by Almoaber et al. [18] was done. All findings informed by Rodell and Parry’s narrative review [26] were also supported by one or more systematic reviews. Therefore, excluding this review would not have materially altered the synthesised findings or the overall conclusions. For Almoaber et al. [18], most contributions were similarly supported by systematic reviews. The only exception was the category “Limitations of current mental health service models” within the fifth synthesised finding “Complex experiences of engaging with mental health services”, which was derived solely from this integrative review. Excluding this review would have reduced the comprehensiveness of the synthesis by removing insights into carers’ experiences of structural limitations in mental health service provision, including concerns regarding staff expertise, the suitability of respite services, and the cultural responsiveness of care. Moreover, the broader synthesised finding remained supported by multiple systematic reviews, suggesting that the inclusion of this review did not materially affect the overall conclusions of the umbrella review.

Third, during the study selection process, only 10% of the retrieved records were independently double-screened at the title and abstract stage due to resource constraints. This approach is consistent with Cochrane guidance, which states that while duplicate screening at the title and abstract stage is desirable, it is not essential [22]. The high level of agreement achieved during the 10% double-screening suggests a slim likelihood of missing relevant records. Nevertheless, it is acknowledged that duplicate screening of all records would have been desirable. Future reviews should consider implementing duplicate screening throughout the entire study selection process where feasible to further minimise the risk of selection bias.

Fourth, while providing a high-level, overarching synthesis, the umbrella review and meta-aggregative approach has some inherent methodological constraints. Both the cumulative synthesis process of an umbrella review and the meta-aggregative method, which prioritises summarising findings over comparative or interpretive analysis, may oversimplify complex experiences, reduce contextual richness, and omit nuanced or minority perspectives, thereby biasing results toward dominant themes [64,65]. The method has limited ability to inform context-specific recommendations. Although contextual patterns were explored where evidence was available, findings regarding specific service settings should be interpreted cautiously. At the same time, the prevailing deficit focus across the included studies, combined with the umbrella review’s inclination to highlight dominant narratives, may have inadvertently obscured positive experiences or developments occurring within mental health services. In addition, as umbrella reviews are built upon systematic reviews, which in turn synthesise primary studies, there is an inevitable temporal lag that may obscure recent developments in service provision [64]. The meta-aggregative approach may also lack interpretive depth and flexibility, as it relies on the synthesis of existing reviews [65,66]. Together, these factors should be considered when understanding and interpreting the synthesised findings.

Fifth, demographic and contextual information were limited in the included reviews, making it difficult to understand how factors, such as carers’ age, gender, ethnicity, or socio-cultural backgrounds, may have influenced their experiences of accessing and engaging with professional mental health services. Also, as for context, the included reviews disproportionately focused on acute and hospital-based contexts, with few findings reflecting carers’ experiences in community or low-intensity services and did not differentiate findings by context. As a result, the synthesis mainly reflects high-intensity or coercive settings, limiting generalisability.

Finally, there is currently no established framework for assessing the certainty or confidence of findings in qualitative umbrella reviews. In accordance with the pre-registered protocol, the initial plan was to adapt the ConQual approach to assess confidence in the synthesised findings. ConQual was developed for qualitative systematic reviews synthesising primary qualitative studies and assesses confidence based on the dependability and credibility of the contributing primary evidence [20]. However, it does not explicitly account for methodological considerations that are particularly relevant to umbrella reviews, such as the breadth of evidence across multiple reviews, variation in study contexts, and the transferability of findings across settings. Consequently, a narrative assessment of the robustness of the synthesised findings was undertaken to facilitate interpretation. Nevertheless, this approach has not been formally validated for qualitative umbrella reviews. Further methodological research is needed to develop and evaluate certainty assessment frameworks that are specifically designed for qualitative umbrella reviews.

### Conclusion

This umbrella review synthesised qualitative review-level evidence on informal carers’ experiences of accessing and engaging with mental health services. Drawing on twelve qualitative reviews and using a meta-aggregative approach, the synthesis identified six key findings that reflected carers’ emotional complexity, efforts to cope and engage with services, systematic challenges and limitations of services they experienced, and relational tensions embedded in the caregiving experience. The findings highlight an urgent need to reform mental health services in ways that actively involve, inform, and support informal carers before, during, and after service users’ formal service engagement. Improving service accessibility, raising public awareness and providing guidance, facilitating carer involvement, and enhancing professionals’ competencies are critical priorities. This review provides a foundation for future research, service innovation, and policy development that centres carers as essential partners in mental health systems.

## Supporting information

S1 File S1 Appendix. (DOCX)
