# Supplementary material for: Informal carers’ experiences of mental health services regarding supporting those they care for and getting support for their own wellbeing: A systematic umbrella review of qualitative evidence
Source: PLOS Ment Health. 2026 Oct 15;3(10):e0000740. doi: 10.1371/journal.pmen.0000740 (PMC13671576; doi:10.1371/journal.pmen.0000740)
Supplement: Supplementary file 1 [file pmen.0000740.s001.docx]

**A. Search strategy: Complete query strings.**

**APA PsycInfo**

1 caregivers/ or caregiver attitudes/ or caregiver burden/ or caregiving/ or caring behaviors/

2 family/ or family members/

3 partners/ or significant others/

4 (carer* or caregiver* or famil* or parent* or mother* or father* or spouse* or wife or wives or husband* or offspring* or daughter* or son* or child* or friend* or neighbo?r* or partner* or sibling* or brother* or sister* or “significant other*”).ti,ab,id.

5 1 or 2 or 3 or 4

6 exp mental disorders/ or exp mental health services/

7 “mental health service*”.ti,ab,id.

8 ((mental or psychology* or psychiatr*) adj3 (problem* or disord* or issue* or illness* or condition* or difficult* or challeng*)).ti,ab,id.

9 (depress* or anxi* or bipolar or phobi* or “mood disord*” or “affective disord*” or “obsessive-compulsive disord*” or “OCD” or “self-harm” or “suicidal ideation” or “suicide attempt*” or “emotional distress” or psychosis or “psychotic disord*” or schizophreni* or “personality disord*” or “complex emotion disord*” or “eating disord*” or anorex* or bulimi* or “substance use disord*” or “trauma-related disord*” or PTSD or “post-traumatic stress disord*” or “conduct disord*”).ti,ab,id.

10 6 or 7 or 8 or 9

11 (metasynthesis or “systematic review”).md. or “systematic review”/ or “systematic reviews as topic”.mh.

12 ((comprehensive or comprehensively) adj (analysis or review or reviewed)).ti,ab,id.

13 ((literature adj (search or searches)) and (database or databases or cinahl or cochrane or embase or psycinfo or pubmed or medline or scopus or (web adj1 science))).ti,ab,id.

14 ((electronic adj (database or databases)) and (eligibility or excluded or exclusion or included or inclusion)).ti,ab,id.

15 ((exploratory adj review) or (framework adj synthesis)).ti,ab,id.

16 ((meta adj (epidemiological or ethnographic or ethnography or interpretation or narrative or review or study or synthesis or summary or theory)) or meta?ethnographic or meta?ethnography or meta?synthesis).ti,ab,id.

17 (narrative adj1 synthesis).ti,ab,id.

18 (“PRISMA” adj4 (guideline or guidelines or preferred or reporting or requirements)).ti,ab,id.

19 ((systematic adj2 literature) or (systematic adj2 (Embase or Medline or PsycInfo or PubMed)) or (systematic adj2 review) or ((systematical or systematically) adj2 (review or reviewed)) or (systematically adj identified) or (systematized adj review)).ti,ab,id.

20 11 or 12 or 13 or 14 or 15 or 16 or 17 or 18 or 19

21 exp qualitative methods/

22 (qualitative or experience* or interview* or mixed-method or “mixed method” or focus group* or thematic analysis).ti.

23 ((qualitative adj (evidence or research)) and ((evidence or research) adj synthesis)).ti,ab,id.

24 21 or 22 or 23

25 5 and 10 and 20 and 24

**Ovid MEDLINE(R) ALL**

1 Caregivers/px [Psychology]

2 Family/px [Psychology]

3 Friends/px [Psychology]

4 Caregiver Burden/

5 (carer* or caregiver* or famil* or parent* or mother* or father* or spouse* or wife or wives or husband* or offspring* or daughter* or son* or child* or friend* or neighbo?r* or partner* or sibling* or brother* or sister* or “significant other*”).ti,ab,kf.

6 1 or 2 or 3 or 4 or 5

7 Mental Health/

8 exp Mental Health Services/

9 exp Mental Disorders/px [Psychology]

10 “mental health service*”.ti,ab,kf.

11 ((mental or psychology* or psychiatr*) adj3 (problem* or disord* or issue* or illness* or condition* or difficult* or challeng*)).ti,ab,kf.

12 (depress* or anxi* or bipolar or phobi* or “mood disord*” or “affective disord*” or “obsessive-compulsive disord*” or “OCD” or “self-harm” or “suicidal ideation” or “suicide attempt*” or “emotional distress” or psychosis or “psychotic disord*” or schizophreni* or “personality disord*” or “complex emotion disord*” or “eating disord*” or anorex* or bulimi* or “substance use disord*” or “trauma-related disord*” or PTSD or “post-traumatic stress disord*” or “conduct disord*”).ti,ab,kf.

13 7 or 8 or 9 or 10 or 11 or 12

14 “systematic review”/

15 Systematic Reviews as Topic/

16 ((comprehensive or comprehensively) adj (analysis or review or reviewed)).ti,ab,kf.

17 ((literature adj (search or searches)) and (database or databases or cinahl or cochrane or embase or psycinfo or pubmed or medline or scopus or (web adj1 science))).ti,ab,kf.

18 ((electronic adj (database or databases)) and (eligibility or excluded or exclusion or included or inclusion)).ti,ab,kf.

19 ((exploratory adj review) or (framework adj synthesis)).ti,ab,kf.

20 ((meta adj (epidemiological or ethnographic or ethnography or interpretation or narrative or review or study or synthesis or summary or theory)) or meta?ethnographic or meta?ethnography or meta?synthesis).ti,ab,kf.

21 (narrative adj1 synthesis).ti,ab,kf.

22 (“PRISMA” adj4 (guideline or guidelines or preferred or reporting or requirements)).ti,ab,kf.

23 ((systematic adj2 literature) or (systematic adj2 (Embase or Medline or PsycInfo or PubMed)) or (systematic adj2 review) or ((systematical or systematically) adj2 (review or reviewed)) or (systematically adj identified) or (systematized adj review)).ti,ab,kf.

24 14 or 15 or 16 or 17 or 18 or 19 or 20 or 21 or 22 or 23

25 qualitative research/

26 (qualitative or experience* or interview* or mixed-method or “mixed method” or focus group* or thematic analysis).ti.

27 ((qualitative adj (evidence or research)) and ((evidence or research) adj synthesis)).ti,ab,kf.

28 25 or 26 or 27 29 6 and 13 and 24 and 28

**CINAHL Plus**

S1 (MH “Caregivers”) OR (MH “Caregiver Burden”) OR (MH “Caregiver Attitudes”) OR (MH “Caregiver Support”)

S2 (MH “Family”) OR (MH “Children of Impaired Parents”) OR (MH “Daughters”) OR (MH “Children of Persons with Alcoholism”) OR (MH “Only Child”) OR (MH “Parents”) OR (MH “Fathers”) OR (MH “Mothers”) OR (MH “Parents of Children with Disabilities”) OR (MH “Parents of LGBTQ+ Persons”) OR (MH “Siblings”) OR (MH “Sons”) OR (MH “Spouses”) OR (MH “Grandchildren”) OR (MH “Grandparents”)

S3 (MH “Significant Other”)

S4 TI (carer* or caregiver* or famil* or parent* or mother* or father* or spouse* or wife or wives or husband* or offspring* or daughter* or son* or child* or friend* or neighbo#r* or partner* or sibling* or brother* or sister* or “significant other*”) OR AB (carer* or caregiver* or famil* or parent* or mother* or father* or spouse* or wife or wives or husband* or offspring* or daughter* or son* or child* or friend* or neighbo#r* or partner* or sibling* or brother* or sister* or “significant other*”)

S5 S1 OR S2 OR S3 OR S4

S6 (MH “Mental Health”) OR (MH “Mental Health Services”)

S7 (MH “Mental Disorders+”) OR (MH “Persons with Mental Disorders”)

S8 TI (“mental health service*”) OR AB (“mental health service*”)

S9 TI ((mental or psychology* or psychiatr*) N3 (problem* or disord* or issue* or illness* or condition* or difficult* or challeng*)) OR AB ((mental or psychology* or psychiatr*) N3 (problem* or disord* or issue* or illness* or condition* or difficult* or challeng*))

S10 TI (depress* or anxi* or bipolar or phobi* or “mood disord*” or “affective disord*” or “obsessive-compulsive disord*” or “OCD” or “self-harm” or “suicidal ideation” or “suicide attempt*” or “emotional distress” or psychosis or “psychotic disord*” or schizophreni* or “personality disord*” or “complex emotion disord*” or “eating disord*” or anorex* or bulimi* or “substance use disord*” or “trauma-related disord*” or PTSD or “post-traumatic stress disord*” or “conduct disord*”) OR AB (depress* or anxi* or bipolar or phobi* or “mood disord*” or “affective disord*” or “obsessive-compulsive disord*” or “OCD” or “self-harm” or “suicidal ideation” or “suicide attempt*” or “emotional distress” or psychosis or “psychotic disord*” or schizophreni* or “personality disord*” or “complex emotion disord*” or “eating disord*” or anorex* or bulimi* or “substance use disord*” or “trauma-related disord*” or PTSD or “post-traumatic stress disord*” or “conduct disord*”)

S11 S6 OR S7 OR S8 OR S9 OR S10

S12 (MH “Systematic Review”)

S13 PT (metasynthesis OR “systematic review”)

S14 TI ((comprehensive OR comprehensively) N (analysis OR review OR reviewed)) OR AB ((comprehensive OR comprehensively) N (analysis OR review OR reviewed))

S15 TI ((literature N (search or searches)) and (database or databases or cinahl or cochrane or embase or psycinfo or pubmed or medline or scopus or (web N1 science))) OR ((literature N (search or searches)) and (database or databases or cinahl or cochrane or embase or psycinfo or pubmed or medline or scopus or (web N1 science)))

S16 TI ((electronic N (database or databases)) and (eligibility or excluded or exclusion or included or inclusion)) OR AB ((electronic N (database or databases)) and (eligibility or excluded or exclusion or included or inclusion))

S17 TI ((exploratory N review) or (framework N synthesis)) OR AB ((exploratory N review) or (framework N synthesis))

S18 TI ((meta N (epidemiological or ethnographic or ethnography or interpretation or narrative or review or study or synthesis or summary or theory)) or meta#ethnographic or meta#ethnography or meta#synthesis) OR AB ((meta N (epidemiological or ethnographic or ethnography or interpretation or narrative or review or study or synthesis or summary or theory)) or meta#ethnographic or meta#ethnography or meta#synthesis)

S19 TI (narrative N1 synthesis) OR AB (narrative N1 synthesis)

S20 TI (“PRISMA” N4 (guideline or guidelines or preferred or reporting or requirements)) OR AB (“PRISMA” N4 (guideline or guidelines or preferred or reporting or requirements))

S21 TI ((systematic N2 literature) or (systematic N2 (Embase or Medline or PsycInfo or PubMed)) or (systematic N2 review) or ((systematical or systematically) N2 (review or reviewed)) or (systematically N identified) or (systematized N review)) OR AB ((systematic N2 literature) or (systematic N2 (Embase or Medline or PsycInfo or PubMed)) or (systematic N2 review) or ((systematical or systematically) N2 (review or reviewed)) or (systematically N identified) or (systematized N review))

S22 S12 OR S13 OR S14 OR S15 OR S16 OR S17 OR S18 OR S19 OR S20 OR S21

S23 (MH “Qualitative Studies”) OR (MH “Meta Synthesis”) OR (MH “Thematic Analysis”)

S24 TI (qualitative or experience* or interview* or mixed-method or “mixed method” or focus group* or thematic analysis)

S25 TI ((qualitative N (evidence or research)) and ((evidence or research) N synthesis)) OR AB ((qualitative N (evidence or research)) and ((evidence or research) N synthesis))

S26 S23 OR S24 OR S25

S27 S5 AND S11 AND S22 AND S26

**Epistemonikos**

(title:(carer* OR caregiver* OR famil* OR parent* OR mother* OR father* OR spouse* OR wife OR wives OR husband* OR offspring* OR daughter* OR son* OR child* OR friend* OR neighbor* OR neighbour* OR partner* OR sibling* OR brother* OR sister* OR “significant other”) OR abstract:(carer* OR caregiver* OR famil* OR parent* OR mother* OR father* OR spouse* OR wife OR wives OR husband* OR offspring* OR daughter* OR son* OR child* OR friend* OR neighbor* OR neighbour* OR partner* OR sibling* OR brother* OR sister* OR “significant other”))

AND

(title:(“mental health service” OR “mental health services” OR “mental problem” OR “mental disorder” OR “mental issue” OR “mental illness” OR “mental condition” OR “mental difficulties” OR “mental challenges” OR “psychological problem” OR “psychological disorder” OR “psychological issue” OR “psychological illness” OR “psychological condition” OR “psychological difficulties” OR “psychological challenges” OR “psychiatric problem” OR “psychiatric disorder” OR “psychiatric issue” OR “psychiatric illness” OR “psychiatric condition” OR “psychiatric difficulties” OR “psychiatric challenges” OR depress* OR anxi* OR bipolar OR phobi* OR “mood disorder” OR “affective disorder” OR “obsessive-compulsive disorder” OR “OCD” OR “self-harm” OR “suicidal ideation” OR “suicide attempt” OR “emotional distress” OR psychosis OR “psychotic disorder” OR schizophreni* OR “personality disorder” OR “complex emotion disorder” OR “eating disorder” OR anorex* OR bulimi* OR “substance use disorder” OR “trauma-related disorder” OR PTSD OR “post-traumatic stress disorder” OR “conduct disorder”) OR abstract:(“mental health service” OR “mental health services” OR “mental problem” OR “mental disorder” OR “mental issue” OR “mental illness” OR “mental condition” OR “mental difficulties” OR “mental challenges” OR “psychological problem” OR “psychological disorder” OR “psychological issue” OR “psychological illness” OR “psychological condition” OR “psychological difficulties” OR “psychological challenges” OR “psychiatric problem” OR “psychiatric disorder” OR “psychiatric issue” OR “psychiatric illness” OR “psychiatric condition” OR “psychiatric difficulties” OR “psychiatric challenges” OR depress* OR anxi* OR bipolar OR phobi* OR “mood disorder” OR “affective disorder” OR “obsessive-compulsive disorder” OR “OCD” OR “self-harm” OR “suicidal ideation” OR “suicide attempt” OR “emotional distress” OR psychosis OR “psychotic disorder” OR schizophreni* OR “personality disorder” OR “complex emotion disorder” OR “eating disorder” OR anorex* OR bulimi* OR “substance use disorder” OR “trauma-related disorder” OR PTSD OR “post-traumatic stress disorder” OR “conduct disorder”))

AND

(title:(qualitative))

Filter: Publication type: systematic review

**Cochrane library**

#1 MeSH descriptor: [Caregivers] this term only

#2 MeSH descriptor: [Caregiver Burden] this term only

#3 MeSH descriptor: [Family] this term only

#4 MeSH descriptor: [Friends] this term only

#5 (carer* or caregiver* or famil* or parent* or mother* or father* or spouse* or wife or wives or husband* or offspring* or daughter* or son* or child* or friend* or neighbo?r* or partner* or sibling* or brother* or sister* or (significant NEXT other*)):ti,ab,kw

#6 #1 OR #2 OR #3 OR #4 OR #5

#7 MeSH descriptor: [Mental Health Services] this term only

#8 MeSH descriptor: [Mental Health] this term only

#9 ((mental NEXT health NEXT service*)):ti,ab,kw

#10 ((mental OR psychology* OR psychiatr*) NEAR/3 (problem* OR disord* OR issue* OR illness* OR condition* OR difficult* OR challeng*)):ti,ab,kw

#11 (depress* OR anxi* OR bipolar OR phobi* OR (mood NEXT disord*) OR (affective NEXT disord*) OR (obsessive-compulsive NEXT disord*) OR OCD OR “self-harm” OR “suicidal ideation” OR (suicide NEXT attempt*) OR “emotional distress” OR psychosis OR (psychotic NEXT disord*) OR schizophreni* OR (personality NEXT disord*) OR (complex NEXT emotion NEXT disord*) OR (eating NEXT disord*) OR anorex* OR bulimi* OR (substance NEXT use NEXT disord*) OR (trauma-related NEXT disord*) OR PTSD OR (post-traumatic NEXT stress NEXT disord*) OR (conduct NEXT disord*)):ti,ab,kw

#12 #7 OR #8 OR #9 OR #10 OR #11

#13 MeSH descriptor: [Systematic Review] this term only

#14 MeSH descriptor: [Systematic Reviews as Topic] this term only

#15 ((comprehensive or comprehensively) NEXT (analysis or review or reviewed)):ti,ab,kw

#16 ((literature NEAR/1 (search OR searches)) AND (database OR databases OR cinahl OR cochrane OR embase OR psycinfo OR pubmed OR medline OR scopus OR (web NEAR/1 science))):ti,ab,kw

#17 ((electronic NEAR/1 (database OR databases)) AND (eligibility OR excluded OR exclusion OR included OR inclusion)):ti,ab,kw

#18 ((exploratory NEAR/1 review) OR (framework NEAR/1 synthesis)):ti,ab,kw

#19 ((meta NEAR/1 (epidemiological OR ethnographic OR ethnography OR interpretation OR narrative OR review OR study OR synthesis OR summary OR theory)) OR metaethnographic OR metaethnography OR metasynthesis):ti,ab,kw

#20 (narrative NEAR/1 synthesis):ti,ab,kw

#21 ((PRISMA) NEAR/4 (guideline or guidelines or preferred or reporting or requirements)):ti,ab,kw

#22 ((systematic NEAR/2 literature) OR (systematic NEAR/2 (Embase OR Medline OR PsycInfo OR PubMed)) OR (systematic NEAR/2 review) OR ((systematical OR systematically) NEAR/2 (review OR reviewed)) OR (systematically NEAR/2 identified) OR (systematized NEAR/2 review)):ti,ab,kw

#23 #13 OR #14 OR #15 OR #16 OR #17 OR #18 OR #19 OR #20 OR #21 OR #22 13911

#24 MeSH descriptor: [Qualitative Research] this term only

#25 (qualitative or experience* or interview* or mixed-method or (mixed NEXT method*) or focus group* or thematic analysis):ti

#26 ((qualitative NEAR/1 (evidence OR research)) AND ((evidence OR research) NEAR/1 synthesis)):ti,ab,kw

#27 #24 OR #25 OR #26

#28 #6 AND #12 AND #23 AND #27

**B. Records excluded at the full-text stage.**

96 reviews were assessed for eligibility at the full-text stage, and 84 were excluded. 27 reviews were excluded because the phenomenon of interest was not experiences regarding mental health services; 9 reviews were excluded because of ineligible study designs, as findings from qualitative studies or themes relating to informal carers’ experiences could not be distinguished; 21 reviews were excluded because of an ineligible population (i.e. formal carers, informal carers of people without mental health difficulties, or carers of individuals with neurocognitive or neurodevelopmental disorders); 27 reviews were excluded because they examined the experiences of informal carers of individuals with mental health difficulties in general rather than focusing on experiences of accessing or engaging with mental health services.

Below is a list of the 84 reviews excluded at the full-text stage, along with the reasons for their exclusion.

**Wrong phenomenon of interest (n = 27):**

1. Atkinson J, Smith V, Carroll M, Sheaf G, Higgins A. Perspectives of partners of mothers who experience mental distress in the postnatal period: A systematic review and qualitative evidence synthesis. Midwifery. 2021;93:102868.
2. Boone K, Geerts P-J, Van de Velde N, Verhaeghe S, Lemmens GMD. Relatives' Knowledge, Attitudes, and Experiences Toward Electroconvulsive Therapy: A Systematic Review. The journal of ECT. 2024.
3. Buus N, Petersen A, McPherson S, Meadows G, Brand G, Ong B, et al. The relatives of people with depression: A systematic review and methodological critique of qualitative studies. Family Process. 2023:No-Specified.
4. Clerc E, Mesquida L, Raynaud J-P, Revet A, American Psychiatric Association BLMSMMMCMBUBB-LSB-LBHRBLGD, et al., et al. Therapeutic groups for parents of patients with anorexia nervosa: A systematic review of subjective experiences and state of current practices in France. Annales Medico-Psychologiques. 2023;181(6):509-20.
5. Dam K, Hall EOC, Aldridge J AAJAAJBSAALABHKBSIAAHTAM. Navigating in an unpredictable daily life: A metasynthesis on children's experiences living with a parent with severe mental illness. Scandinavian Journal of Caring Sciences. 2016;30(3):442-57.
6. Huddlestone L, Shoesmith E, Pervin J, Lorencatto F, Watson J, Ratschen E. A Systematic Review of Mental Health Professionals, Patients, and Carers' Perceived Barriers and Enablers to Supporting Smoking Cessation in Mental Health Settings. Nicotine & tobacco research : official journal of the Society for Research on Nicotine and Tobacco. 2022;24(7):945-54.
7. Juel A, Berring LL, Hybholt L, Erlangsen A, Larsen ER, Buus N, et al. Relatives' experiences of providing care for individuals with suicidal behaviour conceptualized as a moral career: A meta-ethnographic study. International Journal of Nursing Studies. 2021;113.
8. Lagdon S, Grant A, Davidson G, Devaney J, Donaghy M, Duffy J, et al. Families with Parental Mental Health Problems: A Systematic Narrative Review of Family‐Focused Practice. Child Abuse Review. 2021;30(5):400-21.
9. Liu Y-C. Caregiving experience associated with unpaid carers of persons with schizophrenia: a scoping review. Social Work in Mental Health. 2024;22(2):219-45.
10. Ma N, Roberts R, Winefield H, Furber G, Alderfer MALKALEAMALONLHJMELJABP. Utility of qualitative metasynthesis: Advancing knowledge on the wellbeing and needs of siblings of children with mental health problems. Qualitative Psychology. 2015;2(1):3-28.
11. Maude P, James R, Searby A. The use of Open Dialogue in Trauma Informed Care services for mental health consumers and their family networks: A scoping review. Journal of Psychiatric & Mental Health Nursing (John Wiley & Sons, Inc). 2024;31(4):681-98.
12. May K, Van Hooff M, Doherty M, Iannos M. How a systematic review of the experiences of emergency first responder family members living with post-traumatic stress disorder can inform new models of care. JBI Evidence Synthesis. 2023;21(4):627-8.
13. Munchenberg PS, Yessimova D, Panteli D, Kurth T. Digital Health Interventions for Informal Family Caregivers of People With First-Episode Psychosis: Systematic Review on User Experience and Effectiveness. JMIR Mental Health. 2024;11:e63743.
14. Noiriel A, Verneuil L, Osmond I, Manolios E, Revah-Levy A, Sibeoni J, et al. The lived experience of first-episode psychosis: A systematic review and metasynthesis of qualitative studies. Psychopathology. 2020;53(5-6):223-38.
15. Ntsayagae EI, Poggenpoel M, Myburgh C. Experiences of family caregivers of persons living with mental illness: A meta-synthesis. Curationis. 2019;42(1):e1-e9.
16. 56.	Oluwoye O, Cheng SC, Fraser E, Stokes B, McDonell MG, Addington JBPAAJCAMAADAAJHRKRDGSNR, et al. Family experiences prior to the initiation of care for first-episode psychosis: A meta-synthesis of qualitative studies. Journal of Child and Family Studies. 2020;29(9):2530-41.
17. Rask M, Schmidt M, Brolin R, Tuvesson H. Children's Perceptions and Experiences of Their Involvement in Their Parents' Mental Health Care - A Qualitative Literature Review. Issues in Mental Health Nursing. 2024;45(7):676-85.
18. Rowaert S, Vandevelde S, Lemmens G, Vanderplasschen W, Vander Beken T, Vander Laenen F, et al. The role and experiences of family members during the rehabilitation of mentally ill offenders. International Journal of Rehabilitation Research. 2016;39(1):11-9.
19. Shiraishi N, Reilly J, American Psychiatric Association ASLSSHEMFAVJAAAGVLNABAG. Positive and negative impacts of schizophrenia on family caregivers: A systematic review and qualitative meta-summary. Social Psychiatry and Psychiatric Epidemiology: The International Journal for Research in Social and Genetic Epidemiology and Mental Health Services. 2019;54(3):277-90.
20. Sibeoni J, Orri M, Colin S, Valentin M, Pradere J, Revah-Levy A, et al. The lived experience of anorexia nervosa in adolescence, comparison of the points of view of adolescents, parents, and professionals: A metasynthesis. International Journal of Nursing Studies. 2017;65:25-34.
21. Simes D, Shochet I, Murray K, Sands IG, Aggarwal SPGRNSSABMAAJSHCAWTAAUKK, Prevention NCfHSCASPCDNIPCSDCCSCCSTPMA, et al. A systematic review of qualitative research of the experiences of young people and their caregivers affected by suicidality and self-harm: Implications for family-based treatment. Adolescent Research Review. 2022;7(2):211-33.
22. Sin J, Norman I. Psychoeducational interventions for family members of people with schizophrenia: a mixed-method systematic review. The Journal of Clinical Psychiatry. 2013;74(12):e1145-62.
23. Tungpunkom P, Napa W, Chaniang S, Srikhachin P. Caregiving experiences of families living with persons with schizophrenia: a systematic review. JBI Database of Systematic Reviews & Implementation Reports. 2013;11(8):415-564.
24. Vusio F, Thompson A, Birchwood M, Clarke L, Adolescent Psychiatry Commitee Aacap Us ARSAAAKBAABHAABDHCLRBBWP, et al., et al. Experiences and satisfaction of children, young people and their parents with alternative mental health models to inpatient settings: A systematic review. European Child & Adolescent Psychiatry. 2020;29(12):1621-33.
25. Wadephul F, Jones C, Jomeen J. The Impact of Antenatal Psychological Group Interventions on Psychological Well-Being: A Systematic Review of the Qualitative and Quantitative Evidence. Healthcare (Basel, Switzerland). 2016;4(2).
26. Wirsen E, Akerlund S, Ingvarsdotter K, Hjarthag F, Ostman M, Persson K. Burdens experienced and perceived needs of relatives of persons with SMI - a systematic meta-synthesis. Journal of mental health (Abingdon, England). 2020;29(6):712-21.
27. Young L, Murata L, McPherson C, Jacob JD, Vandyk AD. Exploring the Experiences of Parent Caregivers of Adult Children With Schizophrenia: A Systematic Review. Archives of Psychiatric Nursing. 2019;33(1):93-103.

**Wrong study design (n = 9)：**

1. Doody O, Butler MP, Lyons R, Newman D. Families' experiences of involvement in care planning in mental health services: an integrative literature review. Journal of Psychiatric and Mental Health Nursing. 2017;24(6):412-30.
2. Jeon Y, Brodaty H, Chesterson J. Respite care for caregivers and people with severe mental illness: literature review. Journal of Advanced Nursing (Wiley-Blackwell). 2005;49(3):297-306.
3. Kumar G, Sood M, Verma R, Mahapatra A, Chadda RK, Amaresha ACVGMDABVCVBCAJCCRCCRDKCG. Family caregivers' needs of young patients with first episode psychosis: A qualitative study. International Journal of Social Psychiatry. 2019;65(5):435-42.
4. Miller J, Nguyen E, Lam AYH, Brann P, Innes S, Buntine P, et al. Experiences of consumers, carers and clinicians during borderline personality disorder presentations to the emergency department—An integrative review. Journal of Psychiatric & Mental Health Nursing (John Wiley & Sons, Inc). 2024;31(4):572-82.
5. Nic Dhonnacha S, Kerr L, McCague Y, Cawley D. Parents' Experiences of Accessing Mental Health Services for Their Adolescents With Mental Health Challenges: A Scoping Review. Journal of Clinical Nursing. 2024;33(12):4602-17.
6. Ray S. Acute inpatient mental health services: Carers experiences and implementing method of levels therapy for those experiencing suicidality. Dissertation Abstracts International Section A: Humanities and Social Sciences. 2025;86(6-A):No-Specified.
7. Sibeoni J, Orri M, Valentin M, Podlipski M-A, Colin S, Pradere J, et al. Metasynthesis of the Views about Treatment of Anorexia Nervosa in Adolescents: Perspectives of Adolescents, Parents, and Professionals. PLoS ONE. 2017;12(1):e0169493.
8. Tungpunkom P, Srikhachin P, Napa W, Chaniang S. Caregiving Experiences of Families Living with Patients with Schizophrenia: a Systematic Review. JBI library of systematic reviews. 2012;10(28 Suppl):1-8.
9. Walters C, Petrakis M. A Systematic Mapping Review of Family Perspectives About Received Mental Health Interventions. Research on Social Work Practice. 2022;32(1):61-72.

**Wrong population (n = 21)：**

1. Anker‐Hansen C, Skovdahl K, McCormack B, Tønnessen S. The third person in the room: The needs of care partners of older people in home care services—A systematic review from a person‐centred perspective. Journal of Clinical Nursing (John Wiley & Sons, Inc). 2018;27(7-8):e1309-e26.
2. Bacon G, Sweeney A, Batchelor R, Grant C, Mantovani N, Peter S, et al. At the Edge of Care: A Systematic Review and Thematic Synthesis of Parent and Practitioner Views and Experiences of Support for Parents with Mental Health Needs and Children's Social Service Involvement. Health & Social Care in the Community. 2023:1-33.
3. Baudinet J, Eisler I, Dawson L, Simic M, Schmidt U. Multi-family therapy for eating disorders: A systematic scoping review of the quantitative and qualitative findings. The International journal of eating disorders. 2021;54(12):2095-120.
4. Dehbozorgi R, Shahriari M, Fereidooni-Moghadam M, Moghimi-Sarani E. Family-centered collaborative care for patients with chronic mental illness: A systematic review. Journal of Research in Medical Sciences. 2023;28(2):6-.
5. DeLeo K, Maconick L, McCabe R, Broeckelmann E, Sheridan Rains L, Rowe S, et al. Experiences of crisis care among service users with complex emotional needs or a diagnosis of 'personality disorder', and other stakeholders: systematic review and meta-synthesis of the qualitative literature. BJPsych Open. 2022;8(2):e53.
6. Forde R, Peters S, Wittkowski A, Alhusen JLHMJGDABVBKMKKMKAMHWJL-. Recovery from postpartum psychosis: A systematic review and metasynthesis of women's and families' experiences. Archives of Women's Mental Health. 2020;23(5):597-612.
7. Gill J, Jones A, Price K, Goodison E, Tyson P. A mixed‐method systematic review of the perspectives of young people, carers and professionals on psychiatric diagnosis in childhood and adolescence. Journal of Child & Adolescent Psychiatric Nursing. 2024;37(2):1-14.
8. Greer H, Cohen JN, AgardAs ETLAAPAACABGBBGBBGBBDSSBBWRPDZ, et al., et al. Partners of individuals with borderline personality disorder: A systematic review of the literature examining their experiences and the supports available to them. Harvard Review of Psychiatry. 2018;26(4):185-200.
9. Gustafsson SA, Stenstrom K, Olofsson H, Pettersson A, Wilbe Ramsay K. Experiences of eating disorders from the perspectives of patients, family members and health care professionals: a meta-review of qualitative evidence syntheses. Journal of Eating Disorders. 2021;9(1):156.
10. Izon E, Radez J, Knight MTD, Al-Sawafi ALKRLHNA-SASALKRLHNASAACJ. The psychosocial stressors of siblings of people with experiences of psychosis (SOPEP): A systematic narrative review across cultures. Clinical Psychology & Psychotherapy. 2024;31(1):No-Specified.
11. Johns G, Taylor B, John A, Tan J, Anderson KAECKKRGDLABDARRWSLRRES, et al., et al. Current eating disorder healthcare services-The perspectives and experiences of individuals with eating disorders, their families and health professionals: Systematic review and thematic synthesis. BJPsych Open. 2019;5.
12. Lamont E, Dickens GL. Mental health services, care provision, and professional support for people diagnosed with borderline personality disorder: systematic review of service-user, family, and carer perspectives. Journal of Mental Health. 2021;30(5):619-33.
13. Loughlin M, Bucci S, Brooks J, Berry K, Allard JLSCSATBMAAKKFRMAKAAAGVL. Service users' and carers' experiences of engaging with early intervention services: A meta-synthesis review. Early Intervention in Psychiatry. 2020;14(1):26-36.
14. MacDonald K, Ferrari M, Fainman-Adelman N, Iyer SN, Aisbett DBCFKJNKNKAAADOBMEDOLAAKF. Experiences of pathways to mental health services for young people and their carers: A qualitative meta-synthesis review. Social Psychiatry and Psychiatric Epidemiology: The International Journal for Research in Social and Genetic Epidemiology and Mental Health Services. 2020:No-Specified.
15. Martin F, Dahmash D, Wicker S, Glover S, Duncan C, Anastassiou A, et al. Systematic review with qualitative meta-synthesis of parents' experiences and needs in relation to having a child or young person with a mental health difficulty. BMJ mental health. 2025;28(1).
16. Maybery D, Reupert A. Parental mental illness: a review of barriers and issues for working with families and children. Journal of Psychiatric & Mental Health Nursing (Wiley-Blackwell). 2009;16(9):784-91.
17. Pereira TLB, Wichaikhum OA, Nantsupawat A, Rajendrana P, Baladram S, Shorey S. Recognising the Parental Caregiver Burden of Children With Mental Disorders: A Systematic Mixed‐Studies Review. International Journal of Mental Health Nursing. 2024;33(6):1941-61.
18. Reardon T, Harvey K, Baranowska M, O'Brien D, Smith L, Creswell C, et al. What do parents perceive are the barriers and facilitators to accessing psychological treatment for mental health problems in children and adolescents? A systematic review of qualitative and quantitative studies. European Child & Adolescent Psychiatry. 2017;26(6):623-47.
19. Steiro AK, Iversen HH. Important aspects of experiences from patients and parents related to medications in Child and Adolescents Mental Health Services (CAMHS) - a qualitative study. BMC Psychology. 2024;12(1):458.
20. Wells J, Kennedy C, Bain H, Lee SH, Abdul-Hamid WKL-CKHFSAMA-HADAADBSBACPJ, Blood Institute NCHPJNPLACVFDSSMHFDJDVP. The experiences of older adults with a diagnosed functional mental illness, their carers and healthcare professionals in relation to mental health service delivery: An integrative review. Journal of Clinical Nursing. 2020;29(1-2):31-52.
21. Wyatt C. Postnatal mental distress: Exploring the experiences of professionals, mothers, and significant others. Dissertation Abstracts International: Section B: The Sciences and Engineering. 2021;82(6-B):No-Specified.

**Reviews on informal carers’ experiences in general (n = 27)：**

1. Alyafei AH, Alqunaibet T, Mansour H, Ali A, Billings J, Abdelmoneium AORJCMAAHWPAAMAMIAMJLVCM, et al. The experiences of family caregivers of people with severe mental illness in the Middle East: A systematic review and metasynthesis of qualitative data. PLoS ONE. 2021;16(7).
2. Casaleiro T, Caldeira S, Cardoso D, Apostolo J, Ahmad Ramli FZTCWJARANMDDMGNMRJABEC, Quality CMEACCMWSHGEMLKRCCLSMCD-OM. Spiritual aspects of the family caregivers' experiences when caring for a community-dwelling adult with severe mental illness: A systematic review of qualitative evidence. Journal of Psychiatric and Mental Health Nursing. 2022;29(2):240-73.
3. Cleary M, West S, Hunt GE, McLean L, Kornhaber R. A Qualitative Systematic Review of Caregivers' Experiences of Caring for Family Diagnosed with Schizophrenia. Issues in Mental Health Nursing. 2020;41(8):667-83.
4. Fox JRE, Dean M, Whittlesea A, Apple RFAARSKDKPLKMEABABBJGCSM, Care Excellence NGHRNPCSCDGVBFPPKFJREPPS. The experience of caring for or living with an individual with an eating disorder: A meta-synthesis of qualitative studies. Clinical Psychology & Psychotherapy. 2017;24(1):103-25.
5. Greally S, Oute J, McPherson S, Ahlstrom BHSIDEAAGFDVAGAYAAOOASLCA. Experiences of parents of children with mental illnesses: A systematic review and meta-ethnographic synthesis. Family Process. 2024:No-Specified.
6. Heneghan A, Manitsa I, Livanou M, Treasure J, Abrams MSAAPAADM-PCSARTJAAAAAAAAR, Care Excellence PSWSMJMRSUPPAGSGCMMRBCPQ. The experiences of having a sibling with an eating disorder: A systematic review of the literature. European Eating Disorders Review. 2024;32(3):382-403.
7. Hennipman-Herweijer C, Amerongen-Meeuse JvN, Schaap-Jonker H, Boonstra N, Adelman RDTLLDDDSLMSAASCTLHCMXL. The experiences of living with a suicidal family member, and the impact on daily life: A systematic review and meta-aggregation. Journal of Psychiatric and Mental Health Nursing. 2024;31(5):883-903.
8. Ho LL, Li Y, Gray R, Ho GW, Bressington D. Experiences and views of carers regarding the physical health care of people with severe mental illness: An integrative thematic review of qualitative research. Journal of Psychiatric and Mental Health Nursing. 2021;29(6):774-87.
9. Issac A, Nayak SG, Yesodharan R, Sequira L. Needs, challenges, and coping strategies among primary caregivers of schizophrenia patients: A systematic review & meta-synthesis. Archives of Psychiatric Nursing. 2022;41:317-32.
10. Kageyama M. The Process of Family Experiences in Caring for Persons with Serious Mental Illnesses: Common Stages Identified through a Literature Review. Journal of Japan Academy of Nursing Science. 2012;32(4):64-70.
11. Kallquist A, Salzmann-Erikson M, Andersson GTNAARHJGCGRABSYBBJBD-CAJS. Experiences of having a parent with serious mental illness: An interpretive meta-synthesis of qualitative literature. Journal of Child and Family Studies. 2019;28(8):2056-68.
12. Lascelles KM, Davey Z, Jackson D, Aveyard H. Experiences and needs of adult informal carers of adults at risk of suicide: A systematic review with mixed methods analysis. Journal of Advanced Nursing. 2024;80(5):1686-718.
13. Lavers G, Andriessen K, Krysinska K. A Systematic Review of the Experiences and Support Needs of Informal Caregivers for People Who Have Attempted Suicide or Experienced Suicidal Ideation. International journal of environmental research and public health. 2022;19(9).
14. ling Xiao BAI, yang Zhao Z, zhen Zeng Y, Ting LOU, yuan Si T. The lived experience of family members caring for schizophrenia patients at home:a systematic review of qualitative studies. Chinese Journal of Nursing. 2017(12):1486-94.
15. Marchetti D, Sawrikar V, American Psychiatric Publishing BSCC-RMHNFLTJSPSCOJSBBVZ, Care Excellence ODLODOEEORISHRPRRRGJYIWB. Parents' illness representations of their child with anorexia nervosa: A systematic review of qualitative studies using the common-sense model. International Journal of Eating Disorders. 2024;57(5):1049-68.
16. Marshall P, Sansom K, Jagfeld G, Jones S, Lobban F, Allan NPBJWRAMSNBAA-DWOJACSA-DAY, et al. Caring for a friend or family member who has experienced suicidal behaviour: A systematic review and qualitative synthesis. Psychology and Psychotherapy: Theory, Research and Practice. 2023;96(2):426-47.
17. McGaw VE, Reupert AE, Maybery D, Ackerson BAA-TFAOJUA-TAPABKNKSAWLASK. Military posttraumatic stress disorder: A qualitative systematic review of the experience of families, parents and children. Journal of Child and Family Studies. 2019;28(11):2942-52.
18. Mui EYW, Chan SKW, Chan PY, Hui CLM, Chang WC, Lee EHM, et al. Systematic review (meta-aggregation) of qualitative studies on the experiences of family members caring for individuals with early psychosis. International Review of Psychiatry. 2019;31(5-6):491-509.
19. Neely L. Exploring experiences of parents of individuals with an eating disorder. Dissertation Abstracts International Section A: Humanities and Social Sciences. 2025;86(5-A):No-Specified.
20. Panes II, Tuppal CP, Reñosa MDC, Baua MEP, Vega PD. Family Experiences of Mental Illness: A Meta-Synthesis. Nurse Media Journal of Nursing. 2018;8(2):102-12.
21. Rodriguez J, Radjack R, Moro MR, Lachal J. Migrant adolescents' experience of depression as they, their parents, and their health-care professionals describe it: A systematic review and qualitative meta-synthesis. European Child & Adolescent Psychiatry. 2022:No-Specified.
22. Rowe J. Great expectations: a systematic review of the literature on the role of family carers in severe mental illness, and their relationships and engagement with professionals. Journal of Psychiatric & Mental Health Nursing (John Wiley & Sons, Inc). 2012;19(1):70-82.
23. Senior E, Clarke A, Wilson-Menzfeld G, AllenE.S RKSMMJAADaLRiH, Social Care ABCLLFECBiPoVWVwPSDBBLSBAJ, Psychological Distress in Partners of Veterans with Chronic Posttraumatic Stress Disorder CCBRDCCN-PJCDAASDDJ, et al. The military spouse experience of living alongside their serving/veteran partner with a mental health issue: A systematic review and narrative synthesis. PLoS ONE. 2023;18(5).
24. Susanti H, Lovell K, Mairs H. What does the literature suggest about what carers need from mental health services for their own wellbeing? A Systematic Review. Enfermería Clínica. 2018;28:102-11.
25. Tay JY, Li Z, Goh YS. Experiences of Informal Caregivers Caring for Individuals With Chronic Schizophrenia in Asia: A Systematic Review and Meta-Synthesis. Journal of Psychiatric and Mental Health Nursing. 2025;32(2):487-563.
26. Umer M, Tong HYCC, Rodak T, Xu C, Dennis CL, Naeem F, et al. Mapping global evidence of caregiver experiences in bipolar disorder in low and middle-income countries: A scoping review. Journal of Affective Disorders. 2025;374:191-215.
27. Zhao Y-l, Liu Z-h, Li Y-y, Liu D-l, Yi J-n. The lived experiences of parents providing care to young people who self-harm: A meta-aggregative synthesis of qualitative studies. International Journal of Mental Health Nursing. 2022:No-Specified.

**C. Findings extracted from each included review (Tables 1-12)**

This appendix contains 12 tables, each summarising findings from one of the 12 included reviews. **The first column** includes a summary of the main findings, their credibility rating, and the original study reference. **The second column** provides verbatim excerptsfrom the source reviews (i.e. text copied directly from the included reviews without modification, representing the original descriptions of findings reported by the review authors), and **the third column** presents direct quotes from carers cited in the reviews to support the findings. This structure ensures clarity, transparency, and a clear link between the findings, original text, and carers’ quotes.

Credibility rating:

Unequivocal (U): supported by clear, direct quotes or observations.

Equivocal (E): logically inferred but less directly supported.

Unsupported (US): findings not supported.

* The finding was derived from a theme that also gave rise to another finding in the original review. (Findings typically reflected a single theme or subtheme, though in a few cases one theme yielded two findings. These instances were documented and marked with an asterisk (*) to maintain transparency and avoid overrepresentation in the synthesis.)

**Abou Seif N, Wood L, Morant N. Invisible experts: a systematic review & thematic synthesis of informal carer experiences of inpatient mental health care. BMC Psychiatry. 2022;22(1):347.**

| **Findings, credibility rating and citations** | **Verbatim excerpts of the source reviews** | **Cited illustrative quotes** |
| --- | --- | --- |
| The build up to hospitalisation was distressing, overwhelming, powerless, and frustrated. (U)  AbouSeif et al., 2022 | The build up to hospitalisation was described as distressing and overwhelming, ... Carers report feeling powerlessness and frustration that help was seemingly only made available once their loved one had deteriorated to a point where hospitalisation was inevitable. | “I need help now not like tomorrow or next week. I think like they got back to me three months later, it was really, really hard to get any kind of help to start with.” |
| Carers reported a mixture of emotions such as relief, guilt, fear, and hope upon their loved one’s hospitalisation. (U)  AbouSeif et al., 2022 | Once their loved one was hospitalised, carers reported experiencing a mixture of conflicting emotions such as relief, guilt, fear, and hope. | “Participants’ accounts of hospitalisation framed it overwhelmingly as an appropriate intervention that brought relief and respite.”  “The mothers expressed that they often felt as if they were being judged as parents who were trying to ‘get rid of the problem.’” |
| Carers were seldom acknowledged or supported. (U)  AbouSeif et al., 2022 | Despite the significant emotional toll of the hospitalisation and a heightened need for support, carers report that their needs were seldom acknowledged and strongly perceived inpatient staff as unsupportive. They were not offered support or asked about their own needs by staff. | “Interestingly, the families clearly did not perceive staff as being supportive … they were seldom, if ever, acknowledged when they visited their child” |
| Carers felt excluded from inpatient care. (U)  AbouSeif et al., 2022 | ..., carers felt excluded from inpatient care. Carers reported being informed of decisions regarding their loved one’s care last minute or after the fact, ... Carers felt that their expertise was not drawn upon or included in clinical decision making. | “I wasn’t involved, I was an afterthought . . . no one  told us anything, no one rang to keep us up to date with the plan of care.” |
| Carers expressed dissatisfaction with multiple aspects of mental health services. (U)  AbouSeif et al., 2022 | Carers spoke of dissatisfaction relating to delays in admission, unmet service user needs, staff competence, the duration of hospitalisation, safety and the lack of space. ... Carers were also displeased with the heavy focus on medicating the service user, | “That’s all she gets. She’s never actually sat down with anybody and just talked about anything.” |
| Hospitalisation led to a strain on the relationship between carers and service users. (U)  AbouSeif et al., 2022 | Carers reported that with the hospitalisation, a strain was placed on their relationship with the service user. … For some, this was linked to blame, ... Others cited an inability to visit their loved one as often as desired due to practical difficulties, ... | “He had this great hatred of me, whatever it was, so it was very difficult for me, it was a great hate. And I think it stemmed from I was the one, I actually put him into the hospital.” |
| Carers felt a tension between staff and themselves, while partnership and cooperation were desired. (U)  AbouSeif et al., 2022 | They described the relationship as marked with tension and reported sensing a divide of “us vs them”, wherein they were viewed as threats, challenging, or nuisances. Nonetheless, carers consistently expressed a desire for partnership with staff, … | “It’s about working together, the team knowing that I have valuable things to contribute and vice versa,  because we all want the same at the end of the day.” |
| Carers wanted staff to be more affectionate and caring to their loved ones. (U)  AbouSeif et al., 2022 | They spoke of wanting staff to be more affectionate and caring to their loved one, to actively listen and talk to them, and to help them with self-care. | “All she does is see a doctor once or twice a week. There’s no counsellor brought in … She seriously needs to talk to somebody” |

**Almoaber Z, Moxham L, Patterson C. Experiences of respite care among carers or relatives who are responsible for caring for individuals with a mental illness: an integrative literature review. International Journal of Mental Health Nursing. 2024;33(6):2267-83.**

| **Findings, credibility rating and citations** | **Verbatim excerpts of the source reviews** | **Cited illustrative quotes** |
| --- | --- | --- |
| Most informal carers experienced relief during their use of respite care. (U)  Almoaber et al., 2024 | […] most of the informal carers experienced relief from burden during their use of respite care […] services that offer respite care provide the necessary security for informal carers to place their family members in care. Furthermore, this peace of mind enables carers to perceive the respite experience positively. | “One carer emphasised ‘feeling peace of mind that their loved one was safe’.” |
| The aviailability of respite care services is limited. (U)  Almoaber et al., 2024 | Carers described the availability of respite care services, in general, as being limited, … | ‘There isn’t any suitable respite … [the] few services that are around just don’t offer what young adults with depression and anxiety want’ and ‘We do not have a respite situation in our city which would be suitable’. |
| Carers’ a lack of knowledge and understanding of respite care is a barrier to utilising the services. (E)  Almoaber et al., 2024 | Carers consider a lack of knowledge and understanding of respite care a common barrier to utilising respite services … | No quotes. |
| Entering respite care was restricted by specific criteria, which negatively impacted the access. (E)  Almoaber et al., 2024 | ... entering respite care was restricted by the use of specific criteria such as the patient’s health condition, diagnosis, geographical location, age, length of the caring role and ethnicity. All these restrictions had the potential to negatively impact access to respite care due to a lack of flexibility for carers to meet the demands that respite service providers levied .... | No quotes. |
| The relectance of patients with mental health difficulties to services is a significant barrier to accessing respite services. (E)  Almoaber et al., 2024 | The reluctance on the part of patients with MI towards using respite care is a significant barrier to accessing respite services. 74% of their participating carers indicated that people with MI had rejected respite opportunities. | No quotes. |
| Carers experienced increased stress, shame, stigma, guilt, resentment, grief, and worry during the process of accessing respite care. (E)  Almoaber et al., 2024 | ... the subjective burden (shame, stigma, guilt, resentment, grief, worry) remained unaffected. Regarding this aspect, carers reported experiencing an increased stress level. | No quotes. |
| Carers viewed staff in respite services as having insufficient skills. (U)  Almoaber et al., 2024 | … carers viewed staff who worked in respite services as having insufficient skills to care for people with MI | “For example, one carer specified that the ‘respite worker was not able to provide a sense of safety/care’”. |
| Carers described respite services as inadequately tailored to service users’ needs. (E)  Almoaber et al., 2024 | Carers and patients with MI in the studies under consideration described respite services as providing care for a variety of people simultaneously, [...] the activities offered tended to be unsuitable for all participants … | No quotes. |
| There is a general lack of attention to cultural diversity in current services. (E)  Almoaber et al., 2024 | This review also uncovered a general lack of attention to cultural diversity because of the inappropriate service model for respite care. ... A lack of cultural understanding among mental health workers and regarding Indigenous Australians has also raised a barrier to accessing respite care. | No quotes. |

**Bartl G, Stuart R, Ahmed N, Saunders K, Loizou S, Brady G, et al. A qualitative meta-synthesis of service users’ and carers’ experiences of assessment and involuntary hospital admissions under mental health legislations: a five-year update. BMC psychiatry. 2024;24(1):476.**

| **Findings, credibility rating and citations** | **Verbatim excerpts of the source reviews** | **Cited illustrative quotes** |
| --- | --- | --- |
| Carers reported fear, high stress, hypervigilance in the build-up to a service user’s crisis, and stress and frustration in the build-up to services. (U)*  Bartl et al., 2024 | Carers reported fear about service users’ symptoms and behaviour prior to detention, high stress and hypervigilance in the build-up to a service user’s crisis; and frustration when health professionals ignored or ‘failed to grasp the gravity of the service user’s illness’. | “The actual sectioning process was about as horrible as it ever could have been. It was possibly the worst experience of my life …” |
| Carers felt bad and distressing assessment and admission. (U)*  Bartl et al., 2024 | Carers felt bad about initiating coercive measures and found assessment distressing, and admission traumatizing. | “The actual sectioning process was about as horrible as it ever could have been. It was possibly the worst experience of my life …” |
| Some carers felt some relief upon their loved one’s admission. (U)  Bartl et al., 2024 | Following admission, some carers felt some relief because they were reassured that the risk of harm to self or others had been averted, and the service user was now safe and in receipt of care. | “A member of the public called in once. I felt relieved it wasn’t us that time. When he recovered, we could face him and tell him we had nothing to do with it.” |
| Detention caused emotional strain and reduced carers’ psychological wellbeing. (U)  Bartl et al., 2024 | Personal uncertainty, disagreements with other family members about the right course of action, and differences between what the person they cared for wanted and what was in their best interests, all took a toll on carer wellbeing. | “As I’m leaving he’s crying out, ‘Mom, why you doing this to me? I don’t want to be here, don’t leave me here.’” |
| Carers needed proactive support. (U)  Bartl et al., 2024 | For their own health, carers need support to be offered proactively, before, during and after detention, to help them make sense of the illness, deal with stress, and accept their situation. | “One minute there were police cars and half a dozen doctors and lots of shouting and kind of stuff going on and then the next minute I was just here on my own and that was a bit kind of challenging, difficult.” |
| Carers reported a lack of information and guidance from health services during help seeking. (U)  Bartl et al., 2024 | Carers reported that there was a lack of information and guidance from health services as they managed a family member with deteriorating health and sought help. | “The whole [assessment] experience was pretty traumatic really I suppose. There should be more support actually for me or actually tell me what I need to do to support him.” |
| Carers felt a strong sense of duty, supporting their relatives before, during, and after hospitalisation. (U)  Bartl et al., 2024 | Carer participants expressed a sense of duty towards their family member which they willingly took on. While some carers initiated or advocated for admission, some also remained vigilant to protect their relative following admission and resumed 24-hour responsibility following discharge. | “The phone calls, oh, they’ve taken an overdose or I’m going to go under a bus or - they [health professionals] don’t seem to have any real regard for the fact that there are people out here that are having to deal with all this stuff.” |
| Carers’ familiarity with the service users gave them expertise. (U)  Bartl et al., 2024 | Carers’ familiarity with the relative they care for, having been with them when they were well and during previous episodes of illness, and knowing the circumstances of the latest admission, gives carers expertise. | “He’s been with me all his life.”  “I know what she was like before.” |
| Carers attempted to participate in care and decision making, but often failed. (U)  Bartl et al., 2024 | Following involuntary admission carers reported that they tried to maintain dialogue with hospital staff and participate in treatment and discharge decisions but were often not being heard. | “… Nearest Relatives are not routinely consulted or provided with information once the hospital admission takes place.” |
| Carers wanted to share and receive information, while the absence of information was frequently reported. (U)  Bartl et al., 2024 | To maximise treatment and recovery, carers want to share information and described the importance of receiving information from clinicians about the illness and the service user’s progress. Carers frequently reported the absence of information … | “While the treatment team relied on them for information, they were not informed about their relatives’ progress in return.” |
| Confidentiality prevented carers from receiving information. (U)  Bartl et al., 2024 | Carers stated that confidentiality prevented them from receiving information that would help them take care of their relative. | “Some disclosed that this [confidentiality] clause was being used purposively to inhibit family involvement. Furthermore, a subsection of these caregivers stated that being denied access to such information went against their rights.” |
| Some carers described experiences of power in their role and the power unbalanced their relationship with their family members. (U)  Bartl et al., 2024 | Carers described experiences of power in their role. ... Some carers felt distressed that their powers as a Nearest Relative [...] unbalanced their relationship with their relative ... Many carers felt powerless, but some viewed their relative’s admission as forced by them and some used their relative’s economic dependency as leverage to engage in treatment. | “Caregivers predominantly viewed their relative’s admission as forced and imposed by them rather than led by the service user or mental health services.” |
| Carers wanted partnership with health professionals and appreciated kind and respectful professionals, while some reported neglect and unpleasant interactions. (U)  Bartl et al., 2024 | Carers wanted to be able to work in partnership with health professionals and appreciated professionals who were kind and respectful. Others experienced staff as lacking empathy, compassion, or time to talk; and failing to listen or take carers seriously. | “One participant praised the AMHPs [Approved Mental Health Professionals - clinicians involved in assessment for detention in hospital in England] she had spoken to as being ‘fair’ and ‘lovely’” |
| Carers mediated between service users and health professionals. (U)  Bartl et al., 2024 | Some carers described mediating between the person they care for and health professionals. | “If you haven’t got someone fighting from the outside inwards, well you’re just left to your own devices. […] If she did not have any visitors, she could be bruised and bullied.” |
| Some studies described relationship break-down between carers and service users following involuntary admission. (U)  Bartl et al., 2024 | Carers spoke of trying to make sense of the illness in the context of family relationships. … some studies described communication breaking down between carers and service users following involuntary admission: mistrust and rejection by service users; losing touch; or carers cutting off contact after admission ... | “Some interviewees reflected on their family relationships. They suspected that this also played a role in the behaviour of their ill family member.” |
| Community services and pathways to care were inadequate. (U)  Bartl et al., 2024 | Several studies describe insufficient community services [...] dismissed carer concerns until the service user was acutely unwell. … Some carers reported that pathways to care were inadequate. | “… the need for a full medical assessment in service users who are acutely mentally unwell and have a standing history of mental illness”, and in Connecticut “caregivers argued against the need for probate court hearings in order to obtain a commitment order” |
| Carers felt let down by heavy-handed detention but valued respectful treatment. (U)  Bartl et al., 2024 | The previous review reported that by the time assessment and detention occurred, many carers already felt let down by services. Carers described detention processes as inappropriate/heavy-handed...  Recent reports are also mixed. Carers appreciated a street triage initiative and service users being treated with respect. Carers also reported a lack of community alternatives to inpatient admission, concern about how involuntary admission was managed. | “I didn’t have problems with the involuntary treatment. Without the involuntary treatment […] how would she have gotten the treatment? But it’s just how the service  provider carried it out [which] is probably the bigger issue.” |
| Some carers did not trust the services and treatments. (U)  Bartl et al., 2024 | Carers wanted to know that their relatives are safe and receiving care to help them recover, but some carers did not believe that the hospital met their relative’s needs and did not trust the treatment provided. … | “All she does is see a doctor once or twice a week. There’s no counsellor brought in […] She seriously needs to talk to somebody, not for 10 minutes, how you’re going, how  you’re feeling, are you still seeing anything? That’s all she gets. She’s never actually sat down with anybody and just talked about anything.” |
| Many carers reported to be informed about discharge at the last minute. (U)  Bartl et al., 2024 | ... many carers reported that they were informed about discharge at the last minute when neither the service user nor family was ready. | “No discharge plan or anything. They didn’t even explain the medications to us, how he would take it, what times to give it to him. Just said ‘Bye’. They gave us a bag of medication.” |
| Carers’ fear and unwillingness were barriers to help-seeking and accessing services. (U)  Bartl et al., 2024 | Carers had difficulties arising from their relatives’ subsequent fear and unwillingness to seek help and engage with services, withdrawing and keeping their distance from carers; being guarded and masking their symptoms during assessments. | “… the effect was unsustainable and had not changed the insight, and because of this experience, patients were even more negative regarding compliance with treatment recommendations.” |

**Cairns VA, Reid GS, Murray C. Family members’ experience of seeking help for first‐episode psychosis on behalf of a loved one: A meta‐synthesis of qualitative research. Early intervention in psychiatry. 2015;9(3):185-99.**

| **Findings, credibility rating and citations** | **Verbatim excerpts of the source reviews** | **Cited illustrative quotes** |
| --- | --- | --- |
| Upon noticing changes in loved ones, carers experience a sense of ‘not knowing’ upon noticing changes in loved ones characterized by a sense of confusion, frustration, and desperation. (U)*  Cairns et al., 2015 | Upon noticing changes in their loved ones, family members experienced a sense of ‘not knowing’ … This experience was characterized by a sense of desperation … | “… we knew something was terribly wrong, we didn’t have a name for it.” |
| Upon noticing changes in their loved ones, informal carers struggle to make sense of the situations and to cope. (U)*  Cairns et al., 2015 | Upon noticing changes in their loved ones, family members experienced a sense of ‘not knowing’ and attempted to make sense, understand the experience and seek answers to difficult questions. This experience was characterized by a sense of desperation while trying to meet the needs of their loved ones. | This often resulted in a ‘desperate search for someone who could suggest both an explanation and a remedy for the disturbing behaviours of their child’. |
| Personal coping resources became overwhelmed at ‘crisis point’ and led to a sharp increase in help-seeking behaviour. (U)  Cairns et al., 2015 | The reaching of crisis point within the help seeking experience was related to both limited knowledge and access to support, as well as families drawing upon personal coping resources before becoming overwhelmed. Crisis point was associated with a sharp increase in help-seeking behaviour by family members, a sense of relief at support being accessed and also concerns regarding that available. | “When it [psychosis] was first happening I mentioned it to my doctor [GP] but it wasn’t picked up … luckily, I had a pamphlet that someone gave me … and there was a phone number [FEP service] in it. I just rang it … within 12 hours they were on to it [care and treatment]. It was just fantastic … My doctor [GP] was … not alert enough. I won’t sit around sit around wasting time again to get a doctor’s [GPs] appointment and fit into her structure, because it was a real crisis.” |
| Seeking help for FEP on behalf of a loved one led to high levels of distress and sometimes a sense of trauma. (U)  Cairns et al., 2015 | The experience of seeking help for FEP on behalf of a loved one had a significant emotional impact upon family members, including high levels of distress and sometimes a sense of trauma. The help-seeking experience also impacted upon relationships, with both others and the self, often negatively, but at times (often later in the process) positively. | “I just couldn’t stop crying. It was horrible, I had to go to work, I’d go to work in tears, you know.”  “I know that this is not my fault. But the truth is, I feel like a failure at the most important, one of the most important things of my life.” |
| Interactions with informal networks, professionals, and other families mediated the help-seeking process, either facilitating it or hindering it. (U)  Cairns et al., 2015 | Interactions had a mediating effect upon attempts to seek help, ways in which this was approached and the impact upon the family member. .... Help seeking was facilitated where these interactions identified the need for help and involved clear communication and support from professionals, whereas interactions had a negative influence upon the process where others’ attempts to normalize subsequently minimized the significance, and/or became dismissing of difficulties. | “If I talked to people they would say “he sounds like a normal teenager to me.” You do sort of wonder . . you wonder whether this is what they mean by ‘stroppy obnoxious teenagers’ and so you put it down to that.”  “my experience with the psychiatrist . . . is that I felt really marginal to the whole process”. |

**Dawson S, Jordan Z, Attard M. Carers’ experiences of seeking help for relatives with first-episode psychosis: a systematic review of qualitative evidence. JBI Evidence Synthesis. 2013;11(11):183-361.**

| **Findings, credibility rating and citations** | **Verbatim excerpts of the source reviews** | **Cited illustrative quotes** |
| --- | --- | --- |
| Difficulties were gradually noticed by relatives. (U)  Dawson et al., 2013 | Various mood and behavioral changes, as well as general functional decline, alerted relatives to there being something “wrong” with the individual developing a psychotic illness. | “He was 15 or 16 when we started to get calls from college that he was having panic attacks. At sixth form he couldn’t sit in a class room but they didn’t help him. He’s tried to go into employment but things keep going wrong and he loses the job.” |
| Early symptoms are commonly misatributed to alternative explanations, which influence the type and timing of help-seeking. (U)  Dawson et al., 2013 | The early phase and symptoms of psychotic illnesses are commonly attributed to factors other than mental illnesses … The misattribution of symptoms impacts upon the type and timing of help-seeking … | “And he’d say things as if he was talking to himself. I just thought he was talking to himself. I mean, I go round the house talking to myself.” |
| Carers’ knowledge and belief influence their perceptions and the type of help that is sought. (U)  Dawson et al., 2013 | After a problem has been acknowledged, carers attempt to understand the problems within their own belief system. Many were unable to make sense of the presenting difficulties and were unsure of how to respond. | “And I was thinking it was just him being lazy and not wanting to do anything.”  “This can be exemplified by the case of a youth that will be called José. His mother was convinced that José’s problem had a supernatural causation: some ‘evil spirit’ was disturbing her son, or that he was the prey of some black magic perpetrated by her ex-lover. This belief motivated José’s mother to force her son to be exorcised in a Pentecostal church.” |
| Lack of knowledge of treatment and service options delayed help-seeking. (U)  Dawson et al., 2013 | Carers reported they were unaware of where to access help and how to negotiate access **…** | “I have a feeling I did try. I mean I spoke to friends about it. I didn’t even think to go to a psychiatrist; I wouldn’t even have known where to go. I mean I didn’t know this place [CIEIS] here existed. Had I known about it and had they had a helpline or something, I might have done that and I think that would be very, very good if they had a helpline for confused and worried parents or parents-in-law or friends.” |
| Relatives’ informal networks and the individuals’ wider context did not promote help-seeking.  Dawson et al., 2013 | ...the carers’ informal contacts either actively discouraged contact with mental health services or were noted as not advocating for or facilitating help-seeking. Informal networks included family members, relatives and friends as well as people in the individuals’ wider context including employers, educators and religious bodies. | “My mother would often get very irritated and say ‘what do you want us to do - put her in a mental asylum?’ like that was the most horrible thing one could say. […] My father used to say ‘oh let her go out into the world, she’ll soon be put in her place or something…’” |
| Stigma associated with having a mental illness and of mental health services and treatment delayed help-seeking.  Dawson et al., 2013 | Relatives struggled with the stigma attached to their relative having, or possibly having, a mental illness.  Stigma was reported to have an impact on carers recognizing and acknowledging their relative may be ill as well as how they managed the symptoms e.g. secrecy. | “I didn’t want him to think that, ya know, we were trying to tell him that he was crazy or something like that, but just that he needed to talk to someone who could probably explain to him better than I could what he was experiencing.” |
| The service users could be a deterrent in the help-seeking process. (U)  Dawson et al., 2013 | The ill relatives themselves were frequently viewed as a deterrent in the help-seeking process. In many instances, participants’ responses indicated that treatment delay may have been partly driven by the notion that the patient was “of age” and capable of (and responsible for) independent decision-making. | “I just kinda let him make his own decisions, ya know. I usually try to help him make decisions, but he says I treat him like a little boy.” |
| Carers’ informal networks contributed to clarifying mental illness and facilitating the help-seeking process. (U)  Dawson et al., 2013 | Initial detection of problems, clarification of the seriousness of problems and encouragement to seek help was often from within carers’ informal networks. Various relations who may have a role as facilitator are described within these findings including parents, siblings, children, family members, relatives, friends and religious leaders. The informal network also provides invaluable support to carers. | “I go to my friend who told me, ‘take her to the clinic.’”   “I knew something was wrong with my son. I did not know what to do, and so I asked my sister-in-law about it. She thought that my son might have a mental illness.” |
| Carers start to actively seek help when coping mechanisms become overwhelmed after ‘crisis point’. (U)  Dawson et al., 2013 | Carers start to actively seek help when coping mechanisms become overwhelmed, traditional healing methods become viewed as ineffective, or they are presented with a crisis. | “It took me quite a long time to understand that there was a mental issue there but it wasn’t until she started to become very ill. She actually had a crisis.” |
| Formal networks were either helpful or unhelpful in facilitating the help-seeking process. (U)  Dawson et al., 2013 | Relatives reported conflicting responses when seeking help from formal networks e.g. general practitioners (GPs), social workers, counsellors. Some were viewed as helpful and others unhelpful in facilitating the help-seeking process. | “So I brought him along to our local GP and told her what I felt was really wrong with him and she kind of dismissed it and said ‘no, I think he just needs to sleep, he needs some sleeping tablets and he needs to sleep’. […] She [GP] said ‘No, no give him these tablets and he’ll be fine’. And his situation got worse.”  “Our GP has been fantastic. His relationship is such that … (carer’s son) actually went to him when he was first ill. So that’s a huge bonus I think, and he referred … (carer’s son) to our local hospital and he rang them and organised for them to accept … (carer’s son) as a way of referring him on to Orygen …, because he knew that he needed that. My GP’s gives ongoing support; I suppose by him just encouraging us to keep going, because he tells us, ‘you are a good mum.’” |
| Help was not available until the ill relative seriously unwell due to system barriers. (U)  Dawson et al., 2013 | Various findings alerted to the ill relative needing to present as convincingly unwell, in crisis, at risk of harming themselves or others, or at least willing to engage in treatment in order to gain access to services. | “Consequently, treatment often began with emergency services and inpatient hospitalization, which four family members described explicitly as ‘traumatic’” |
| Help-seeking often required multiple attempts. (U)  Dawson et al., 2013 | Help-seeking was often a continuous process requiring multiple attempts on behalf of the relative and sometimes ill individual, to get appropriate help. | “One parent described trying to convince health care professionals for over one year that her son needed help for his psychiatric illness. After a number of attempts to get help, she recalled an incident where her son was found by a girl along the side of a road after being missing in the woods for 11 days. The police brought her son to the emergency department so that he could be physically stabilized. After he was physically stable the staff were about to discharge him, which greatly concerned his parents.” |
| Upon illness recognition, relatives attempted to explain the precipitators and causes of the ill individuals’ changed presentation. (U)  Dawson et al., 2013 | During the first stage of illness recognition relatives attempted to make sense of the ill individuals’ changed presentation.  After clarification that an illness was present, relatives reported trying to understand what may have triggered or caused the illness. | “I think that’s the hardest thing, you can’t! You can’t actually get a concrete answer. You know, there are just all these ideas out there, lots and lots of ideas. Nothing says, ‘well yeah, it’s definitely that’” |
| Carers expressed difficult emotions ranging from fear, sadness, helplessness, hopelessness, isolation, guilt, distress, confusion, and embarrassment in help-seeking process. (U)  Dawson et al., 2013 | The majority of findings were carers expressing difficult emotions ranging from fear, sadness, helplessness, hopelessness, and isolation. Carers expressed guilt at not recognizing signs of the illness earlier. Some expressed feeling distressed, embarrassed and confused when confronted with psychotic symptoms and behavioral changes. | “I didn’t stop feeling concerned, and I still haven’t stopped feeling concerned.”   “One family member felt ‘it is all over’. One parent wondered if her son could ever get married and have children, and another mother worried what would happen to her child when she eventually died.” |
| Relatives managed difficult behaviors and developed coping strategies before active help-seeking. (U)  Dawson et al., 2013 | Before active help-seeking had commenced, problems were becoming evident, and carers were often required to manage difficult behaviors and develop coping strategies. | “There was nothing I could really do to try to make him come together, so I kinda ignored it completely. I just forgot about it.” |
| Immersing in one’s own business acted as a protective factor for siblings in early adulthood during the prodromal phase. (U)  Dawson et al., 2013 | From the prodromal phase until the time of their sibling’s diagnosis of schizophrenia, most well siblings were embracing their independence and were immersed in other things, such as schooling and work, as the majority were in adolescence and early adulthood | “To be honest I was totally focused on my own life and didn’t even have much of a relationship with Isaac at that time (when the illness was emerging).” |

**Lever Taylor B, Billings J, Morant N, Johnson S. How do women’s partners view perinatal mental health services? A qualitative meta‐synthesis. Clinical psychology & psychotherapy. 2018;25(1):112-29.**

| **Findings, credibility rating and citations** | **Verbatim excerpts of the source reviews** | **Cited illustrative quotes** |
| --- | --- | --- |
| Partners were dismissed and left out by the serivice. (U)  Lever Taylor et al., 2018 | A key theme … related to the exclusion, marginalization, or neglect of women’s partners by the system and by healthcare professionals. | “You know, I was never included. I mean, it was like, like I wasn’t part of it. She told me that her OB/GYN said it was that [postnatal depression], but I didn’t know what that meant for her. Well, for us really. That didn’t feel good at all. “ |
| Partners reported an unmet need for information. (U)  Lever Taylor et al., 2018 | A second, related theme centred on a lack of relevant information for partners. Partners said they wanted to help but did not know how and “had no idea how to get help” . | “I needed advice on how to handle the illness and what to say. Also, information on the early signs of relapse to watch for and if it was to the point that I needed to get help.” |
| Partners felt ambivalent about involvement, wanting involvement while minimising their own needs. (U)  Lever Taylor et al., 2018 | Although partners reported feeling underinformed and neglected, there was at the same time a somewhat competing theme related to their own ambivalence about being better included and attended to. …, a majority of the studies reviewed (n = 14) also depicted partners themselves as struggling to identify their own needs, minimizing their own desires, prioritizing the woman, or expressing a reluctance to ask for or accept help or involvement. | “I think the main priority, I think, is to take care of themother first because she’s the one home with the kidsand they are the main priority too. As far as the fathers,well you know, it’s just as long as they are given at least acknowledgment.” |
| Practical barriers hindered partners from accessing support. (U)  Lever Taylor et al., 2018 | ... practical difficulties could present a barrier to partners being involved or accessing support, particularly where the woman’s care was not local. | “I had a lot of responsibility but I think because so much was going on I didn’t have the energy to seek out one person to find out more about this.” |
| Partners held mixed views on support, with unclear preferences and conflicting feedback. (U)  Lever Taylor et al., 2018 | Mirroring partners’ ambivalence around involvement and support, there was also divergence or discrepancy apparent between and even within studies about what support would be appropriate for partners. | “I don’t think [a group] would work because, I think, I wouldn’t feel comfortable in a group situation where I didn’t know the other people intimately, not intimately but enough to be able to cry and talk in front of them.”  “I did attend a couple times, but the group was so big that I found it wasn’t really intimate. It wasn’t a place where I felt super comfortable opening up.” |
| Partners experienced relief once they felt the women was getting good care. (U)  Lever Taylor et al., 2018 | ..., partners did emphasize that above all they wanted professionals to help the woman get better, … they described experiencing considerable relief themselves once they felt she was receiving appropriate treatment, … | “When she and the baby were admitted, I was able to relax a bit.”  “I was scared that the baby would be disabled by the effect of medication, but I felt relieved when I heard that the doctor agreed to have her take medicine for symptoms.” |

**Oketah NO, Hur JO, Talebloo J, Cheng CM, Nagata JM. Parents’ perspectives of anorexia nervosa treatment in adolescents: a systematic review and metasynthesis of qualitative data. Journal of eating disorders. 2023;11(1):193.**

| **Findings, credibility rating and citations** | **Verbatim excerpts of the source reviews** | **Cited illustrative quotes** |
| --- | --- | --- |
| The initial reaction at discovering their child had eating disorders was often denial and shock. (U)  Oketah et al., 2023 | The initial reaction was predominated by denial and shock at discovering their child had an ED, ... | “… I suppose really would be the initial stage of shock, we found it very hard.”  “I thought it was for attention. I thought it was a phase. I had no idea how serious.” |
| Parents’ struggle with guilt and self-blame. (U)  Oketah et al., 2023 | ... parents’ struggle with guilt and self-blame. Parents often blamed themselves for not recognising the illness earlier, not seeking help, or even believing they were ‘causing the illness’. | “I felt I should have picked up on it earlier”  “I should have been more watchful”  “did we not pay enough attention?” |
| Parents were worried and anxious during the treatment process. (E)  Oketah et al., 2023 | ...parents were worried about knowing too little about the disease and not knowing the treatment process, which some found unbearable. Anxiety was heightened by self-blame. | No quotes. |
| Parents expressed feelings of helplessness or powerlessness. (U)  Oketah et al., 2023 | ... the parents’ account of feelings of helplessness or powerlessness, ... Feelings of hopelessness may lessen the effectiveness of a parent’s role in a child’s treatment, … | “We’d failed as parents”  “I felt helpless”  “… I kind of think that nothing I do helps really” |
| As parents caring for their children, they felt isolated and stigmatised. (U)  Oketah et al., 2023 | … parents struggling with loneliness. As their responsibility for caring for their child increased, they felt further isolated, stigmatised, and cut off from their friends. Parents felt that stigma around mental illness and EDs contributed to a lack of support and increased blame from peers. | “It was just me and her…”  “It is lonely… friends were frightened to come…”  “… we’d go out for meals with people... that’s stopped because… she found it difficult” |
| The family doctors’ inability to provided required information and appropriate treatment options. (U)  Oketah et al., 2023 | ... an initial barrier in help-seeking was a perceived knowledge gap by their doctor in the area of EDs, as there was an inability to provide required information or information on treatment options. | “… the initial contact with the GP is not great…”  “They need to understand it’s not just about the weight…” |
| Parents reported different experiences with diverse treatment modalities. (U)  Oketah et al., 2023 | Overall, FBT was seen as positive by parents, ... …, MFT was viewed positively by some parents  Other studies highlighted negative aspects of experiences with FBT/MFT, ...  Home treatment … Parents experienced containment, provision of hope, an opportunity to find strength, and a reduction in the burden of care. On the negative side, parents reported fear and anxiety about the future   Inpatient treatment … parents’ experience of inpatient treatment as inappropriate as it was in a general ward. … Feelings of guilt were exacerbated by not being with their child constantly …  Compulsory treatment/control they were conflicted between acknowledging that compulsory treatment was justified and wanting their child to be treated with dignity (their consent), … | Positive:  On FBT:  “It developed an openness that hadn’t been there before”  “Attending the parenting courses was very helpful…” On home therapy: “I felt energized…”  On MFT: “… if it came from my child, I would not have understood. I understood better through the words of another young person”   Negative:  On FBT: “… they didn’t appear to be doing very much or to be talking about the whys and wherefores”   On in-patient treatment: “How thin must one become to receive treatment? Actually, she lost weight just to meet their BMI criteria”  Compulsory treatment/control “… I think if their life is at risk… someone has to make the decision…” |
| Carers valued professionals’ empathy, competence, availability, and respectful attitudes. (U)  Oketah et al., 2023 | Availability, competence, and positive attitudes like empathy, sensitivity, respectfulness, friendliness, and trustworthiness were especially highlighted in six studies and were deeply appreciated by parents. | “… He (psychiatrist) just kind of saved us in a way”   “She (therapist) would calm us down…”   “a parent referring to their psychiatrist as ‘some kind of saviour’” |
| Parents reported feeling frustrated for negative attributes of health professionals. (U)  Oketah et al., 2023 | ... a lack of feedback from clinicians, which made them unclear about their child’s prognosis during therapy.  ... Parents felt that doctors, nutritionists, social workers, and other members of the medical community who were unfamiliar with AN negatively impacted the diagnosis and treatment of their child. … Parents reported negative attitudes where they felt like they were being blamed or judged ... | “…there wasn’t any other feedback other than ‘oh well that’s difficult’…”  “I felt very judged… at a time in my life when the core thing I needed was support” |
| Parents observed positive changes during recovery. (U)  Oketah et al., 2023 | Six studies reported parents observing an overall positive outlook with recovery, personality change, better engagement with the family, and increased confidence with recovery. | “… she talked about wanting to get a job…”  “… She is more flexible and open about changes and new activities” |
| Parents experience uncertainty about the future. (U)  Oketah et al., 2023 | ... there was a lot of uncertainty during the recovery period and difficulty in predicting the future while fearing that AN ‘might return.’ | “It’s all unknown”  “What on earth is going to happen next with this..?” |
| Parents feel deep anguish and struggle with support during relapse. (U)  Oketah et al., 2023 | ... Parents experienced substantial anguish when relapse occurred, ... It was agonising watching their child deteriorate after they had made some progress, and parents struggled to find support during this time from professionals. ... Some even found it depressing. | “… taking one step forward and then three back…”  “… to accept relapses was difficult. To accept that it didn’t go according to the pace I had hoped…” |

**Reupert A, Tchernegovski P, Chen L, Huddle M. Experiences of family members when a parent is hospitalized for their mental illness: a qualitative systematic review. BMC psychiatry. 2023;23(1):56.**

| **Findings, credibility rating and citations** | **Verbatim excerpts of the source reviews** | **Cited illustrative quotes** |
| --- | --- | --- |
| Families experienced pressures and a change in responsibilities during the whole process of the parents’ care. (U)  Reupert et al., 2023 | Seeking admission for the parent was challenging for families. … When parents were admitted, there were changes to children’s responsibilities. Discharge was another stressful time | “…they describe it as a battle to get help”  “We said she couldn’t just be discharged; she wasn’t capable of managing herself, but in fact that’s what they intended to do!” |
| Family members were disappointed at the lack of information or guidance. (U)  Reupert et al., 2023 | ... parents, children, and other relatives were all disappointed at the lack of information or guidance about children visiting their parents in the hospital. | “You shouldn’t be left in the dark because you’re a kid and maybe they don’t give us credit… you can handle it. It is much more scary not knowing” |
| Children felt lethargic, depressed, withdrawn, or anxious when their parent got hospitalised and wanted connection. (U)  Reupert et al., 2023 | Children experienced a range of emotions when their parents were hospitalized. Some children became lethargic, depressed and withdrawn while others described experiences of anxiety. … ... children of various ages described a need for connection and “someone to talk things through with”. | “I wished that I could have seen a social worker or a psychologist, someone who could have asked me about my reactions…”. |

**Rodell S, Parry S. Family members’ experiences of seeking help for a young person with symptoms associated with the psychosis spectrum: A narrative review and synthesis. Clinical child psychology and psychiatry. 2024;29(3):897-912.**

| **Findings, credibility rating and citations** | **Verbatim excerpts of the source reviews** | **Cited illustrative quotes** |
| --- | --- | --- |
| Family members’ own stigmatised attitudes were initial barriers to help-seeking. (U)  Rodell & Parry, 2024 | ... family members spoke of how their own stigmatised attitudes toward psychosis acted as initial barriers to help-seeking.  Stigma was echoed in narratives suggesting their loved one was hostile toward their efforts to seek help on their behalf, with self-stigma acting as a further barrier to accessing timely care. | “I always thought mental illness was something you didn’t talk about because there was so much shame” |
| Limited mental health literacy contributed to uncertainty surrounding how and where to access support. (U)  Rodell & Parry, 2024 | A sense of ‘not knowing’ surrounding psychosis and USEs was frequently highlighted... Furthermore, limited mental health literacy contributed to uncertainty surrounding how and where to access support, delaying help-seeking further. | “I didn’t know anything about it” |
| Family members’ distrust in services lead to reluctance to help-seeking. (U)  Rodell & Parry, 2024 | … negative past experiences of inpatient mental health services contributed to lasting distrust and reluctance to seek help from similar services on subsequent occasions. | “GPs will not be very informed, will not refer you to the right services”. |
| Reaching a crisis point appeared to provoke dramatic increases in help-seeking. (U)  Rodell & Parry, 2024 | Reaching a crisis point appeared to provoke dramatic increases in help-seeking, ..., the absence of crisis may act as a barrier to help-seeking. | “My putting him in the hospital was my cry for help too”  “When she made a suicide attempt…it was like she just has to get help…I didn’t care about labels” |
| Sourcing support involved multiple agencies and significant wait times. (U)  Rodell & Parry, 2024 | Sourcing support involved multiple agencies and significant wait times. | “CAMHS is a long wait and hard to get through to”. |
| There was a significant gap in tailored services to young people. (U)  Rodell & Parry, 2024 | A significant gap in services was observed for young people: ... Being unable to access targeted support raised concerns surrounding whether care received was age-appropriate ... | “no way he should ever have been in a place like that when he was 16…but they had nowhere else to put him”  “zero services are available for someone experiencing voices so young” |
| A lack of continuity in services. (U)  Rodell & Parry, 2024 | A lack of continuity in services was voiced in five studies ... This lack of continuity was also highlighted between services and ... especially prominent following discharge from inpatient services, … | “We kept on getting sick of telling our same story to different people”. |
| Families felt being excluded from provisions of care. (U)  Rodell & Parry, 2024 | Within family narratives, feeling excluded from provisions of care was expressed ... Although the importance of confidentiality was recognised, frustration was expressed nonetheless ... | “I feel like I’m being left out”.   “There’s issues of patient confidentiality…but then there’s the issue of parents out there, who have no clue as to what is happening with their child”. |
| Feelings of helplessness, fear, failure, guilt, anxiety, and disempowerment were raised. (U)  Rodell & Parry, 2024 | …, experiences of feeling helpless and scared were raised … many family members’ accounts highlighted feelings of failure and guilt … One source of anxiety amongst family members was caused by a lack of confidence and self-belief to help their loved one, indicating feelings of disempowerment. … A strong emotional impact on family members was reported across all of the reviewed literature. | “It was really scary…there was nothing anyone could do”. |
| Family members experience ongoing concern and guarded hope for recovery. (U)  Rodell & Parry, 2024 | Even after accessing support from services, continued concern for their loved one and future prospects was prominent, ... There also seemed to be a tension between wanting to be hopeful, but describing ‘guarded hope’, … | “I probably felt sorry for myself, thinking…what are we gonna do?”   “I want to be optimistic but I feel maybe she can only be helped so much and then this will happen again. I fear she will backslide. My only hope is that she will be happy and enjoy life”. |
| Family members experienced isolation in their caring roles. (NS)  Rodell & Parry, 2024 | While family members rarely voiced feeling isolated explicitly, isolation ran through their stories. | No quotes. |
| Specialist services often led to physical and emotional distance between carers and service users. (U)  Rodell & Parry, 2024 | ... escalation of symptoms frequently created a physical and emotional distance as primary care was handed over to specialist services, leaving a sense of loss.... | “one woman prayed to understand why her son had become like a stranger”. |
| Some carers felt themselves grow from the caring experience. (NS)  Rodell & Parry, 2024 | ... feeling they had “grown from their experiences” through challenging their own negative pre-conceptions … | “it can happen to anyone”. |

**Stuart R, Akther SF, Machin K, Persaud K, Simpson A, Johnson S, et al. Carers’ experiences of involuntary admission under mental health legislation: systematic review and qualitative meta-synthesis. BJPsych Open. 2020;6(2):e19.**

| **Findings, credibility rating and citations** | **Verbatim excerpts of the source reviews** | **Cited illustrative quotes** |
| --- | --- | --- |
| Conflicting emotions were reported in response to detention, with distress being most common, alongside some positive expressions of relief and gratitude. (U)  Stuart et al., 2020 | Distress was the emotion most frequently described but carers also reported a range of other, often conflicting, emotions in response to the detention of the person for whom they cared. …Positive emotions included feelings of relief, hope and gratitude. ... Feelings of distress were variously attributed ... | “Participants’ accounts of hospitalisation framed it overwhelmingly as an appropriate intervention that brought relief and respite.” |
| Cares needed support throughout the process, but the lack of adequate support for carers was prominent. (U)  Stuart et al., 2020 | The lack of adequate support for carers of patients being assessed or detained under mental health legislation was a prominent theme across all papers. … Carers reported that they needed support not only in the period prior to assessment and detention but also during this time and afterwards, including to understand what had happened and their role in it. | “Relatives reported not knowing what to do, where to get information and help from, and expressed that this was an extremely stressful and difficult time for them… at this early point, you were “on your own”, struggling to persuade others for assistance.” |
| The extent of carers’ involvement in decision-making and the provision of care was a key influence on carers’ experiences under mental health legislation, but their involvement was often limited. (U)  Stuart et al., 2020 | The extent to which carers felt involved in decision-making and the provision of care was a key influence on carers’ experiences of supporting patients detained under mental health legislation. ...Yet, information was often lacking. ... After detention it was a source of frustration to many carers to not be involved in discharge planning ... | “Parents wanted to be treated as a resource and described recognizing patterns of emotions and behaviours. They hoped that by sharing these with health professionals, they would make better and more informed treatment decisions.” |
| Detention under mental health legislation could lead to the breakdown of relationships between the patients and carers. (U)*   Stuart et al., 2020 | The detention of a patient could lead to the breakdown of the relationship between the patient and carer. | “[Carers] describe how they and their sick family member “lose each other” due to long hospitalisations. This breaking of family bonds is particularly problematic if the family is responsible for practical and emotional support following hospitalisation.” |
| Many carers described their relationships with healthcare professionals as unsatisfactory. (U)*  Stuart et al., 2020 | Across several papers many... carers described their relationships with healthcare professionals, ... as having been unsatisfactory. | “‘It’s a terrible battle’ … describes the relationships between parent caregivers and mental health professionals before and after admission …. The majority of parents described a strained and invalidating relationship with professionals.” |

**Sugiura K, Pertega E, Holmberg C. Experiences of involuntary psychiatric admission decision-making: a systematic review and meta-synthesis of the perspectives of service users, informal carers, and professionals. International journal of law and psychiatry. 2020;73:101645.**

| **Findings, credibility rating and citations** | **Verbatim excerpts of the source reviews** | **Cited illustrative quotes** |
| --- | --- | --- |
| Families felt that they were protecting the family member by admitting them. (E)  Sugiura et al., 2020 | Some families felt that they were protecting the family member by signing and admitting them to the hospital. | “I felt responsible for my son, so I thought it was OK for me to sign the form.” |
| Families felt that signing the involuntary admission would ruin their relationship with the servuce users. (U)  Sugiura et al., 2020 | Some families found it hard to sign a form to admit their family member because they felt as if they were putting the person away. They also worried that becoming the applicant could damage their relationship with the service user. | “The GP wanted me to sign, and said that he could get an ASW (Approved Social Worker) as an alternative, but that this would take hours, I reluctantly agreed, just to cut the pain. … I signed for my son but never again. … I felt that I had ‘put him away.’ It is much better that this should be done by someone like an ASW who can then take the blame.” |
| Families found it difficult to get support and the services were not proactive. (U)  Sugiura et al., 2020 | Families found it difficult to navigate the system to get support, they were frustrated with a delay in support, and they felt services were reactive rather than proactive. | “The mental health system was good at reaction, but hopeless at pro-action, and unless there was some serious incident, like the self-harming, or the time when she wouldn’t get out of bed, to which they could respond with sort of blue lights flashing and so on, they were absolutely useless.” |
| Families were not ready to make the involuntary admission decision as they lacked information about the process and its legislation. (U)  Sugiura et al., 2020 | Families felt that they lacked information about the involuntary admission process and its legislation, and they felt that they were not ready to make the involuntary admission decision. | “In my experience it is not explained to the carer. Had I known the details I would have thought twice about whether to admit my relative.” |
| Families struggled to be recognized by professionals in care. (U)  Sugiura et al., 2020 | Families struggled to be recognized by professionals in care and often felt that they were undervalued. | “Until we had the compulsory order (and being Named Person) you really were largely on your own and the information didn’t come to you, you had to seek it out and it was hard to come by ... Since we had the compulsory order, we have found a lot more about what is available, what we’re entitled to and have had a lot more help.” |
| Families felt that the services were degrading for the service users. (U)  Sugiura et al., 2020 | Families described the experience of using the mental health system as harmful and degrading for family members | “The mental health system can be so degrading, ending up with very low self-esteem—compulsion is part of this—my brother would refer to it as the way the world had harmed him.” |
| Families found family group conferencing (FGC) helpful in promoting understanding and avoiding coercive measures. (U)  Sugiura et al., 2020 | Families found FGC to be helpful in finding mutual understanding with neighbors and avoiding coercive measures including involuntary admission. | “Arie’s behavior made the situation after the conference initially worse, but in the longer term it appeared to have a positive effect on Arie as well on the residents: He has now realized that the residents want him to stay, but that they do not accept all of his behavior. I think that he now feels safer and comfortable with these new boundaries.” |

**Vestphal TK, Gildberg FA, Jørgensen R, Rowaert S, Tingleff EB. Experiences of family caregivers in forensic mental health care—A qualitative evidence synthesis. Journal of psychiatric and mental health nursing. 2023;30(4):663-78.**

| **Findings, credibility rating and citations** | **Verbatim excerpts of the source reviews** | **Cited illustrative quotes** |
| --- | --- | --- |
| Caregivers reported anxiety, fear, hypervigilance and exhaustion. (U)  Vestphal et al., 2023 | The majority of caregivers and families had been exposed to mental and physical violence by the service user over many years … Because of their experiences, the caregivers reported anxiety, fear, hypervigilance and exhaustion … | “I am afraid of his bad conduct, that if he comes back, he will raise trouble. He also hit me with his fist and two of my teeth fell out” |
| Caregivers reported a sense of responsibility for the person they cared for. (U)  Vestphal et al., 2023 | This theme represented characteristics of caregivers’ sense of responsibility and burden in relation to the service user. | “We are both ill and neither of us (parents) know the minute we are not going to be here, and there’s going to be a big change because we can’t see anybody going there and looking after her”. |
| Caregivers felt being excluded from the service users’ care and treatment. (U)  Vestphal et al., 2023 | Caregivers’ experiences of feeling excluded from the service user’s care and treatment characterized this theme. | “They work in their towers, they could not care less about the family…the doctors don’t care about us, they don’t talk to us, they don’t ask us anything…”. |
| Caregivers possessed comprehensive knowledge of the service users. (U)  Vestphal et al., 2023 | Caregivers experienced and perceived that their comprehensive knowledge of the service user was a potential facilitator in their involvement in care and treatment. However, when the caregiver felt ignored, despite such knowledge, and excluded by HCPs from care and treatment, this could lead to feelings of anger, disempowerment and distrust. | “I have warned, and warned and warned the hospital, I warned his (staff) carer, the social worker, how bad things were. I almost feel like (son) had to get as bad as he did, before I got help for him”. |
| Most caregivers were very persistent in caring for the service user. (U)  Vestphal et al., 2023 | This theme was characterized by most caregivers being very persistent in taking care of the service user, to a point where they lived their lives with an almost constant focus on their ill family member. | “…He used 80% of my strength. My other son used 20%…”. |
| Caregivers advocated for the protection and rights of the service user as they felt the need. (U)  Vestphal et al., 2023 | … some caregivers were of the perception that the service user was unfairly treated and/or victimized by the healthcare system or by HCPs, which could create the foundation for, what may be described as caregivers advocating for the protection and rights of the service user. | “The doctor says, ‘to her’ ‘Ach you’ll be alright, I’ll give you a couple of pain killers, go away on home there, you’ll be okay’. I say, I want you to repeat what you just said in front of a witness, because when she goes outa here and harms herself or somebody else, I’m taking you to court”. |

**D. Findings contributing to each category (Tables 1-20)**

This appendix contains 20 tables, each presenting all findings contributing to each specific category, alongside verbatim excerpts from the source reviews (i.e. text copied directly from the included reviews without modification, representing the original descriptions of findings reported by the review authors). The order of the categories follows the same sequence and structure as presented in **Table 2** of the main text, which outlines the organisation of the synthesised findings and supporting categories.

Credibility rating:

Unequivocal (U): supported by clear, direct quotes or observations.

Equivocal (E): logically inferred but less directly supported.

Unsupported (US): findings not supported.

* The finding was derived from a theme that also gave rise to another finding in the original review. (Findings typically reflected a single theme or subtheme, though in a few cases one theme yielded two findings. These instances were documented and marked with an asterisk (*) to maintain transparency and avoid overrepresentation in the synthesis.)

**Synthesised finding 1: Emotional complexity in the caregiving journey**

**Category: High emotional distress during the build-up to help-seeking and accessing services**

(Informal carers of people with mental difficulties underwent intense distress before accessing services.)

| **Findings, credibility rating and citations** | **Verbatim excerpts of the source reviews** |
| --- | --- |
| The build up to hospitalisation was distressing, overwhelming, powerless, and frustrated. (U)  AbouSeif et al., 2022 | The build up to hospitalisation was described as distressing and overwhelming, ... Carers report feeling powerlessness and frustration that help was seemingly only made available once their loved one had deteriorated to a point where hospitalisation was inevitable. |
| Carers experienced increased stress, shame, stigma, guilt, resentment, grief, and worry during the process of accessing respite care. (E)  Almoaber et al., 2024 | ... the subjective burden (shame, stigma, guilt, resentment, grief, worry) remained unaffected. Regarding this aspect, carers reported experiencing an increased stress level. |
| Carers reported fear, high stress, hypervigilance in the build-up to a service user’s crisis, and stress and frustration in the build-up to services. (U)*  Bartl et al., 2024 | Carers reported fear about service users’ symptoms and behaviour prior to detention, high stress and hypervigilance in the build-up to a service user’s crisis; and frustration when health professionals ignored or ‘failed to grasp the gravity of the service user’s illness’. |
| Upon noticing changes in loved ones, carers experience a sense of ‘not knowing’ upon noticing changes in loved ones characterized by a sense of confusion, frustration, and desperation. (U)*  Cairns et al., 2015 | Upon noticing changes in their loved ones, family members experienced a sense of ‘not knowing’ and attempted to make sense, understand the experience and seek answers to difficult questions. This experience was characterized by a sense of desperation while trying to meet the needs of their loved ones. |
| Seeking help for FEP on behalf of a loved one led to high levels of distress and sometimes a sense of trauma. (U)   Cairns et al., 2015 | The experience of seeking help for FEP on behalf of a loved one had a significant emotional impact upon family members, including high levels of distress and sometimes a sense of trauma. The help-seeking experience also impacted upon relationships, with both others and the self, often negatively, but at times (often later in the process) positively. |
| Carers expressed difficult emotions ranging from fear, sadness, helplessness, hopelessness, isolation, guilt, distress, confusion, and embarrassment in help-seeking process. (U)  Dawson et al., 2013 | The majority of findings were carers expressing difficult emotions ranging from fear, sadness, helplessness, hopelessness, and isolation. Carers expressed guilt at not recognizing signs of the illness earlier. Some expressed feeling distressed, embarrassed and confused when confronted with psychotic symptoms and behavioral changes. |
| Feelings of helplessness, fear, failure, guilt, anxiety, and disempowerment were raised. (U)  Rodell & Parry, 2024 | …, experiences of feeling helpless and scared were raised … many family members’ accounts highlighted feelings of failure and guilt … One source of anxiety amongst family members was caused by a lack of confidence and self-belief to help their loved one, indicating feelings of disempowerment. … A strong emotional impact on family members was reported across all of the reviewed literature. |
| Caregivers reported anxiety, fear, hypervigilance and exhaustion. (U)  Vestphal et al., 2023 | The majority of caregivers and families had been exposed to mental and physical violence by the service user over many years … Because of their experiences, the caregivers reported anxiety, fear, hypervigilance and exhaustion … |

**Category: Mixed and complicated emotional responses to service involvement**

(Informal carers of people with mental difficulties underwent mixed and conflicting feelings during service involvement, reporting both positive and negative feelings.)

| **Findings, citations, and credibility rating** | **Verbatim excerpts of the source reviews** |
| --- | --- |
| Carers reported a mixture of emotions such as relief, guilt, fear, and hope upon their loved one’s hospitalisation. (U)  AbouSeif et al., 2022 | Once their loved one was hospitalised, carers reported experiencing a mixture of conflicting emotions such as relief, guilt, fear, and hope. |
| Most informal carers experienced relief during their use of respite care. (U)  Almoaber et al., 2024 | most of the informal carers experienced relief from burden during their use of respite care … services that offer respite care provide the necessary security for informal carers to place their family members in care. Furthermore, this peace of mind enables carers to perceive the respite experience positively. |
| Carers felt bad and distressing assessment and admission. (U)*  Bartl et al., 2024 | Carers felt bad about initiating coercive measures and found assessment distressing and admission traumatizing. |
| Some carers felt some relief upon their loved one’s admission. (U)  Bartl et al., 2024 | Following admission, some carers felt some relief because they were reassured that the risk of harm to self or others had been averted, and the service user was now safe and in receipt of care. |
| Detention caused emotional strain and reduced carers’ psychological wellbeing. (U)  Bartl et al., 2024 | Personal uncertainty, disagreements with other family members about the right course of action, and differences between what the person they cared for wanted and what was in their best interests, all took a toll on carer wellbeing. |
| Partners experienced relief once they felt the women was getting good care. (U)  Lever Taylor et al., 2018 | ..., partners did emphasize that above all they wanted professionals to help the woman get better, … they described experiencing considerable relief themselves once they felt she was receiving appropriate treatment, … |
| Parents’ struggle with guilt and self-blame. (U)  Oketah et al., 2023 | ... parents’ struggle with guilt and self-blame. Parents often blamed themselves for not recognising the illness earlier, not seeking help, or even believing they were ‘causing the illness’. |
| Parents were worried and anxious during the treatment process. (E)  Oketah et al., 2023 | ...parents were worried about knowing too little about the disease and not knowing the treatment process, which some found unbearable. Anxiety was heightened by self-blame. |
| Parents expressed feelings of helplessness or powerlessness. (U)  Oketah et al., 2023 | ... the parents’ account of feelings of helplessness or powerlessness, ... Feelings of hopelessness may lessen the effectiveness of a parent’s role in a child’s treatment, … |
| As parents caring for their children, they felt isolated and stigmatised. (U)  Oketah et al., 2023 | parents struggling with loneliness. As their responsibility for caring for their child increased, they felt further isolated, stigmatised, and cut off from their friends. Parents felt that stigma around mental illness and EDs contributed to a lack of support and increased blame from peers. |
| Families experienced pressures and a change in responsibilities during the whole process of the parents’ care. (U)  Reupert et al., 2023 | Seeking admission for the parent was challenging for families. … When parents were admitted, there were changes to children’s responsibilities. Discharge was another stressful time |
| Children felt lethargic, depressed, withdrawn, or anxious when their parent got hospitalised and wanted connection. (U)  Reupert et al., 2023 | Children experienced a range of emotions when their parents were hospitalized. Some children became lethargic, depressed and withdrawn while others described experiences of anxiety. … ... children of various ages described a need for connection and “someone to talk things through with”. |
| Conflicting emotions were reported in response to detention, with distress being most common, alongside some positive expressions of relief and gratitude. (U)  Stuart et al., 2020 | Distress was the emotion most frequently described but carers also reported a range of other, often conflicting, emotions in response to the detention of the person for whom they cared. …Positive emotions included feelings of relief, hope and gratitude. ... Feelings of distress were variously attributed ... |

**Category: Uncertainty and guarded hope about the future**

(Informal carers of people with mental difficulties expressed ongoing uncertainty and guarded hope about the future.)

| **Findings, citations, and credibility rating** | **Verbatim excerpts of the source reviews** |
| --- | --- |
| Parents experience uncertainty about the future. (U)  Oketah et al., 2023 | ... there was a lot of uncertainty during the recovery period and difficulty in predicting the future while fearing that AN ‘might return.’ |
| Parents feel deep anguish and struggle with support during relapse. (U)  Oketah et al., 2023 | ... Parents experienced substantial anguish when relapse occurred, ... It was agonising watching their child deteriorate after they had made some progress, and parents struggled to find support during this time from professionals. ... Some even found it depressing. |
| Family members experience ongoing concern and guarded hope for recovery. (U)  Rodell & Parry, 2024 | Even after accessing support from services, continued concern for their loved one and future prospects was prominent, ... There also seemed to be a tension between wanting to be hopeful, but describing ‘guarded hope’, … |

**Synthesised finding 2:** **Recognition and coping with their loved ones’ mental health difficulties**

**Category: Early recognition, reactions, and management**

(Carers were often among the first to notice changes in their loved ones. Initial denial, attributions to non-mental health causes, and interpretations based on their own knowledge and belief system influence their perceptions and corresponding management.)

| **Findings, citations, and credibility rating** | **Verbatim excerpts of the source reviews** |
| --- | --- |
| Upon noticing changes in their loved ones, informal carers struggle to make sense of the situations and to cope. (U)*  Cairns et al., 2015 | Upon noticing changes in their loved ones, family members experienced a sense of ‘not knowing’ and attempted to make sense, understand the experience and seek answers to difficult questions. This experience was characterized by a sense of desperation while trying to meet the needs of their loved ones. |
| Difficulties were gradually noticed by relatives. (U)  Dawson et al., 2013 | Various mood and behavioral changes, as well as general functional decline, alerted relatives to there being something “wrong” with the individual developing a psychotic illness. |
| Early symptoms are commonly misatributed to alternative explanations, which influence the type and timing of help-seeking. (U)  Dawson et al., 2013 | The early phase and symptoms of psychotic illnesses are commonly attributed to factors other than mental illnesses … The misattribution of symptoms impacts upon the type and timing of help-seeking … |
| Carers’ knowledge and belief influence their perceptions and the type of help that is sought. (U)  Dawson et al., 2013 | After a problem has been acknowledged, carers attempt to understand the problems within their own belief system. Many were unable to make sense of the presenting difficulties and were unsure of how to respond. |
| Upon illness recognition, relatives attempted to explain the precipitators and causes of the ill individuals’ changed presentation. (U)  Dawson et al., 2013 | During the first stage of illness recognition relatives attempted to make sense of the ill individuals’ changed presentation. After clarification that an illness was present, relatives reported trying to understand what may have triggered or caused the illness. |
| Relatives managed difficult behaviors and developed coping strategies before active help-seeking. (U)  Dawson et al., 2013 | Before active help-seeking had commenced, problems were becoming evident, and carers were often required to manage difficult behaviors and develop coping strategies. |
| The initial reaction at discovering their child had eating disorders was often denial and shock. (U)  Oketah et al., 2023 | The initial reaction was predominated by denial and shock at discovering their child had an ED, ... |

**Category: Boosted help-seeking after reaching “crisis point”**

(Help-seeking to external and professional sources was often prompted by a “crisis point,” where carers’ coping capacity was overwhelmed.)

| **Findings, citations, and credibility rating** | **Verbatim excerpts of the source reviews** |
| --- | --- |
| Personal coping resources became overwhelmed at ‘crisis point’ and led to a sharp increase in help-seeking behaviour. (U)  Cairns et al., 2015 | The reaching of crisis point within the help seeking experience was related to both limited knowledge and access to support, as well as families drawing upon personal coping resources before becoming overwhelmed. Crisis point was associated with a sharp increase in help-seeking behaviour by family members, a sense of relief at support being accessed and also concerns regarding that available. |
| Carers start to actively seek help when coping mechanisms become overwhelmed after the “crisis point”. (U)  Dawson et al., 2013 | Carers start to actively seek help when coping mechanisms become overwhelmed, traditional healing methods become viewed as ineffective, or they are presented with a crisis. |
| Reaching a crisis point appeared to provoke dramatic increases in help-seeking. (U)  Rodell & Parry, 2024 | Reaching a crisis point appeared to provoke dramatic increases in help-seeking, ..., the absence of crisis may act as a barrier to help-seeking. |

**Synthesised finding 3: Multiple barriers for carers in accessing mental health support**

**Category: Systematic barriers to accessing appropriate mental health support**

(Carers frequently encountered systemic inadequacies when attempting to access mental health support for their relatives, including a lack of appropriate and continuous services, restrictive entry criteria, and inadequate community care and pathways.)

| **Findings, citations, and credibility rating** | **Verbatim excerpts of the source reviews** |
| --- | --- |
| The availability of respite care services is limited. (U)  Almoaber et al., 2024 | Carers described the availability of respite care services, in general, as being limited, … |
| Entering respite care was restricted by specific criteria, which negatively impacted access. (E)  Almoaber et al., 2024 | ... entering respite care was restricted by the use of specific criteria such as the patient’s health condition, diagnosis, geographical location, age, length of the caring role and ethnicity. All these restrictions had the potential to negatively impact access to respite care due to a lack of flexibility for carers to meet the demands that respite service providers levied .... |
| Community services and pathways to care were inadequate. (U)  Bartl et al., 2024 | Several studies describe insufficient community services ... dismissed carer concerns until the service user was acutely unwell. … Some carers reported that pathways to care were inadequate. |
| Help was not available until the ill relative seriously unwell due to system barriers. (U)  Dawson et al., 2013 | Various findings alerted to the ill relative needing to present as convincingly unwell, in crisis, at risk of harming themselves or others, or at least willing to engage in treatment in order to gain access to services. Staff shortages, poor communication, lack of free or affordable services, difficulties with insurance, inflexibility and poor scheduling of appointments were additional barriers identified by carers. |
| There was a significant gap in tailored services to young people. (U)  Rodell & Parry, 2024 | A significant gap in services was observed for young people: ... Being unable to access targeted support raised concerns surrounding whether care received was age-appropriate ... |
| A lack of continuity in services. (U)  Rodell & Parry, 2024 | A lack of continuity in services was voiced in five studies ... This lack of continuity was also highlighted between services and ... especially prominent following discharge from inpatient services, … |

**Category: The uneasy and continuous process of sourcing services**

(Sourcing mental health services was an uneasy and repeated process, which reflected procedural barriers to service access.)

| **Findings, citations, and credibility rating** | **Verbatim excerpts of the source reviews** |
| --- | --- |
| Help-seeking often required multiple attempts. (U)  Dawson et al., 2013 | Help-seeking was often a continuous process requiring multiple attempts on behalf of the relative and sometimes ill individual, to get appropriate help. |
| Sourcing support involved multiple agencies and significant wait times. (U)  Rodell & Parry, 2024 | Sourcing support involved multiple agencies and significant wait times. |
| Families found it difficult to get support and the services were not proactive. (U)  Sugiura et al., 2020 | Families found it difficult to navigate the system to get support, they were frustrated with a delay in support, and they felt services were reactive rather than proactive. |

**Category: Lack of information and knowledge**

(Carers’ lack of information and knowledge of mental health services also created confusion and hindered timely, informed decision-making.)

| **Findings, citations, and credibility rating** | **Verbatim excerpts of the source reviews** |
| --- | --- |
| Carers’ lack of knowledge and understanding of respite care is a barrier to utilising the services. (E)  Almoaber et al., 2024 | Carers consider a lack of knowledge and understanding of respite care a common barrier to utilising respite services … |
| Carers reported a lack of information and guidance from health services during help seeking. (U)  Bartl et al., 2024 | Carers reported that there was a lack of information and guidance from health services as they managed a family member with deteriorating health and sought help. |
| Lack of knowledge of treatment and service options delayed help-seeking. (U)  Dawson et al., 2013 | Carers reported they were unaware of where to access help and how to negotiate access **…** |
| Limited mental health literacy contributed to uncertainty surrounding how and where to access support. (U)  Rodell & Parry, 2024 | A sense of ‘not knowing’ surrounding psychosis and USEs was frequently highlighted... Furthermore, limited mental health literacy contributed to uncertainty surrounding how and where to access support, delaying help-seeking further. |
| Families were not ready to make the involuntary admission decision as they lacked information about the process and its legislation. (U)  Sugiura et al., 2020 | Families felt that they lacked information about the involuntary admission process and its legislation, and they felt that they were not ready to make the involuntary admission decision. |

**Category: Complex personal and relational barriers**

(Carers, service users, carers’ informal networks, and healthcare professionals may all potentially hinder help-seeking and access to services.)

| **Findings, citations, and credibility rating** | **Verbatim excerpts of the source reviews** |
| --- | --- |
| The reluctance of patients with mental health difficulties to services is a significant barrier to accessing respite services. (E)  Almoaber et al., 2024 | The reluctance on the part of patients with MI towards using respite care is a significant barrier to accessing respite services. 74% of their participating carers indicated that people with MI had rejected respite opportunities. |
| Carers’ fear and unwillingness were barriers to help-seeking and accessing services. (U)  Bartl et al., 2024 | Carers had difficulties arising from their relatives’ subsequent fear and unwillingness to seek help and engage with services, withdrawing and keeping their distance from carers; being guarded and masking their symptoms during assessments. |
| Interactions with informal networks, professionals, and other families mediated the help-seeking process, either facilitating it or hindering it. (U)  Cairns et al., 2015 | Interactions had a mediating effect upon attempts to seek help, ways in which this was approached and the impact upon the family member. .... Help seeking was facilitated where these interactions identified the need for help and involved clear communication and support from professionals, whereas interactions had a negative influence upon the process where others’ attempts to normalize subsequently minimized the significance, and/or became dismissing of difficulties. |
| Relatives’ informal networks and the individuals’ wider context did not promote help-seeking.  Dawson et al., 2013 | ...the carers’ informal contacts either actively discouraged contact with mental health services or were noted as not advocating for or facilitating help-seeking. Informal networks included family members, relatives and friends as well as people in the individuals’ wider context including employers, educators and religious bodies. |
| Stigma associated with having a mental illness and of mental health services and treatment delayed help-seeking.  Dawson et al., 2013 | Relatives struggled with the stigma attached to their relative having, or possibly having, a mental illness. Stigma was reported to have an impact on carers recognizing and acknowledging their relative may be ill as well as how they managed the symptoms e.g. secrecy. |
| The service users could be a deterrent in the help-seeking process. (U)  Dawson et al., 2013 | The ill relatives themselves were frequently viewed as a deterrent in the help-seeking process. … In many instances, participants’ responses indicated that treatment delay may have been partly driven by the notion that the patient was “of age” and capable of (and responsible for) independent decision-making. |
| Carers’ informal networks contributed to clarifying mental illness and facilitating the help-seeking process. (U)  Dawson et al., 2013 | Initial detection of problems, clarification of the seriousness of problems and encouragement to seek help was often from within carers’ informal networks. Various relations who may have a role as facilitator are described within these findings including parents, siblings, children, family members, relatives, friends and religious leaders. The informal network also provides invaluable support to carers. |
| Formal networks were either helpful or unhelpful in facilitating the help-seeking process. (U)  Dawson et al., 2013 | Relatives reported conflicting responses when seeking help from formal networks e.g. general practitioners (GPs), social workers, counsellors. Some were viewed as helpful and others unhelpful in facilitating the help-seeking process. |
| The family doctors’ inability to provided required information and appropriate treatment options. (U)  Oketah et al., 2023 | ... an initial barrier in help-seeking was a perceived knowledge gap by their doctor in the area of EDs, as there was an inability to provide required information or information on treatment options. ... |
| Family members’ own stigmatised attitudes were initial barriers to help-seeking. (U)  Rodell & Parry, 2024 | ... family members spoke of how their own stigmatised attitudes toward psychosis acted as initial barriers to help-seeking.  Stigma was echoed in narratives suggesting their loved one was hostile toward their efforts to seek help on their behalf, with self-stigma acting as a further barrier to accessing timely care. |
| Family members’ distrust in services lead to reluctance to help-seeking. (U)  Rodell & Parry, 2024 | … negative past experiences of inpatient mental health services contributed to lasting distrust and reluctance to seek help from similar services on subsequent occasions. |

**Synthesised finding 4: Persistent responsibilities and multiple roles in supporting service users**

**Category: Experiential knowledge and potential to help**

(Carers had strong potential to facilitate treatment with their in-depth knowledge of the service users.)

| **Findings, citations, and credibility rating** | **Verbatim excerpts of the source reviews** |
| --- | --- |
| Carers’ familiarity with the service users gave them expertise. (U)  Bartl et al., 2024 | Carers’ familiarity with the relative they care for, having been with them when they were well and during previous episodes of illness, and knowing the circumstances of the latest admission, gives carers expertise. |
| Caregivers possessed comprehensive knowledge of the service users. (U)  Vestphal et al., 2023 | Caregivers experienced and perceived that their comprehensive knowledge of the service user was a potential facilitator in their involvement in care and treatment. However, when the caregiver felt ignored, despite such knowledge, and excluded by HCPs from care and treatment, this could lead to feelings of anger, disempowerment and distrust ... |

**Category: A strong sense of responsibility and persistent caring roles**

(Carers felt a profound and persistent sense of duty in supporting the service users before, during, and after engaging in mental health services.)

| **Findings, citations, and credibility rating** | **Verbatim excerpts of the source reviews** |
| --- | --- |
| Carers mediated between service users and health professionals. (U)  Bartl et al., 2024 | Some carers described mediating between the person they care for and health professionals. |
| Carers felt a strong sense of duty, supporting their relatives before, during, and after hospitalisation. (U)  Bartl et al., 2024 | Carer participants expressed a sense of duty towards their family member which they willingly took on. While some carers initiated or advocated for admission, some also remained vigilant to protect their relative following admission and resumed 24-hour responsibility following discharge. |
| Families felt that they were protecting the family member by admitting them. (E)  Sugiura et al., 2020 | Some families felt that they were protecting the family member by signing and admitting them to the hospital. |
| Caregivers reported a sense of responsibility for the person they cared for. (U)  Vestphal et al., 2023 | This theme represented characteristics of caregivers’ sense of responsibility and burden in relation to the service user. |
| Most caregivers were very persistent in caring for the service user. (U)  Vestphal et al., 2023 | This theme was characterized by most caregivers being very persistent in taking care of the service user, to a point where they lived their lives with an almost constant focus on their ill family member. |
| Caregivers advocated for the protection and rights of the service user as they felt the need. (U)  Vestphal et al., 2023 | .. some caregivers were of the perception that the service user was unfairly treated and/or victimized by the healthcare system or by HCPs, which could create the foundation for, what may be described as caregivers advocating for the protection and rights of the service user. |

**Synthesised finding 5: Complex experiences of engaging with mental health services**

**Category: Dissatisfaction and distrust**

(Regarding engagement with mental health services, carers expressed dissatisfaction with and distrust in the quality of care.)

| **Findings, citations, and credibility rating** | **Verbatim excerpts of the source reviews** |
| --- | --- |
| Carers expressed dissatisfaction with multiple aspects of mental health services. (U)  AbouSeif et al., 2022 | Carers spoke of dissatisfaction relating to delays in admission, unmet service user needs, staff competence, the duration of hospitalisation, safety and the lack of space. ... Carers were also displeased with the heavy focus on medicating the service user, |
| Carers felt let down by heavy-handed detention but valued respectful treatment. (U)  Bartl et al., 2024 | The previous review reported that by the time assessment and detention occurred, many carers already felt let down by services. Carers described detention processes as inappropriate/heavy-handed ... Recent reports are also mixed. Carers appreciated a street triage initiative and service users being treated with respect. Carers also reported a lack of community alternatives to inpatient admission, concern about how involuntary admission was managed. |
| Some carers did not trust the services and treatments. (U)  Bartl et al., 2024 | Carers wanted to know that their relatives are safe and receiving care to help them recover, but some carers did not believe that the hospital met their relative’s needs and did not trust the treatment provided. … |
| Parents reported feeling frustrated for negative attributes of health professionals. (U)  Oketah et al., 2023 | ... a lack of feedback from clinicians, which made them unclear about their child’s prognosis during therapy.  ... Parents felt that doctors, nutritionists, social workers, and other members of the medical community who were unfamiliar with AN negatively impacted the diagnosis and treatment of their child. … Parents reported negative attitudes where they felt like they were being blamed or judged ... |
| Carers expressed deep frustration with current services. (U)  Stuart et al., 2020 | Carers were desperate to get earlier, preventative help rather than having to wait until patients were in crisis and posed a risk of harm to themselves or others. By the time assessment and detention occurred many carers already felt let down by health services. While acknowledging the necessity of detention at that point, they had profound misgivings. |
| Families felt that the services were degrading for the service users. (U)  Sugiura et al., 2020 | Families described the experience of using the mental health system as harmful and degrading for family members |

**Category: Neglect of needs and lack of adequate support**

(Carers frequently reported experiencing neglect and inadequate support from services.)

| **Findings, citations, and credibility rating** | **Verbatim excerpts of the source reviews** |
| --- | --- |
| Carers were seldom acknowledged or supported. (U)  AbouSeif et al., 2022 | Despite the significant emotional toll of the hospitalisation and a heightened need for support, carers report that their needs were seldom acknowledged and strongly perceived inpatient staff as unsupportive. They were not offered support or asked about their own needs by staff. |
| Carers needed proactive support. (U)  Bartl et al., 2024 | For their own health, carers need support to be offered proactively, before, during and after detention, to help them make sense of the illness, deal with stress, and accept their situation. |
| Partners were dismissed and left out by the serivice. (U)  Lever Taylor et al., 2018 | A key theme … related to the exclusion, marginalization, or neglect of women’s partners by the system and by healthcare professionals. …… |
| Cares needed support throughout the process, but the lack of adequate support for carers was prominent. (U)  Stuart et al., 2020 | The lack of adequate support for carers of patients being assessed or detained under mental health legislation was a prominent theme across all papers. … Carers reported that they needed support not only in the period prior to assessment and detention but also during this time and afterwards, including to understand what had happened and their role in it. |
| Families struggled to be recognized by professionals in care. (U)  Sugiura et al., 2020 | Families struggled to be recognized by professionals in care and often felt that they were undervalued. |

**Category: Exclusion from care and decision-making**

(Carers frequently reported being excluded from care and decision-making for their loved ones in mental health services.)

| **Findings, citations, and credibility rating** | **Verbatim excerpts of the source reviews** |
| --- | --- |
| Carers felt excluded from inpatient care. (U)  AbouSeif et al., 2022 | ..., carers felt excluded from inpatient care. Carers reported being informed of decisions regarding their loved one’s care last minute or after the fact, ... Carers felt that their expertise was not drawn upon or included in clinical decision making. |
| Carers attempted to participate in care and decision making, but often failed. (U)  Bartl et al., 2024 | Following involuntary admission carers reported that they tried to maintain dialogue with hospital staff and participate in treatment and discharge decisions but were often not being heard. |
| Many carers reported to be informed about discharge at the last minute. (U)  Bartl et al., 2024 | ... many carers reported that they were informed about discharge at the last minute when neither the service user nor family was ready. |
| Families felt being excluded from provisions of care. (U)  Rodell & Parry, 2024 | Within family narratives, feeling excluded from provisions of care was expressed ... Although the importance of confidentiality was recognised, frustration was expressed nonetheless ... |
| The extent of carers’ involvement in decision-making and the provision of care was a key influence on carers’ experiences under mental health legislation, but their involvement was often limited. (U)  Stuart et al., 2020 | The extent to which carers felt involved in decision-making and the provision of care was a key influence on carers’ experiences of supporting patients detained under mental health legislation. ...Yet, information was often lacking. ... After detention it was a source of frustration to many carers to not be involved in discharge planning ... |
| Caregivers felt being excluded from the service users’ care and treatment. (U)  Vestphal et al., 2023 | Caregivers’ experiences of feeling excluded from the service user’s care and treatment characterized this theme. |

**Category: Unmet need for information and communication**

(Carers frequently reported unmet needs for information and communication.)

| **Findings, citations, and credibility rating** | **Verbatim excerpts of the source reviews** |
| --- | --- |
| Carers wanted to share and receive information, while the absence of information was frequently reported. (U)  Bartl et al., 2024 | To maximise treatment and recovery, carers want to share information and described the importance of receiving information from clinicians about the illness and the service user’s progress. Carers frequently reported the absence of information … |
| Confidentiality prevented carers from receiving information. (U)  Bartl et al., 2024 | Carers stated that confidentiality prevented them from receiving information that would help them take care of their relative. |
| Partners reported an unmet need for information. (U)  Lever Taylor et al., 2018 | A second, related theme centred on a lack of relevant information for partners. Partners said they wanted to help but did not know how and “had no idea how to get help”. |
| Family members were disappointed at the lack of information or guidance. (U)  Reupert et al., 2023 | ... parents, children, and other relatives were all disappointed at the lack of information or guidance about children visiting their parents in the hospital. |

**Category: Limitations of current mental health service models**

(Carers raised concerns about inadequate staff competencies and service models that were inappropriate or culturally insensitive.)

| **Findings, citations, and credibility rating** | **Verbatim excerpts of the source reviews** |
| --- | --- |
| Carers viewed staff in respite services as having insufficient skills. (U)  Almoaber et al., 2024 | … carers viewed staff who worked in respite services as having insufficient skills to care for people with MI. |
| Carers described respite services as inadequately tailored to service users’ needs. (E)  Almoaber et al., 2024 | Carers and patients with MI in the studies under consideration described respite services as providing care for a variety of people simultaneously, ...; ..., the activities offered tended to be unsuitable for all participants … |
| There is a general lack of attention to cultural diversity in current services. (E)  Almoaber et al., 2024 | This review also uncovered a general lack of attention to cultural diversity because of the inappropriate service model for respite care. ... A lack of cultural understanding among mental health workers and regarding Indigenous Australians has also raised a barrier to accessing respite care. |

**Category: Expectations of health professionals and services**

(Carers expected healthcare professionals to be empathetic, respectful, and compassionate.)

| **Findings, citations, and credibility rating** | **Verbatim excerpts of the source reviews** |
| --- | --- |
| Carers wanted staff to be more affectionate and caring to their loved ones. (U)  AbouSeif et al., 2022 | They spoke of wanting staff to be more affectionate and caring to their loved one, to actively listen and talk to them, and to help them with self-care. |
| Carers valued professionals’ empathy, competence, availability, and respectful attitudes. (U)  Oketah et al., 2023 | Availability, competence, and positive attitudes like empathy, sensitivity, respectfulness, friendliness, and trustworthiness were especially highlighted in six studies and were deeply appreciated by parents. |

**Category: Variations in views and experiences**

(It was noticeable that carers expressed ambivalence about their involvement and support needs and reported mixed experiences with various treatment modalities.)

| **Findings, citations, and credibility rating** | **Verbatim excerpts of the source reviews** |
| --- | --- |
| Partners felt ambivalent about involvement, wanting involvement while minimising their own needs. (U)  Lever Taylor et al., 2018 | Although partners reported feeling underinformed and neglected, there was at the same time a somewhat competing theme related to their own ambivalence about being better included and attended to. …, a majority of the studies reviewed (n = 14) also depicted partners themselves as struggling to identify their own needs, minimizing their own desires, prioritizing the woman, or expressing a reluctance to ask for or accept help or involvement. |
| Partners held mixed views on support, with unclear preferences and conflicting feedback. (U)  Lever Taylor et al., 2018 | Mirroring partners’ ambivalence around involvement and support, there was also divergence or discrepancy apparent between and even within studies about what support would be appropriate for partners. |
| Parents reported different experiences with diverse treatment modalities. (U)  Oketah et al., 2023 | Overall, FBT was seen as positive by parents, ... …, MFT was viewed positively by some parents  Other studies highlighted negative aspects of experiences with FBT/MFT, ...  Home treatment … Parents experienced containment, provision of hope, an opportunity to find strength, and a reduction in the burden of care. On the negative side, parents reported fear and anxiety about the future   Inpatient treatment … parents’ experience of inpatient treatment as inappropriate as it was in a general ward. … Feelings of guilt were exacerbated by not being with their child constantly …  Compulsory treatment/control they were conflicted between acknowledging that compulsory treatment was justified and wanting their child to be treated with dignity (their consent), … |

**Synthesised finding 6: Relational strain during the caregiving process**

**Category: Strained relationships between carers and service users**

(Engagement with mental health services frequently led to emotional and relational distance between carers and the individuals they supported.)

| **Findings, citations, and credibility rating** | **Verbatim excerpts of the source reviews** |
| --- | --- |
| Hospitalisation led to a strain on the relationship between carers and service users. (U)  AbouSeif et al., 2022 | Carers reported that with the hospitalisation, a strain was placed on their relationship with the service user. … For some, this was linked to blame, ... Others cited an inability to visit their loved one as often as desired due to practical difficulties, ... |
| Some studies described relationship break-down between carers and service users following involuntary admission. (U)  Bartl et al., 2024 | Carers spoke of trying to make sense of the illness in the context of family relationships. … some studies described communication breaking down between carers and service users following involuntary admission: mistrust and rejection by service users; losing touch; or carers cutting off contact after admission ... |
| Some carers described experiences of power in their role and the power unbalanced their relationship with their family members. (U)  Bartl et al., 2024 | Carers described experiences of power in their role. ... Some carers felt distressed that their powers as a Nearest Relative ... unbalanced their relationship with their relative .... Many carers felt powerless, but some viewed their relative’s admission as forced by them and some used their relative’s economic dependency as leverage to engage in treatment. |
| Specialist services often led to physical and emotional distance between carers and service users. (U)  Rodell & Parry, 2024 | ... escalation of symptoms frequently created a physical and emotional distance as primary care was handed over to specialist services, leaving a sense of loss.... |
| Detention under mental health legislation could lead to the breakdown of relationships between the patients and carers. (U)*   Stuart et al., 2020 | The detention of a patient could lead to the breakdown of the relationship between the patient and carer. |
| Families felt that signing the involuntary admission would ruin their relationship with the servuce users. (U)  Sugiura et al., 2020 | Some families found it hard to sign a form to admit their family member because they felt as if they were putting the person away. They also worried that becoming the applicant could damage their relationship with the service user. |

**Category: Unsatisfactory relationships between carers and health professionals**

(Carers reported unsatisfactory interactions with healthcare professionals.)

| **Findings, citations, and credibility rating** | **Verbatim excerpts of the source reviews** |
| --- | --- |
| Carers felt a tension between staff and themselves, while partnership and cooperation were desired. (U)  AbouSeif et al., 2022 | They described the relationship as marked with tension and reported sensing a divide of “us vs them”, wherein they were viewed as threats, challenging, or nuisances. Nonetheless, carers consistently expressed a desire for partnership with staff, … |
| Carers wanted partnership with health professionals and appreciated kind and respectful professionals, while some reported neglect and unpleasant interactions. (U)  Bartl et al., 2024 | Carers wanted to be able to work in partnership with health professionals and appreciated professionals who were kind and respectful. Others experienced staff as lacking empathy, compassion, or time to talk; and failing to listen or take carers seriously. |
| Many carers described their relationships with healthcare professionals as unsatisfactory. (U)*  Stuart et al., 2020 | Across several papers many... carers described their relationships with healthcare professionals, ... as having been unsatisfactory. |

**Findings Not Included in the Final Synthesis**

| **Findings, citations, and credibility rating** | **Verbatim excerpts of the source reviews** |
| --- | --- |
| Immersing in one’s own business acted as a protective factor for siblings in early adulthood during the prodromal phase. (U)  Dawson et al., 2013 | From the prodromal phase until the time of their sibling’s diagnosis of schizophrenia, most well siblings were embracing their independence and were immersed in other things, such as schooling and work, as the majority were in adolescence and early adulthood. |
| Practical barriers hindered partners from accessing support. (U)  Lever Taylor et al., 2018 | ... practical difficulties could present a barrier to partners being involved or accessing support, particularly where the woman’s care was not local. |
| Parents observed positive changes during recovery. (U)  Oketah et al., 2023 | Six studies reported parents observing an overall positive outlook with recovery, personality change, better engagement with the family, and increased confidence with recovery. |
| Families found family group conferencing (FGC) helpful in promoting understanding and avoiding coercive measures. (U)  Sugiura et al., 2020 | Families found FGC to be helpful in finding mutual understanding with neighbors and avoiding coercive measures including involuntary admission. |
| Some carers felt themselves grow from the caring experience. (NS)  Rodell & Parry, 2024 | ... feeling they had “grown from their experiences” through challenging their own negative pre-conceptions … |
| Family members experienced isolation in their caring roles. (NS)  Rodell & Parry, 2024 | While family members rarely voiced feeling isolated explicitly, isolation ran through their stories. |

**E. Evidence and data underpinning the narrative confidence assessment (Tables E1-E3)**

**Table E1. The diversity of mental health service contexts and geographical settings represented by the included reviews.**

| **References** | **Service and clinical contexts of carers’ service experiences** | **Geographical locations of the primary studies included in the reviews** |
| --- | --- | --- |
| **1. AbouSeif et al., 2022 [15]** | inpatient care/hospitalisation | Canada, Brazil, UK, India, South Korea, Australia |
| **2. Almoaber et al., 2024 [18]** | respite care services | Australia only |
| **3. Bartl et al., 2024 [29]** | involuntary admission and detention | Australia (Brisbane), England, Germany, Norway, Republic of Ireland, South Korea, and the USA (Connecticut) |
| **4. Cairns V.A. et al., 2015 [24]** | psychosis | within both urban and rural communities in the UK, Canada, USA, Hong Kong, Brazil, Australia, and New Zealand |
| **5. Dawson et al., 2013 [19]** | psychosis | USA, UK, Canada, Brazil, Australia, Korea, Germany, Hong Kong, Japan, New Zealand |
| **6. Lever Taylor et al., 2018 [25]** | perinatal mental health care | US, UK, Australia, Canada, Sweden, Japan, Finland |
| **7. Oketah et al., 2023 [28]** | anorexia nervosa | Canada, UK, Australia, China, Ireland, Sweden, France, Norway, US |
| **8. Reupert et al., 2023 [26]** | inpatient care/hospitalisation | There were two papers each from Australia and Norway, and the other four were from Sweden, Iran, the USA, and the UK. |
| **9. Rodell & Parry, 2024 [23]** | psychosis | Australia, Canada, France, Greece, Ivory Coast, Mexico, New Zealand, Norway, Portugal, Saudi Arabia, South Africa, Sweden, UK, USA |
| **10. Stuart et al., 2020 [30]** | involuntary admission and detention | UK, Germany, Greece, Norway, the Republic of Ireland, Canada, the USA, and Australia. |
| **11. Sugiura et al., 2020 [27]** | involuntary admission | UK, Norway, Ireland |
| **12. Vestphal et al., 2023 [31]** | forensic mental health care | Finland, UK, South Africa, Canada, Belgium, Denmark |

**Table E2. Evidence underpinning the narrative confidence assessment of the synthesised findings**

| **Synthesised finding** | **Category** | **Confidence Assessment** | | | | | | | |  |
| --- | --- | --- | --- | --- | --- | --- | --- | --- | --- | --- |
|  |  | **Methodological quality of contributing reviews** | **Quality of support from primary evidence reported in reviews** | **number of extractions contributing to each category** | **number of reviews contributing to each category** | **number of contexts contributing to each category** | **number of extractions contributing to each synthesised finding** | **number of reviews contributing to each synthesised finding (total number of reviews = 12)** | **number of contexts contributing to each synthesised finding (total number of contexts = 7)** |  |
|  |  |  |  |  |  |  |  |  |  |  |
|  |  |  |  |  |  |  |  |  |  |  |
| Emotional complexity in the caregiving journey | High emotional distress during the build-up to help-seeking and accessing services | High | Unequivocal | 8 | 7 | 5 | 24 | 11 | 7 |  |
|  | Mixed and complicated emotional responses to service involvement | High | Unequivocal | 13 | 7 | 5 |  |  |  |  |
|  | Uncertainty and guarded hope about the future | High | Unequivocal | 3 | 2 | 2 |  |  |  |  |
| Recognition and coping with their loved ones’ mental health difficulties | Early recognition, reactions, and management | High | Unequivocal | 7 | 3 | 2 | 10 | 4 | 2 |  |
|  | Boosted help-seeking after reaching “crisis point” | High | Unequivocal | 3 | 3 | 1 |  |  |  |  |
| Multiple barriers for carers in accessing mental health support | Systematic barriers to accessing appropriate mental health support | High | Unequivocal | 6 | 4 | 3 | 25 | 7 | 4 |  |
|  | The uneasy and continuous process of sourcing services | High | Unequivocal | 3 | 3 | 2 |  |  |  |  |
|  | Lack of information and knowledge | High | Unequivocal | 5 | 5 | 3 |  |  |  |  |
|  | Complex personal and relational barriers | High | Unequivocal | 11 | 6 | 4 |  |  |  |  |

*(continued)*

**Table E2** (Continued)

| **Synthesised finding** | **Category** | **Confidence Assessment** | | | | | | | |  |
| --- | --- | --- | --- | --- | --- | --- | --- | --- | --- | --- |
|  |  | **Methodological quality of contributing reviews** | **Quality of support from primary evidence reported in reviews** | **number of extractions contributing to each category** | **number of reviews contributing to each category** | **number of contexts contributing to each category** | **number of extractions contributing to each synthesised finding** | **number of reviews contributing to each synthesised finding (total number of reviews = 12)** | **number of contexts contributing to each synthesised finding (total number of contexts = 7)** |  |
| Persistent responsibilities and multiple roles in supporting service users | Experiential knowledge and potential to help | High | Unequivocal | 2 | 2 | 2 | 8 | 3 | 2 |  |
|  | A strong sense of responsibility and persistent caring roles | High | Unequivocal | 6 | 3 | 2 |  |  |  |  |
| Complex experiences of engaging with mental health services | Dissatisfaction and distrust | High | Unequivocal | 6 | 5 | 3 | 29 | 10 | 7 |  |
|  | Neglect of needs and lack of adequate support | High | Unequivocal | 5 | 5 | 3 |  |  |  |  |
|  | Exclusion from care and decision-making | High | Unequivocal | 6 | 5 | 4 |  |  |  |  |
|  | Unmet need for information and communication | High | Unequivocal | 4 | 3 | 3 |  |  |  |  |
|  | Limitations of current mental health service models | Moderate | Equivocal | 3 | 1 | 1 |  |  |  |  |
|  | Expectations of health professionals and services | High | Unequivocal | 2 | 2 | 2 |  |  |  |  |
|  | Variations in views and experiences | High | Unequivocal | 3 | 2 | 2 |  |  |  |  |
| Relational strain during the caregiving process | Strained relationships between carers and service users | High | Unequivocal | 6 | 5 | 3 | 9 | 5 | 3 |  |
|  | Unsatisfactory relationships between carers and health professionals | High | Unequivocal | 3 | 3 | 2 |  |  |  |  |

**Table E3. Evidence underpinning the narrative confidence assessment of the synthesised findings**

| A | B | C | D | E | F | G | H | I | J | K | L | M | N | O | P | Q | R | S | T |
| --- | --- | --- | --- | --- | --- | --- | --- | --- | --- | --- | --- | --- | --- | --- | --- | --- | --- | --- | --- |
| **Synthesised findings** | **References   Categories** | **1. AbouSeif et al., 2022 [15]** | **2. Almoaber et al., 2024 [18]** | **3. Bartl et al., 2024 [29]** | **4. Cairns V.A. et al., 2015 [24]** | **5. Dawson et al., 2013 [19]** | **6. Lever Taylor et al., 2018 [25]** | **7. Oketah et al., 2023 [28]** | **8. Reupert et al., 2023 [26]** | **9. Rodell & Parry, 2024 [23]** | **10. Stuart et al., 2020 [30]** | **11. Sugiura et al., 2020 [27]** | **12. Vestphal et al., 2023 [31]** | **number of extractions contributing to each category** | **number of reviews contributing to each category** | **number of contexts contributing to each category** | **number of extractions contributing to each synthesised finding** | **number of reviews contributing to each synthesised finding (total number of reviews = 12)** | **number of contexts contributing to each synthesised finding (total number of contexts = 7)** |
| Emotional complexity in the caregiving journey | High emotional distress during the build-up to help-seeking and accessing services | 1 | 1 | 1 | 2 | 1 |  |  |  | 1 |  |  | 1 | 8 | 7 | 5 | 24 | 11 | 7 |
|  | Mixed and complicated emotional responses to service involvement | 1 | 1 | 3 |  |  | 1 | 4 | 2 |  | 1 |  |  | 13 | 7 | 5 |  |  |  |
|  | Uncertainty and guarded hope about the future |  |  |  |  |  |  | 2 |  | 1 |  |  |  | 3 | 2 | 2 |  |  |  |
| Recognition and coping with their loved ones’ mental health difficulties | Early recognition, reactions, and management |  |  |  | 1 | 5 |  | 1 |  |  |  |  |  | 7 | 3 | 2 | 10 | 4 | 2 |
|  | Boosted help-seeking after reaching “crisis point” |  |  |  | 1 | 1 |  |  |  | 1 |  |  |  | 3 | 3 | 1 |  |  |  |
| Multiple barriers for carers in accessing mental health support | Systematic barriers to accessing appropriate mental health support |  | 2 | 1 |  | 1 |  |  |  | 2 |  |  |  | 6 | 4 | 3 | 25 | 7 | 4 |
|  | The uneasy and continuous process of sourcing services |  |  |  |  | 1 |  |  |  | 1 |  | 1 |  | 3 | 3 | 2 |  |  |  |
|  | Lack of information and knowledge |  | 1 | 1 |  | 1 |  |  |  | 1 |  | 1 |  | 5 | 5 | 3 |  |  |  |
|  | Complex personal and relational barriers |  | 1 | 1 | 1 | 5 |  | 1 |  | 2 |  |  |  | 11 | 6 | 4 |  |  |  |
| Persistent responsibilities and multiple roles in supporting service users | Experiential knowledge and potential to help |  |  | 1 |  |  |  |  |  |  |  |  | 1 | 2 | 2 | 2 | 8 | 3 | 2 |
|  | A strong sense of responsibility and persistent caring roles |  |  | 2 |  |  |  |  |  |  |  | 1 | 3 | 6 | 3 | 2 |  |  |  |
| Complex experiences of engaging with mental health services | Dissatisfaction and distrust | 1 |  | 2 |  |  |  | 1 |  |  | 1 | 1 |  | 6 | 5 | 3 | 29 | 10 | 7 |
|  | Neglect of needs and lack of adequate support | 1 |  | 1 |  |  | 1 |  |  |  | 1 | 1 |  | 5 | 5 | 3 |  |  |  |
|  | Exclusion from care and decision-making | 1 |  | 2 |  |  |  |  |  | 1 | 1 |  | 1 | 6 | 5 | 4 |  |  |  |
|  | Unmet need for information and communication |  |  | 2 |  |  | 1 |  | 1 |  |  |  |  | 4 | 3 | 3 |  |  |  |
|  | Limitations of current mental health service models |  | 3 |  |  |  |  |  |  |  |  |  |  | 3 | 1 | 1 |  |  |  |
|  | Expectations of health professionals and services | 1 |  |  |  |  |  | 1 |  |  |  |  |  | 2 | 2 | 2 |  |  |  |
|  | Variations in views and experiences |  |  |  |  |  | 2 | 1 |  |  |  |  |  | 3 | 2 | 2 |  |  |  |
| Relational strain during the caregiving process | Strained relationships between carers and service users | 1 |  | 2 |  |  |  |  |  | 1 | 1 | 1 |  | 6 | 5 | 3 | 9 | 5 | 3 |
|  | Unsatisfactory relationships between carers and health professionals | 1 |  | 1 |  |  |  |  |  |  | 1 |  |  | 3 | 3 | 2 |  |  |  |

*Note.* The number in column C – column N are numbers of extracted findings contributed by each review to each category. Column O, P, R, S are sum of data based on the data in column C – column N. Data in column Q and column T was derived by combining the review-level information in Table E1 with the extraction-level contribution data in Table E3 column C – column N.
