# Supplementary material for: Informal carers’ experiences of mental health services regarding supporting those they care for and getting support for their own wellbeing: A systematic umbrella review of qualitative evidence
Source: PLOS Ment Health. 2026 Oct 15;3(10):e0000740. doi: 10.1371/journal.pmen.0000740 (PMC13671576; doi:10.1371/journal.pmen.0000740)
Supplement: Supplementary file 2 [file pmen.0000740.s002.docx]

Dear PLOS Mental Health Editorial Team,

Re Editors’ query:

*Thank you for mentioning your previous submission in you cover letter. However may I request for you to please include a rebuttal letter with a response to the decision and reviewers’ comments (if applicable), detailing any revisions made to the original submission. Please upload this as a 'Response to Reviewers' file type.*

As we explained in our covering letter: we submitted a previous version of this paper to PLOS MH on 1^st^ March. It was sent back to us because the journal was unable to find a handling editor. However, we have since been invited to resubmit by the Executive Editor, Dr Karli Montague-Cardoso, who has kindly offered to oversee the consideration of our paper this time. There is no response to reviewers we can make, because you never got our paper out to peer review.

Thank you very much for considering our paper now. We appreciate your time and look forward to your response.

Yours sincerely,

Brynmor Lloyd-Evans

-----------------------------------------------

Brynmor Lloyd-Evans

Professor of Mental Health and Social Inclusion

Division of Psychiatry

University College London

Email: [b.lloyd-evans@ucl.ac.uk](mailto:b.lloyd-evans@ucl.ac.uk)
