# Supplementary material for: Informal carers’ experiences of mental health services regarding supporting those they care for and getting support for their own wellbeing: A systematic umbrella review of qualitative evidence
Source: PLOS Ment Health. 2026 Oct 15;3(10):e0000740. doi: 10.1371/journal.pmen.0000740 (PMC13671576; doi:10.1371/journal.pmen.0000740)
Supplement: Supplementary file 3 [file pmen.0000740.s003.docx]

September 2, 2026

Dear Editor,

“Informal carers’ experiences of mental health services regarding supporting those they care for and getting support for their own wellbeing: a systematic umbrella review of qualitative evidence” (Manuscript ID: PMEN-D-26-00246)

Xiaoye Liu, Phoebe Barnett, Patrick Nyikavaranda, Laura Burley, and Brynmor Lloyd-Evans.

We are very grateful for the reviewers’ constructive comments to improve the manuscript. We have carefully considered all the comments and addressed them as fully as possible. In the following part of this letter, we respond to each point raised by the reviewers and describe how it was addressed in the revision. For ease of reference, changes in the revised manuscript are highlighted with the Word track changes function. In this response, line numbers for changes refer to the track-changed document. In this response letter, our responses to the reviewers’ comments are presented in blue, while *the corresponding revised text in the manuscript is presented in italics*.

**Reviewer #1:** This manuscript presents a systematic umbrella review of qualitative review evidence on informal carers’ experiences of mental health services, both in supporting the individuals they care for and in seeking support for their own wellbeing. The topic is important and clearly relevant to PLOS Mental Health. The manuscript is generally well organised, follows a recognised umbrella review approach, and attempts to use JBI-informed methods, quality appraisal and ConQual confidence assessment. However, I think the manuscript requires substantial revision before it can be considered for publication. My main concerns relate to methodological transparency, consistency in reporting, the handling of heterogeneous review types, the assessment of confidence, and the need for a more nuanced discussion of implications across different mental health service contexts.

Thank you for taking the time to appraise our review carefully, and for the positive comments. We hope we have addressed the overall reservations in our point-by-point response below.

Major comments
1. There is an inconsistency regarding language restrictions.
In the eligibility criteria, the authors state that “There were no restrictions on language, geographic location, or date of the reviews.” However, the results later state that all included reviews were published in English. This needs clarification. If no language restriction was applied, the authors should explain whether non-English reviews were identified and excluded for other reasons, or whether none were retrieved. If the search strategy or databases effectively limited retrieval to English-language publications, this should be acknowledged as a limitation. At present, the wording gives the impression of a broader search than may actually have been achieved.

Thank you for this helpful observation. As stated in our review, no language or geographic restriction was applied during the search. Some non-English reviews were identified and screened but were excluded because they did not meet the eligibility criteria, rather than because of their language. We have revised the Results section to make this explicit and avoid any implication that the search was limited to English-language publications (pp.10-11, lines 242-244).

For instance, below is a Japanese review excluded at the full-text screening stage because it examined the experiences of informal carers of individuals with mental health difficulties in general rather than focusing on experiences of accessing or engaging with mental health services, while more non-English reviews were excluded at the title/abstract screening stage:

Kageyama M. The process of family experiences in caring for persons with serious mental illnesses: common stages identified through a literature review. *Journal of Japan Academy of Nursing Science*. 2012;32(4):63-70. doi:10.5630/jans.32.4_63.

***Corresponding revision:***

*(pp. 10-11, lines 242-244)* *Although the search imposed no language restrictions, all included reviews were published in English. Non-English records were identified but excluded because they did not meet the eligibility criteria.*

2. The title/abstract screening procedure needs stronger justification.
The manuscript reports that title and abstract screening was undertaken by one reviewer, with a second reviewer double-screening only 10% of records, although agreement was high. For a systematic umbrella review, this may raise concerns about missed eligible reviews. The authors should justify this approach more clearly and consider providing additional reassurance, for example by reporting the number of records checked by the second reviewer, disagreements identified, and whether any calibration was conducted before screening. If feasible, a larger proportion of records could be double-screened.

Thank you for the comment. We acknowledge that independent screening of all studies by two researchers is ideal at the title and abstract stage. However, we note that the Cochrane Handbook – accepted best practice guidance for systematic review methods – says this is only essential at the full-text screening stage. To address the reviewers’ concerns:

1) We have revised the Methods to provide a clearer description of the title and abstract screening process and to offer additional reassurance regarding its reliability (p. 6, lines 129–137).

2) We have acknowledged and discussed that we did not double-screen all records at title and abstract stage in the Limitations section of the discussion (p.52, lines 1033-1040).

3) To assure ourselves and double-check the adequacy of our title and abstract screening, we have now double-screened a further 10% of records, i.e. 20% (557 records) in total. This resulted in a high level of agreement (*κ = 0.94*), with 35 discrepancies identified in total. We realize this could overestimate agreement, as the second reviewer had participated in later stages of the review process and, for this additional screening, was aware of which studies were ultimately included. Therefore, this additional post-hoc screening was not reported in the manuscript as formal evidence, but we provide it here as supplementary information.

***Corresponding revision:***

*(p. 6, lines 129–137)* *Two independent reviewers (XYL and LB) discussed the eligibility criteria and conducted a pilot screening exercise to ensure a consistent understanding and application of the eligibility criteria. Screening of titles and abstracts was undertaken by one independent reviewer (XYL), consistent with Cochrane guidance that initial title and abstract screening may be conducted by a single reviewer [22]. A second reviewer (LB) double-screened 285 results, approximately 10% of the results screened at the title and abstract level, to ensure consistency. 35 discrepancies were identified and resolved through discussion, achieving strong agreement (κ = 0.88).*

*(p.52, lines 1033-1040)* *Third,* *during the study selection process, only 10% of the retrieved records were independently double-screened at the title and abstract stage due to resource constraints. This approach is consistent with* *Cochrane guidance, which states that while duplicate screening at the title and abstract stage is desirable, it is not essential [22]. The high level of agreement achieved during the 10% double-screening suggests a slim likelihood of missing relevant records. Nevertheless, it is acknowledged that duplicate screening of all records would have been desirable. Future reviews should consider implementing duplicate screening throughout the entire study selection process where feasible to further minimise the risk of selection bias.*

3. The inclusion of different types of reviews requires further explanation.
The eligibility criteria refer to published systematic reviews or qualitative meta-syntheses, but the included studies also contain one integrative literature review and one narrative review. The authors should clarify why these review types were eligible and how they meet the threshold for inclusion in an umbrella review. This is particularly important because the methodological standards and reporting quality of narrative/integrative reviews may differ from those of systematic qualitative syntheses. A sensitivity analysis or a clearer justification of their influence on the synthesis would strengthen the manuscript.

Thank you for the comment. In order not to exclude potentially relevant evidence, we included any form of literature review which involved searching at least two bibliographic databases with transparent search strategies, in line with our pre-registered protocol. The eligibility criteria in the Methods section were revised accordingly for clarification (p.5, lines 107-108).

However, we agree with the reviewer that there is a risk that findings from less methodologically robust types of literature reviews could potentially undermine the validity of our findings. To address the reviewer’s comment, we have added a brief discussion in the Limitations section, explaining the rationale for our inclusion of a range of types of review and the steps we have now taken to check that findings from non-systematic reviews do not have undue weight in our findings (pp. 51-52, lines 1013-1032). We have gone back and checked our categories and synthesised findings. No synthesised findings rely solely on findings from the narrative or integrative reviews. Only one category was generated solely from the integrative review (Almoaber et al.): “Limitations of current mental health service models” within the fifth synthesised finding “Complex experiences of engaging with mental health services”. No categories were generated solely from the narrative review.

***Corresponding revision:***

*(pp. 51-52, lines1013-1032)* *Second, to avoid missing potentially relevant evidence, any form of literature review which involved searching at least two bibliographic databases with transparent search strategies was included in this umbrella review. It is acknowledged that including reviews with different methodological approaches may introduce variability in methodological rigour and reporting quality. Thus, to examine the potential influence of reviews with different methodological approaches on the robustness of our synthesised findings, an additional assessment of the contribution of findings from the narrative review by Rodell and Parry [26] and the integrative review by Almoaber et al. [18] was done. All findings informed by Rodell and Parry’s narrative review [26] were also supported by one or more systematic reviews. Therefore, excluding this review would not have materially altered the synthesised findings or the overall conclusions. For Almoaber et al. [18], most contributions were similarly supported by systematic reviews. The only exception was the category “Limitations of current mental health service models” within the fifth synthesised finding “Complex experiences of engaging with mental health services”, which was derived solely from this integrative review. Excluding this review would have reduced the comprehensiveness of the synthesis by removing insights into carers’ experiences of structural limitations in mental health service provision, including concerns regarding staff expertise, the suitability of respite services, and the cultural responsiveness of care. Moreover, the broader synthesised finding remained supported by multiple systematic reviews, suggesting that the inclusion of this review did not materially affect the overall conclusions of the umbrella review.*

4. Overlap between primary studies across included reviews should be assessed more explicitly. The authors acknowledge in the limitations that overlapping primary studies across included reviews may overrepresent certain findings. However, this issue is only briefly mentioned and does not appear to be systematically assessed. In an umbrella review, overlap can materially affect the interpretation of synthesised findings. The authors should consider mapping primary studies across the included reviews, reporting the degree of overlap, and discussing whether some findings are disproportionately driven by the same primary studies appearing in multiple reviews.

Thank you for this helpful comment. We agree that overlap between primary studies is an important methodological consideration in umbrella reviews, as repeated inclusion of the same primary studies may disproportionately influence the synthesis. To address this, we constructed a citation matrix and calculated the Corrected Covered Area (CCA) to quantify the degree of overlap between primary studies across the included reviews. The calculated CCA was 1.6%, indicating only slight overlap, suggesting that repeated inclusion of the same primary studies was unlikely to have materially and disproportionately influenced the synthesised findings. We have reported the methods used to assess overlap in the Methods section (p. 8, lines 172-182) and the results of the overlap assessment in the Results section (pp. 17-18, lines 296-304).

***Corresponding revision:***

*(p. 8, lines 172-182)* ***Assessment of overlap between primary studies***

*To assess the extent of overlap between primary studies included across the reviews, a citation matrix was constructed, and the Corrected Covered Area (CCA) was calculated [24]. Overlap among primary studies across reviews may result in the over-representation of certain evidence and disproportionately influence the synthesis. The CCA provides an estimate of the degree of overlap by calculating the proportion of repeated primary study occurrences relative to the total number of study occurrences across included reviews [24]. Higher CCA values indicate greater overlap and a greater potential for duplicated evidence to influence the interpretation of findings. CCA values below 5% are generally considered to indicate slight overlap, 5–10% moderate overlap, and above 10% high overlap [25].*

*(pp. 17-18, lines 296-304)* *The lists of primary studies across the included reviews were cross-checked to assess the potential overlap. Across the 12 included reviews, there were 168 total occurrences of primary studies, representing 143 unique primary studies. Among these, 14 primary studies were included in two reviews, 4 were included in three reviews, and 1 was included in four reviews. The degree of overlap was quantified using the* *Corrected Covered Area (CCA) [24]. It was 1.6%, indicating slight overlap.* *This suggests that the included reviews were based largely on independent bodies of primary qualitative evidence and that repeated inclusion of the same primary studies in multiple reviews was unlikely to have materially influenced the synthesis by disproportionately representing particular findings.*

5. The confidence assessment appears too strong and needs more cautious interpretation.
The abstract states that the findings were rated as high confidence. However, the manuscript also notes important limitations, including inconsistent reporting of primary study context, limited demographic information, predominance of high-income country evidence, and disproportionate focus on acute or hospital-based contexts. The authors should explain more clearly how these limitations were incorporated into the ConQual assessment. The current “high confidence” framing may overstate the certainty and transferability of the findings.

Thank you for the comment. There is currently no established certainty assessment framework specifically designed for qualitative umbrella reviews. The ConQual approach is developed for qualitative systematic reviews, assessing confidence in synthesised findings based on the dependability and credibility of contributing primary evidence. However, it does not explicitly account for the breadth of evidence across multiple reviews, variation in study contexts, and the transferability of findings across settings, which are particularly relevant to umbrella reviews.

Thus, we acknowledge the inapplicability of the ConQual approach in the current review in the Limitation section (pp. 53-54, lines 1073–1085).

We have reconsidered our approach following the reviewer’s valid comments. A qualitative narrative assessment of the robustness of the synthesised findings was conducted and presented this instead of applying the original ConQual approach for assessing confidence in our current findings. We have described the methods applied in the Methods section (p. 10, lines 211-226) and the results presented in the Results section (pp. 33-37, lines 628-712) and supplementary material (Supplementary Material, S5 Appendix). The abstract has also been revised correspondingly (p. 2, lines 44-45).

***Corresponding revision:***

*(p. 10, lines 211-226)* ***Assessment of the confidence of synthesised findings***

*To facilitate interpretation of the synthesised findings, an assessment of the breadth and robustness of the supporting evidence was undertaken. As no established framework exists for assessing confidence in synthesised findings in qualitative umbrella reviews,* *a narrative assessment was conducted using review-level information, considering the* *methodological quality of the included reviews,* *quality and adequacy of support, and breadth and diversity of the supporting evidence. Specifically, for each synthesised finding, we examined:* *1) The methodological quality of the contributing reviews;* *2) the extent to which the individual, extracted findings were well supported by primary evidence reported within the included reviews (e.g., links to primary qualitative findings or participant quotations), rated as* *unequivocal (i.e. clearly supported by direct and unambiguous evidence),* *equivocal (**i.e. supported but open to some interpretation), or not supported; 3) the degree of primary study overlap across contributing reviews; 4) the number of contributing extracted findings and reviews to each category and synthesised finding; and 5)* *the diversity of mental health service contexts and geographical settings represented by the contributing evidence.*

*(pp. 33-37, lines 628-712)* ***The confidence of synthesised findings***

*A narrative assessment indicated that confidence varied across the synthesised findings according to the methodological quality, quality and adequacy, breadth, and contextual diversity of the supporting evidence. Overall, confidence was greatest for findings supported by multiple high-quality reviews, numerous extracted findings, evidence from diverse mental health service settings, and primary studies conducted across a broad range of countries.*

*The methodological quality of included reviews was presented in* ***Table 1****, while the* *quality ratings of support from primary data of individual extracted findings were shown in* ***S3 and S4 Appendices****. Drawing on these assessments, the methodological quality of the contributing reviews and the extent to which the contributing extracted findings were supported by primary evidence were summarised for each category and presented in* ***S5 Appendix Table5.2****. At the category level, almost all categories demonstrated high methodological quality of contributing reviews and unequivocal support from primary qualitative evidence.* *One category, “Limitations of current mental health service models”,* *was assessed as having moderate methodological quality and equivocal support, reflecting a comparatively weaker evidence base and warranting cautious interpretation. Across all synthesised findings, confidence was strengthened by the generally high methodological quality of the contributing reviews and by the fact that most contributing reviews explicitly supported their interpretations using primary qualitative data. Together, these characteristics suggest that the umbrella review findings are well grounded in the available qualitative evidence.*

*The overlap assessment indicated slight overlap among primary studies across contributing reviews (CCA = 1.6%), suggesting that the synthesised findings were largely informed by independent bodies of primary qualitative evidence [25]. Therefore, the potential for repeated inclusion of the same primary studies to disproportionately influence the synthesis was considered limited.*

*The service-context and geographical diversity of the primary studies, the number of contributing reviews and extracted findings to categories and synthesised findings, and the level of diversity supporting each synthesised finding were summarised and presented in* ***S5 Appendix****.* *Specifically, service-context and geographical diversity of the primary studies are presented in* ***S5 Appendix Table 5.1****, while the number of contributing extracted findings, reviews, and contexts for each category and synthesised finding are presented in* ***S5 Appendix Table 5.2****.*

*The included reviews synthesised primary qualitative studies from geographically diverse regions* *across Asia, Europe, North America, South America, Africa and Oceania. Only one Australia-focused review applied geographical restriction in primary study inclusion [18]. Despite breadth of included geographical settings in the primary reviews’ inclusion criteria, studies from high-income countries, particularly the United Kingdom, Australia, Canada, and the United States, were disproportionately numerous, with relatively limited representation from low- and middle-income countries. Consequently, the transferability of the findings to resource-constrained health systems should be interpreted with caution.* *Meanwhile, across the included reviews, carers’ experiences were examined in seven service contexts: inpatient care overall, compulsory admission specifically, and five specific clinical or service contexts — psychosis services, perinatal mental health services, eating disorder services, forensic mental health services, and respite care. The evidence was disproportionately derived from acute, inpatient, and high-intensity mental health services. Community-based, lower-intensity services were comparatively underrepresented. As a result, the synthesis primarily reflects carers’ experiences within specialist or crisis-oriented services, which may limit the applicability of some findings to low-intensity mental health settings.*

*Among the six synthesised findings, “Complex experiences of engaging with mental health services” received the strongest support, drawing on 29 extracted findings from 10 reviews representing all seven mental health service contexts. Within this synthesised finding, Dissatisfaction and distrust, Neglect of needs and lack of adequate support, and Exclusion from care and decision-making were each supported by five or more reviews and evidence from multiple service contexts, with contributing reviews generally providing clear links to primary qualitative findings. Similarly, “Emotional complexity in the caregiving journey” was supported by 24 extracted findings from 11 reviews across all seven service contexts, indicating substantial consistency in carers’ emotional experiences despite variation in clinical settings. “Multiple barriers for carers in accessing mental health support” was also supported by a broad evidence base, comprising 25 extracted findings from seven reviews across four mental health service contexts. The convergence of evidence across multiple reviews suggests that barriers to accessing support are common across different mental health services, although the predominance of evidence from high-income countries should be considered when interpreting the transferability of these findings. “Recognition and coping with their loved ones’ mental health difficulties”, “Persistent responsibilities and multiple roles in supporting service users”, and “Relational strain during the caregiving process” were supported by fewer reviews, extracted findings, and service contexts than the strongest themes, indicating somewhat lower confidence, with a narrower but internally consistent evidence base.*

*Confidence was comparatively lower for several categories supported by a more restricted evidence base.* *In particular, “Limitations of current mental health service models” was supported by only one integrative review on respite care services that included only Australian primary studies, with moderate methodological quality and equivocal support. Despite comprising three extracted findings, this category should be interpreted cautiously due to the limited breadth and strength of the supporting evidence. In addition, findings such as “Expectations of health professionals and services”, “Uncertainty and guarded hope about the future”, “Boosted help-seeking after reaching crisis point”, and “Variations in views and experiences” were each informed by only two or three reviews, reducing confidence in their transferability across different settings.*

*Taken together, the synthesised findings are well grounded in the available qualitative evidence, while recognising that confidence is lower for themes supported by fewer reviews or more limited contextual diversity. At the same time, the transferability of the findings to resource-constrained health systems and lower-intensity community services may be constrained by the predominance of high-income countries and acute mental health service settings.*

*(**pp. 53-54, lines 1073–1085)* *Finally, there is currently no established framework for assessing the certainty or confidence of findings in qualitative umbrella reviews. In accordance with the pre-registered protocol, the initial plan was to adapt the ConQual approach to assess confidence in the synthesised findings. ConQual was developed for qualitative systematic reviews synthesising primary qualitative studies and assesses confidence based on the dependability and credibility of the contributing primary evidence [20]. However, it does not explicitly account for methodological considerations that are particularly relevant to umbrella reviews, such as the breadth of evidence across multiple reviews, variation in study contexts, and the transferability of findings across settings. Consequently, a narrative assessment of the robustness of the synthesised findings was undertaken to facilitate interpretation. Nevertheless, this approach has not been formally validated for qualitative umbrella reviews. Further methodological research is needed to develop and evaluate certainty assessment frameworks that are specifically designed for qualitative umbrella reviews.*

6. The contribution beyond existing reviews should be made clearer. The manuscript argues that no comprehensive synthesis has integrated the existing qualitative review literature into a single high-level evidence base. This is a reasonable rationale. However, many of the findings, such as carers’ exclusion from decision-making, lack of information, emotional distress, and difficulty navigating services, are already well established. The authors should more explicitly articulate what new conceptual, methodological, or service-level insight is generated by this umbrella review. For example, the manuscript could better explain whether the review identifies a cross-cutting pathway of carers’ experiences across different service stages, or whether it reveals gaps in existing service models.

Thank you for the comment. We acknowledge that, inevitably, an umbrella review will reflect the findings from the primary systematic reviews it includes. We nevertheless think it adds value by clarifying the extent of consistent findings across a range of service settings, and in which service contexts we can be confident that findings apply. We think our umbrella review does a better job of this with the revised confidence assessment, and thank the reviewer for that helpful prompt. We have revised the Discussion to clarify the contribution of this umbrella review, which integrated existing qualitative review evidence to provide a higher-level conceptual understanding of informal carers’ experiences across the mental health care trajectory. We have also highlighted two findings from our paper which we believe particularly help to increase understanding of carers’ experiences and needs from services. Specifically, we have highlighted how carers’ experiences can be understood as a dynamic caregiving journey across different stages of care (p. 39, lines 751-760), and how the synthesis identifies recurring structural limitations within current service models that require system-level approaches rather than isolated interventions (pp. 45-47, lines 893-924).

***Corresponding revision:***

*(p. 39, lines 751-760)* *Although many of the experiences identified in this review have been reported previously, they have largely been presented within specific mental health conditions, service settings, or stages of service engagement. Rather than representing isolated encounters with services, this umbrella review provides a higher-level conceptual understanding of informal carers’ experiences throughout the mental health care trajectory. The synthesis demonstrates that carers’ experiences can be organised and understood around a dynamic caregiving journey, extending from recognising emerging mental health difficulties, through help-seeking, accessing and engaging with services, to long-term caregiving and ongoing uncertainty about the future.* *Across this journey, carers’ emotional experiences, caring responsibilities, and relationships with both service users and health professionals evolve continuously.*

*(pp. 45-47, lines 893-924)* ***Strengthening system-level approaches to carer support***

*Insufficient information and guidance, fragmented communication, limited involvement in decision-making, inadequate inclusion and respect, and insufficient support directed towards carers themselves were described relatively consistently by carers.* *The recurring nature of these challenges suggests that many of the challenges experienced by carers are structural rather than isolated problems arising within individual services or geographical settings. Also, support for carers should not be conceptualised as a single intervention delivered at one point in the care pathway. Instead, carers require ongoing support that adapts to their changing informational, emotional, and practical needs as their interactions with mental health services evolve.*

*Recent international and national initiatives increasingly emphasise recognising, involving, and supporting informal carers of people with mental health difficulties. For instance, the World Health Organisation Comprehensive Mental Health Action Plan advocates involving families and carers in treatment and providing information, care and support for them [56]. Comparable commitments have been reflected in national mental health policies, such as the UK Government’s Unpaid Carers Action Plan: Recognise, Refer, Reach [57] and New Zealand’s Mental Health and Addiction Service Development Plan [58], which have increasingly recognised informal carers as essential partners in care whose contributions and knowledge should be actively valued and integrated, while also acknowledging their own support needs. However, current findings suggest that the translation of these principles into routine practice remains inconsistent and challenging. Structural limitations identified in the current synthesis are unlikely to be fully addressed by fragmented, service-level improvements. Instead, improvements may require coordinated organisational and system-level approaches that ensure carer-inclusive practice is implemented consistently across different services and stages of the mental health care pathway. Future implementation efforts may benefit from embedding carer-inclusive practice within routine organisational processes and care pathways. This may include establishing consistent standards for information sharing and carer involvement, providing workforce training on collaborative working with carers, and ensuring continuity of support across transitions between services. Such system-level approaches may better address the recurring barriers identified throughout the caregiving journey than isolated interventions delivered within individual services.*

7. The discussion of service implications is currently rather general.
The conclusion recommends improving service accessibility, raising public awareness, providing guidance, facilitating carer involvement, and enhancing professional competence. These are important but broad recommendations. The authors should develop more specific implications for different service contexts, for example inpatient care, compulsory admission, community mental health services, crisis care, discharge planning, and services designed primarily for carers.

Thank you for the comment. We agree that the implications presented in the original manuscript were rather general. The current review applied the meta-aggregation approach, which aims to identify and synthesise common experiences of informal carers across diverse mental health service contexts rather than generate context-specific recommendations. Consequently, the methodology naturally limits our ability to draw implications for individual service settings, which has been acknowledged in the Limitations section of the original manuscript (p. 52, lines 1047–1052). In the revised manuscript, we have made this limitation more explicit (p. 53, lines 1053–1055).

However, in response to this comment, we conducted an additional examination of the contribution of synthesised findings across different service contexts. Where multiple reviews focused on the same service context, we explored whether particular synthesised findings were consistently identified across all reviews within that context and incorporated more context-specific implications into the revised Discussion section (pp. 47-49, lines 925-969). We hope this goes some way to addressing the reviewer’s concern.

***Corresponding revision:***

*(pp. 47-49, lines 925-969)* ***Context-specific service implications***

*Although the findings of this umbrella review primarily represent overarching experiences shared across different mental health service contexts, additional consideration of context-specific patterns identified several areas where tailored service responses may be particularly relevant. To explore this, where multiple reviews focused on the same service context, whether particular synthesised findings were consistently identified across reviews within that context was examined.*

*In three reviews regarding compulsory admission contexts, carers consistently reported dissatisfaction and distrust towards services, unmet needs and insufficient support, and strained relationships with service users. Services should prioritise approaches that promote trust, collaboration, and shared understanding. This may include providing clear and timely psychoeducation and explanations about mental health difficulties and compulsory interventions, and maintaining transparent communication. Also, carers may require specific emotional and practical support to cope with the distress associated with compulsory admission and relationship tension with the person they support. While balancing service-user autonomy and confidentiality with carers’ need for support and information remains essential, flexible approaches to information sharing, such as distinguishing between general information that can be provided without consent and confidential personal information as mentioned earlier, may help [55].*

*In two reviews about inpatient care, carers consistently described mixed emotional responses following service users’ admission, with relief that their loved ones were receiving care often coexisting with uncertainty, guilt, loneliness, and ongoing distress. These complicated and nuanced feelings highlight carers as individuals with their own support needs. Alongside involving carers in treatment where appropriate, services could provide clear explanations of mental health conditions and treatment processes, combined with empathetic acknowledgement and support for carers’ emotional experiences, including guilt, uncertainty, and feelings of isolation. Offering emotional support and signposting carers to peer support or other relevant services during admission may help carers adapt to changing roles, improve competence and confidence in daily care-giving, and better prepare for the transition to community care following discharge.*

*In three reviews of psychosis-related services, the consistent identification of high distress during the help-seeking process, crisis-driven service engagement, and personal and relational barriers to accessing support highlights the importance of proactive carer-facing support before psychosis situations escalate. Although optimal approaches are not yet clearly established, proactive early detection strategies and targeted health education from early psychosis services are warranted to improve service users’ care pathways and outcomes, and make it easier for carers to recognise psychosis and seek help[59]. Once people with psychosis have reached services, community mental health and early intervention services could consider early, proactive engagement with carers, including providing accessible psychoeducation about emerging symptoms, available pathways to support, and strategies for managing relational difficulties. Such approaches are consistent with family intervention models in psychosis, which aim to enhance carers’ understanding, coping capacity, and involvement in recovery-oriented care [60]. Earlier support may help reduce reliance on crisis points as the primary trigger for accessing mental health services and lessen the influence of personal and relational barriers on timely help-seeking.*

(p. 53, lines 1053-1055) *The method has limited ability to inform context-specific recommendations. Although contextual patterns were explored where evidence was available, findings regarding specific service settings should be interpreted cautiously. At the same time, …*

Minor comments
1. There are some typographical and formatting issues that should be corrected, for example “expriences” in Table 1 and occasional spacing issues such as “experiences.A summary table”.

Thank you for pointing this out. We have carefully checked the manuscript for typographical and formatting errors. The typo “expriences” in Table 1 (p.13) and the identified spacing issues, such as “experiences. A summary table” (p.9, line 196), have been corrected. We have also conducted a further check throughout the manuscript to ensure consistency in formatting and spacing.

2. The phrase “the rest reviews” should be revised to “the remaining reviews” or “the rest of the reviews.”

Thank you for the comment. The revision has been done accordingly (p.7, line 147).

3. The authors should ensure consistent use of “synthesised” and “synthesized” throughout the manuscript.

Thank you for the comment. The revision has been done accordingly (p.9, lines 196- 197).

4. It would be helpful to include more detail on the excluded full-text reviews and reasons for exclusion, either in the main manuscript or supplementary materials.

Thank you for the comment. Revisions have been made to the main manuscript (p. 11, lines 230-237). At the same time, a new appendix on records excluded at the full-text stage and the reasons for exclusion has been added to improve transparency (Supplementary Material pp. 7-14, S2 Appendix).

***Corresponding revision:***

*(p. 11, lines 230-237)* *The database search identified 3,746 records. After removal of 1,108 duplicates, 2,638 records underwent title and abstract screening, of which 2,542 were excluded.* *96 full-text reviews were assessed for eligibility, and 84 were excluded.* *The reasons for exclusion were an ineligible phenomenon of interest (n = 27), an ineligible population (n = 21), an ineligible review design (n = 9), and the reviews were on informal carers’ experiences in general rather than experiences of accessing or engaging with mental health services (n = 27). The records excluded at the full-text screening stage were presented in* ***S2 Appendix*** *with reasons for their exclusion to ensure transparency.*

5. The authors may wish to clarify whether the review protocol was registered before or after the search was conducted, as the search was conducted on 9 May 2025, while the protocol is reported as registered on 20 July 2025.

Thank you for this comment. We confirm that the timing of the literature search and protocol registration was accurately reported in the manuscript. The protocol was registered with PROSPERO after the electronic literature search was run but before literature searching was completed and data extraction and subsequent analysis were started. Protocol registration before data extraction is acceptable for prospective registration according to PROSPERO guidance.

**Reviewer #2:** Thank you for the opportunity to review this umbrella review of qualitative studies related to carers experience with mental health care services. The authors should be commended on the depth of work included in this review and the thorough documentation of developing categories and synthesising findings. Overall, that manuscript is methodologically strong.

I have two main concerns, the first being the length of the manuscript. There is a lot of cross-over of findings between the appendix tables and the main text. Can the main text be shortened to a brief high-level summary of findings, with more reference made to the table in the appendix? While there is an extraordinary amount of detail included in this review, it is easy to get lost in the lengthiness of the paper. It is important work and providing a more succinct manuscript would strengthen the clarity of the findings.

Thank you for this helpful comment. We recognise the tension between providing sufficient detail and transparency regarding the review process and findings, while also presenting a concise and easily digestible manuscript. Overall, Reviewer 1 has asked us for more detail while Reviewer 2 has asked us for more brevity. We have done our best to meet both these reasonable requests, but apologise that we may not have fully followed either Reviewer’s steer.

In the current manuscript, the opening paragraph under each synthesised finding is intended to provide a brief, overarching summary of that synthesised finding, followed by more detailed narrative descriptions of the contributing categories. To make this structure clearer for readers, we have added an explanation of the organisation of the Results section (p. 18, lines 310-317).

In addition, to better balance richness with readability, if the Editors permit, we propose to present the overarching summary (i.e. the opening paragraph) of each synthesised finding in a highlighted textbox. This would help readers to quickly identify and understand the key messages of each synthesised finding, while retaining the more detailed category-level narratives for readers who wish to explore the findings in greater depth. We haven’t yet made this change in the manuscript, but would be glad to do so if it fits with journal policies.

***Corresponding revision:***

*(p. 18, lines 310-317) As for the narrative presentations, the opening paragraph under each synthesised finding provides a brief, overarching summary of that synthesised finding. The brief summary is followed by narrative descriptions of each contributing category, with each subsequent paragraph corresponding to one category and elaborating on the specific aspects of carers’ experiences represented within it. Where appropriate, data excerpts or quotations reported in the included reviews are presented to support the narrative synthesis and provide contextual richness while remaining faithful to the original review findings.*

My second concern is the search date of the review as this is now 12 months old, it would benefit from being updated.

Thank you for the comment. We appreciate the importance of maintaining an up-to-date evidence base. This requires review searches to be recent, but also for delays in publication to be avoided. The literature search was conducted well within one year of the original submission of the manuscript, so is within the bounds of standard good practice. Our research team does not have the resources to repeat the search now, and we are mindful that this would also delay the publication of findings. We therefore respectfully think that updating the search is not warranted. The search date has been clearly reported in the manuscript to ensure transparency.

Minor comments:
1) Can the authors initials be included for those who undertook screening and reviewing articles, and who extracted data.

Thank you for the comment. The initials are added to the manuscript (pp.6-7, lines 130-148).

2) Providing additional detail of the quality rating of reviews by using high, moderate, and low is helpful in letting the readers know about which reviews were of more superior quality.

Thank you for the comment. The last column in Table 1 actually reported the quality rating of reviews using high, moderate, and low, but was mistakenly labelled as Dependability and thus caused confusion. The mistake is now corrected (pp. 12-17, Table 1).

3) Can the authors specify how many reviews synthesised and reported information on carers?

Thank you for the comment. The numbers are added to the manuscript (p. 12, lines 264-265).

4) The quality appraisal and certainty assessment could be presented in the same section for better flow.

Thank you for this comment. We have reported these two assessments separately because they occurred at different stages in the research process. Quality assessment of primary papers was undertaken before data synthesis. Assessment of confidence in our synthesised findings followed data synthesis. Our preference is therefore to retain two distinct sections as in our current manuscript, but we are happy to defer to the Journal’s editorial direction on this.

5) In the appendices, it is not clear what ‘verbatim replication of the source reviews’ means i.e. what has been replicated. In the manuscripts that phrasing ‘verbatim excerpts’ has been used. Can this wording be the same, and a brief description of what this is.

Thank you for the comment. We have revised the terminology throughout the supplementary materials to use “verbatim excerpts” consistently (Supplementary Material, pp. 15-54, S3 & S4 Appendices).

To improve clarity, we have also added a brief description explaining that verbatim excerpts refer to “text copied directly from the included reviews without modification, representing the original descriptions of findings reported by the review authors” and serving as the source material for data extraction (p. 7, lines 153-155).

6. PLOS authors have the option to publish the peer review history of their article. If published, this will include your full peer review and any attached files.

Thank you for the information. We are happy for the peer review history of our article to be published in support of open science practices.

We have addressed all the comments and suggestions raised by the Editor and Reviewer(s) and made the corresponding revisions to the manuscript.

Last but not least, during the revision process, we identified an error in the reported number of extractions, which has been corrected from **102** to **105** in the revised manuscript (p. 18, line 306). This reporting error does not affect the underlying data, synthesis, or conclusions.

Thank you again for the Editor’s and Reviewer(s)’ careful consideration and constructive feedback, which have helped us further improve the manuscript. We hope that the revised manuscript will be suitable for further consideration for publication in *PLOS Mental Health*.

Yours sincerely,

Xiaoye Liu
on behalf of all authors
