# Supplementary material for: Informal carers’ experiences of mental health services regarding supporting those they care for and getting support for their own wellbeing: A systematic umbrella review of qualitative evidence
Source: PLOS Ment Health. 2026 Oct 15;3(10):e0000740. doi: 10.1371/journal.pmen.0000740 (PMC13671576; doi:10.1371/journal.pmen.0000740)
Supplement: Peer review history

# Author comment: Author response to Decision Letter 0

**DOI:** 10.1371/journal.pmen.0000740.r001

18 May 2026

Attachment Submitted filename: Response to editors letter_180526.docx

# Peer review: Decision Letter 0

**DOI:** 10.1371/journal.pmen.0000740.r002

**Authors:** Karli Montague-Cardoso

16 Jun 2026

PMEN-D-26-00246

Informal carers’ experiences of mental health services regarding supporting those they care for and getting support for their own wellbeing: a systematic umbrella review of qualitative evidence

PLOS Mental Health

Dear Dr. Lloyd-Evans,

Thank you for submitting your manuscript to PLOS Mental Health. I am sorry for the delay in reaching a decision, and thank you for resubmitting this piece. After careful consideration of the reviewer reports, we feel that the paper has merit but does not fully meet PLOS Mental Health’s publication criteria as it currently stands. Therefore, we invite you to submit a revised version of the manuscript that addresses the points raised during the review process. You can find all of the comments from the reviewers below.

Please submit your revised manuscript by Aug 15 2026 11:59PM. If you will need more time than this to complete your revisions, please reply to this message or contact the journal office at mentalhealth@plos.org. When you're ready to submit your revision, log on to https://www.editorialmanager.com/pmen/ and select the 'Submissions Needing Revision' folder to locate your manuscript file.

Please include the following items when submitting your revised manuscript:

- A letter that responds to each point raised by the editor and reviewer(s). You should upload this letter as a separate file labeled 'Response to Reviewers'.
- A marked-up copy of your manuscript that highlights changes made to the original version. You should upload this as a separate file labeled 'Revised Manuscript with Track Changes'.
- An unmarked version of your revised paper without tracked changes. You should upload this as a separate file labeled 'Manuscript'.

Guidelines for resubmitting your figure files are available below the reviewer comments at the end of this letter.

As the corresponding author, your ORCID iD is verified in the submission system and will appear in the published article. PLOS supports the use of ORCID, and we encourage all coauthors to register for an ORCID iD and use it as well. Please encourage your coauthors to verify their ORCID iD within the submission system before final acceptance, as unverified ORCID iDs will not appear in the published article. *Only*  the individual author can complete the verification step; PLOS staff *cannot*  verify ORCID iDs on behalf of authors.

We look forward to receiving your revised manuscript.

Kind regards,

Dr Karli Montague-Cardoso

Staff Editor

PLOS Mental Health

**Journal Requirements:**

1. Please provide a complete Data Availability Statement in the submission form, ensuring you include all necessary access information or a reason for why you are unable to make your data freely accessible. If your research concerns only data provided within your submission, please write "All data are in the manuscript and/or supporting information files" as your Data Availability Statement.

2. As required by our policy on Data Availability, please ensure your manuscript or supplementary information includes the following:

A numbered table of all studies identified in the literature search, including those that were excluded from the analyses.

For every excluded study, the table should list the reason(s) for exclusion.

If any of the included studies are unpublished, include a link (URL) to the primary source or detailed information about how the content can be accessed.

A table of all data extracted from the primary research sources for the systematic review and/or meta-analysis. The table must include the following information for each study:

Name of data extractors and date of data extraction

Confirmation that the study was eligible to be included in the review.

All data extracted from each study for the reported systematic review and/or meta-analysis that would be needed to replicate your analyses.

If data or supporting information were obtained from another source (e.g. correspondence with the author of the original research article), please provide the source of data and dates on which the data/information were obtained by your research group.

If applicable for your analysis, a table showing the completed risk of bias and quality/certainty assessments for each study or outcome.  Please ensure this is provided for each domain or parameter assessed. For example, if you used the Cochrane risk-of-bias tool for randomized trials, provide answers to each of the signalling questions for each study. If you used GRADE to assess certainty of evidence, provide judgements about each of the quality of evidence factor. This should be provided for each outcome.

An explanation of how missing data were handled.

This information can be included in the main text, supplementary information, or relevant data repository. Please note that providing these underlying data is a requirement for publication in this journal, and if these data are not provided your manuscript might be rejected.

If the reviewer comments include a recommendation to cite specific previously published works, please review and evaluate these publications to determine whether they are relevant and should be cited. There is no requirement to cite these works unless the editor has indicated otherwise.

**Additional Editor Comments (if provided):**

[Note: HTML markup is below. Please do not edit.]

Reviewers' comments:

Reviewer's Responses to Questions

**Comments to the Author**

1. Does this manuscript meet PLOS Mental Health’s publication criteria?

Reviewer #1: Partly

Reviewer #2: Yes

**********

2. Has the statistical analysis been performed appropriately and rigorously?

Reviewer #1: N/A

Reviewer #2: Yes

**********

3. Have the authors made all data underlying the findings in their manuscript fully available (please refer to the Data Availability Statement at the start of the manuscript PDF file)?

The PLOS Data policy

Reviewer #1: Yes

Reviewer #2: Yes

**********

4. Is the manuscript presented in an intelligible fashion and written in standard English?

Reviewer #1: Yes

Reviewer #2: Yes

**********

**Reviewer #1:**  This manuscript presents a systematic umbrella review of qualitative review evidence on informal carers’ experiences of mental health services, both in supporting the individuals they care for and in seeking support for their own wellbeing. The topic is important and clearly relevant to PLOS Mental Health. The manuscript is generally well organised, follows a recognised umbrella review approach, and attempts to use JBI-informed methods, quality appraisal and ConQual confidence assessment. However, I think the manuscript requires substantial revision before it can be considered for publication. My main concerns relate to methodological transparency, consistency in reporting, the handling of heterogeneous review types, the assessment of confidence, and the need for a more nuanced discussion of implications across different mental health service contexts.

Major comments

1. There is an inconsistency regarding language restrictions.

In the eligibility criteria, the authors state that “There were no restrictions on language, geographic location, or date of the reviews.” However, the results later state that all included reviews were published in English. This needs clarification. If no language restriction was applied, the authors should explain whether non-English reviews were identified and excluded for other reasons, or whether none were retrieved. If the search strategy or databases effectively limited retrieval to English-language publications, this should be acknowledged as a limitation. At present, the wording gives the impression of a broader search than may actually have been achieved.

2. The title/abstract screening procedure needs stronger justification.

The manuscript reports that title and abstract screening was undertaken by one reviewer, with a second reviewer double-screening only 10% of records, although agreement was high. For a systematic umbrella review, this may raise concerns about missed eligible reviews. The authors should justify this approach more clearly and consider providing additional reassurance, for example by reporting the number of records checked by the second reviewer, disagreements identified, and whether any calibration was conducted before screening. If feasible, a larger proportion of records could be double-screened.

3. The inclusion of different types of reviews requires further explanation.

The eligibility criteria refer to published systematic reviews or qualitative meta-syntheses, but the included studies also contain one integrative literature review and one narrative review. The authors should clarify why these review types were eligible and how they meet the threshold for inclusion in an umbrella review. This is particularly important because the methodological standards and reporting quality of narrative/integrative reviews may differ from those of systematic qualitative syntheses. A sensitivity analysis or a clearer justification of their influence on the synthesis would strengthen the manuscript.

4. Overlap between primary studies across included reviews should be assessed more explicitly.

The authors acknowledge in the limitations that overlapping primary studies across included reviews may overrepresent certain findings. However, this issue is only briefly mentioned and does not appear to be systematically assessed. In an umbrella review, overlap can materially affect the interpretation of synthesised findings. The authors should consider mapping primary studies across the included reviews, reporting the degree of overlap, and discussing whether some findings are disproportionately driven by the same primary studies appearing in multiple reviews.

5. The confidence assessment appears too strong and needs more cautious interpretation.

The abstract states that the findings were rated as high confidence. However, the manuscript also notes important limitations, including inconsistent reporting of primary study context, limited demographic information, predominance of high-income country evidence, and disproportionate focus on acute or hospital-based contexts. The authors should explain more clearly how these limitations were incorporated into the ConQual assessment. The current “high confidence” framing may overstate the certainty and transferability of the findings.

6. The contribution beyond existing reviews should be made clearer.

The manuscript argues that no comprehensive synthesis has integrated the existing qualitative review literature into a single high-level evidence base. This is a reasonable rationale. However, many of the findings, such as carers’ exclusion from decision-making, lack of information, emotional distress, and difficulty navigating services, are already well established. The authors should more explicitly articulate what new conceptual, methodological, or service-level insight is generated by this umbrella review. For example, the manuscript could better explain whether the review identifies a cross-cutting pathway of carers’ experiences across different service stages, or whether it reveals gaps in existing service models.

7. The discussion of service implications is currently rather general.

The conclusion recommends improving service accessibility, raising public awareness, providing guidance, facilitating carer involvement, and enhancing professional competence. These are important but broad recommendations. The authors should develop more specific implications for different service contexts, for example inpatient care, compulsory admission, community mental health services, crisis care, discharge planning, and services designed primarily for carers.

Minor comments

1. There are some typographical and formatting issues that should be corrected, for example “expriences” in Table 1 and occasional spacing issues such as “experiences.A summary table”.

2. The phrase “the rest reviews” should be revised to “the remaining reviews” or “the rest of the reviews.”

3. The authors should ensure consistent use of “synthesised” and “synthesized” throughout the manuscript.

4. It would be helpful to include more detail on the excluded full-text reviews and reasons for exclusion, either in the main manuscript or supplementary materials.

5. The authors may wish to clarify whether the review protocol was registered before or after the search was conducted, as the search was conducted on 9 May 2025, while the protocol is reported as registered on 20 July 2025.

**Reviewer #2:**  Thank you for the opportunity to review this umbrella review of qualitative studies related to carers experience with mental health care services. The authors should be commended on the depth of work included in this review and the thorough documentation of developing categories and synthesising findings. Overall, that manuscript is methodologically strong.

I have two main concerns, the first being the length of the manuscript. There is a lot of cross-over of findings between the appendix tables and the main text. Can the main text be shortened to a brief high-level summary of findings, with more reference made to the table in the appendix?

While there is an extraordinary amount of detail included in this review, it is easy to get lost in the lengthiness of the paper. It is important work and providing a more succinct manuscript would strengthen the clarity of the findings.

My second concern is the search date of the review as this is now 12 months old, it would benefit from being updated.

Minor comments:

1) Can the authors initials be included for those who undertook screening and reviewing articles, and who extracted data.

2) Providing additional detail of the quality rating of reviews by using high, moderate, and low is helpful in letting the readers know about which reviews were of more superior quality.

3) Can the authors specify how many reviews synthesised and reported information on carers? Page 10, line 221-223.

4) The quality appraisal and certainty assessment could be presented in the same section for better flow.

5) In the appendices, it is not clear what ‘verbatim replication of the source reviews’ means i.e. what has been replicated. In the manuscripts that phrasing ‘verbatim excerpts’ has been used. Can this wording be the same, and a brief description of what this is.

**********

what does this mean?). If published, this will include your full peer review and any attached files.

**Do you want your identity to be public for this peer review?**  If you choose “no”, your identity will remain anonymous but your review may still be made public.

For information about this choice, including consent withdrawal, please see our Privacy Policy

Reviewer #1: No

Reviewer #2: No

**********

[NOTE: If reviewer comments were submitted as an attachment file, they will be attached to this email and accessible via the submission site. Please log into your account, locate the manuscript record, and check for the action link "View Attachments". If this link does not appear, there are no attachment files.]

**Figure Resubmissions:**

# Author comment: Author response to Decision Letter 1

**DOI:** 10.1371/journal.pmen.0000740.r003

4 Sep 2026

Attachment Submitted filename: Response to Reviewers.docx

# Editor report: Decision Letter 1

**DOI:** 10.1371/journal.pmen.0000740.r004

**Authors:** Karli Montague-Cardoso

17 Sep 2026

Informal carers’ experiences of mental health services regarding supporting those they care for and getting support for their own wellbeing: a systematic umbrella review of qualitative evidence

PMEN-D-26-00246R1

Dear Professor Lloyd-Evans,

We are pleased to inform you that your manuscript 'Informal carers’ experiences of mental health services regarding supporting those they care for and getting support for their own wellbeing: a systematic umbrella review of qualitative evidence' has been provisionally accepted for publication in PLOS Mental Health.

Before your manuscript can be formally accepted you will need to complete some formatting changes, which you will receive in a follow up email. A member of our team will be in touch with a set of requests.

Please note that your manuscript will not be scheduled for publication until you have made the required changes, so a swift response is appreciated.

IMPORTANT: The editorial review process is now complete. PLOS will only permit corrections to spelling, formatting or significant scientific errors from this point onwards. Requests for major changes, or any which affect the scientific understanding of your work, will cause delays to the publication date of your manuscript.

If your institution or institutions have a press office, please notify them about your upcoming paper to help maximize its impact. If they'll be preparing press materials, please inform our press team as soon as possible -- no later than 48 hours after receiving the formal acceptance. Your manuscript will remain under strict press embargo until 2 pm Eastern Time on the date of publication. For more information, please contact mentalhealth@plos.org.

Thank you again for supporting Open Access publishing; we are looking forward to publishing your work in PLOS Mental Health.

Best regards,

Karli Montague-Cardoso

Staff Editor

PLOS Mental Health

***********************************************************

Reviewer Comments (if any, and for reference):
